# The neuroprotective effects of glucagon-like peptide 1 in Alzheimer’s and Parkinson’s disease: An in-depth review

**DOI:** 10.3389/fnins.2022.970925

**Published:** 2022-09-01

**Authors:** Niklas Reich, Christian Hölscher

**Affiliations:** ^1^Biomedical and Life Sciences Division, Faculty of Health and Medicine, Lancaster University, Lancaster, United Kingdom; ^2^Neurology Department, Second Associated Hospital, Shanxi Medical University, Taiyuan, China; ^3^Henan University of Chinese Medicine, Academy of Chinese Medical Science, Zhengzhou, China

**Keywords:** GLP-1, Alzheimer’s disease, Parkinson’s disease, neuroinflammation, amyloid beta, mitochondrial dysfunction, brain glucose hypometabolism, insulin resistance

## Abstract

Currently, there is no disease-modifying treatment available for Alzheimer’s and Parkinson’s disease (AD and PD) and that includes the highly controversial approval of the Aβ-targeting antibody aducanumab for the treatment of AD. Hence, there is still an unmet need for a neuroprotective drug treatment in both AD and PD. Type 2 diabetes is a risk factor for both AD and PD. Glucagon-like peptide 1 (GLP-1) is a peptide hormone and growth factor that has shown neuroprotective effects in preclinical studies, and the success of GLP-1 mimetics in phase II clinical trials in AD and PD has raised new hope. GLP-1 mimetics are currently on the market as treatments for type 2 diabetes. GLP-1 analogs are safe, well tolerated, resistant to desensitization and well characterized in the clinic. Herein, we review the existing evidence and illustrate the neuroprotective pathways that are induced following GLP-1R activation in neurons, microglia and astrocytes. The latter include synaptic protection, improvements in cognition, learning and motor function, amyloid pathology-ameliorating properties (Aβ, Tau, and α-synuclein), the suppression of Ca^2+^ deregulation and ER stress, potent anti-inflammatory effects, the blockage of oxidative stress, mitochondrial dysfunction and apoptosis pathways, enhancements in the neuronal insulin sensitivity and energy metabolism, functional improvements in autophagy and mitophagy, elevated BDNF and glial cell line-derived neurotrophic factor (GDNF) synthesis as well as neurogenesis. The many beneficial features of GLP-1R and GLP-1/GIPR dual agonists encourage the development of novel drug treatments for AD and PD.

## Introduction

As a major peptide hormone and growth factor, glucagon-like peptide 1 (GLP-1) regulates several physiological processes in the body and brain. GLP-1 is produced in enteroendocrine cells of the lower gastrointestinal tract and continuously liberated at a low basal level. It is also produced in other cells and organs in the body such as the brain (Yang X. et al., 2022). Known as the incretin effect, GLP-1 and its synergetic sister hormone glucose-dependent insulinotropic polypeptide (GIP), are rapidly released by lower enteroendocrine L-cells (GLP-1) or upper enteroendocrine K-cells (GIP) in the gut following food intake and rising circulatory glucose levels to augment the glucose-stimulated release of insulin by pancreatic β cells. Other associated effects of GLP-1 include the inhibition of glucagon secretion by pancreatic α cells, reduced gastric emptying and intestinal transit, the suppression of appetite, enhanced satiety and further functions in other tissues (see Drucker, 2018; Nauck and Meier, 2018; Zhao et al., 2021a).

In gastric L-cells, the bioactive forms of GLP-1, GLP_7–37_ (glycine-extended GLP-1), and GLP_7–37 amide_, are cleaved from its precursor (pre)proglucagon by prohormone convertase (PC)1 and PC3, which co-generates GLP-2 and oxyntomodulin. On the other hand, GIP is synthesized as a pro-peptide (pro-GIP) that is post-translationally processed to GIP in K-cells. Notably, proglucagon is cleaved in an alternative fashion by PC2 in pancreatic α cells to yield glucagon, but not GLP-1/2. Once GLP-1 is released, the peptide acts on GLP-1 receptors (GLP-1Rs) that are widely distributed in peripheral tissue, including the gut, stomach, pancreas, kidneys, heart, adipose cells, bones, and blood vessels (Vrang and Larsen, 2010; Drucker, 2018; Nauck and Meier, 2018).

Importantly, while blood-borne GLP-1 readily crosses the blood brain barrier (BBB) (Kastin et al., 2002), incretins and their receptors are expressed in the central nervous system (CNS). A major source of preproglucagon and, thus, GLP-1, is found in PC1/3-expressing neurons in the caudal area of the medial nucleus of the solidary tract (NTS) and, partially, the area postrema, which both belong to the dorsal vagal complex (DVC). Preproglucagon-containing cell bodies were also identified in the granular cell layer of the olfactory bulb, dorsal and ventral medulla and lumbar sacral spinal cord. In this context, the activity of preproglucagon-expressing neurons in the NTS is stimulated by afferent vagal inputs that relay satiety signals from the periphery to the brain, such as gastric distention, the induction of peripheral GLP-1Rs or the release of the satiety-associated hormones cholecystokinin and leptin. Furthermore, NTS neurons form proglucagon and/or GLP-1-positive projections toward the olfactory bulb, various hypothalamic nuclei, the bed nucleus of the stria terminalis, lateral and medial septal nuclei, the amygdaloid complex, the septohippocampal region, nucleus accumbens and, more sparsely, medullary reticular formation, dorsal motor nucleus of the vagus and cortex. In contrast to the NTS-localized production and distribution of GLP-1, the GLP-1R is widely distributed in the CNS. *In vivo*, the enriched immunoreactivity or transcription of the GLP-1R was detected in neurons of the DVC, paraventricular and posterior thalamic nuclei, various hypothalamic regions, ventral, posterodorsal and interpeduncular tegmental areas, the periaqueductal gray and superior colliculus, while medium to low levels of GLP-1Rs were found in the posterior/caudal hippocampus, the hippocampal CA1, temporal/cerebral cortex, striatum, substantia nigra (SN) [which included tyrosine hydroxylase (SN)-expressing dopaminergic neurons (Elabi et al., 2021)], locus coeruleus, preoptic area, parabrachial nuclei, lateral septum, lateral habenula; zona incerta; substantia innominate, subfornical organ, interpenduncular nucleus, superior colliculus, ventral pallium; nucleus basalis of Meynert, central gray (especially medial), amygdala, spinal cord, the bed nuclei of the stria terminalis, the shell of the nucleus accumbens and the dorsal raphe nuclei. Besides neurons, astrocytes and microglia express GLP-1Rs (see Vrang and Larsen, 2010 for a general overview) (Merchenthaler et al., 1999; Iwai et al., 2006; Lee et al., 2011a; Darsalia et al., 2013; Trapp and Richards, 2013; Trapp and Cork, 2015). Other studies using rodents or primates with immunofluorescent reporter GLP-1Rs verify the expression of GLP-1Rs by pyramidal (glutamergic), GABAergic and catecholaminergic neurons in feeding, energy homeostasis, fear, stress, motivation/reward, learning plus memory as well as Alzheimer’s disease (AD) and Parkinson’s Disease (PD)-associated key brain areas, such as the hippocampus, hypothalamus, cortex, striatum, amygdala, SNpc, or ventral tegmental area (VTA) (Cork et al., 2015; Heppner et al., 2015; Graham et al., 2020). Prominent in neurons, but not glial cells, GIP synthesis is more distributed in the CNS compared to GLP-1 and was identified in the hippocampal CA1-3, granule cell layer and neurogenic niche of the dentate gyrus, thalamus, cerebral cortex, cerebellum (Purkinje cells) and brainstem. GIP immunoreactivity was further observed in the olfactory bulb, basal ganglia (including the striatum, nucleus accumbens, ventral pallidum and SN), amygdala, lateral septal nucleus as well as multiple hypothalamic nuclei. Moreover, GIPRs are present in pyramidal cortical and hippocampal, DG granular and progenitor plus cerebellar neurons and further brain regions (see Zhang and Holscher, 2020 for more information about GIP) (Nyberg et al., 2005, 2007).

**FIGURE 1:**
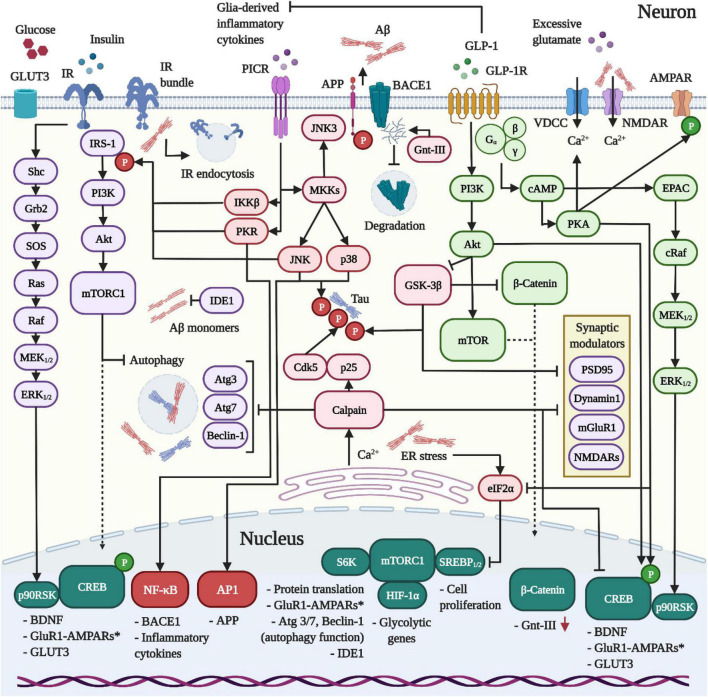
Dynamics between IR, GLP-1R and PICR-signaling in neurons. **1** The release of pro-inflammatory cytokines and the activation of PICRs on neurons induce the kinases IKKβ, PKR, and JNK to trigger the inhibitory Ser-phosphorylation of IRS-1 and neuronal insulin resistance in AD and PD. Aβ was further shown to drive IR clustering and endocytosis. In addition, Aβ provokes intracellular Ca^2+^ accumulation by external (VDCCs/NMDARs) and internal means (ER). The latter reinforces the desensitization of the insulin pathway, blocks protein translation (eIF2α/mTORC1 pathway) and activates the Ca^2+^-sensitive calpain to impair autophagy, interfere with the synaptic function and promote the hyperphosphorylation of Tau by cleaving p35 into the Cdk5-activating binding partner p25. Further consequences of inflammation and insulin resistance include reduced IDE1 expression, enhanced APP and BACE1 expression, Aβ overproduction and amassment, the loss of neuroprotective PI3K/Akt and CREB signaling, GSK-3β hyperactivity and concomitant Tau hyperphosphorylation. Crucially, the impairment of the Akt/mTor pathway following insulin resistance impedes the expression of glycolytic enzymes, thus enforcing bioenergetic impairments and glucose hypometabolism. **2** In contrast to the IR, the GLP-1R does not desensitize in neurons. When activated, the GLP-1R stimulates PI3K/Akt/mTORC1, cAMP/PKA, MEK/ERK, and CREB/BDNF-signaling to ameliorate the Aβ (section “GLP-1R agonists are neuroprotective and prevent amyloid beta accumulation *in vivo”*) and Tau (section “GLP-1R mimetics suppress Tau hyperphosphorylation and aggregation during AD”) pathologies through various mechanisms, suppress excessive Ca^2+^ influx and ER stress (not shown; details in sections “GLP-1 mimetics suppress Ca2+ deregulation by amyloid beta and excitotoxicity” and “GLP-1 analogues counteract endoplasmic reticulum stress”), restore insulin signaling (section “Insulin resistance and the neuronal energy metabolism”) by aiding the clearance of Aβ, normalize the autophagy function by raising the expression of autophagy modulators via mTORC1 (Atg3, Atg7, Beclin-1) (section “Autophagy and mitophagy”), elevate neurogenesis (section “GLP-1R agonists promote neurogenesis”) and promote the synaptic function, plasticity and memory (section “Pro-cognitive effects”). Importantly, the activation of GLP-1R on microglia and astrocytes induces the anti-inflammatory M2 phenotype and suppresses inflammatory cytokine production (not shown; see section Inflammation), thus preventing insulin resistance.

As G_*q*α_-recruiting and G-protein coupled receptors (GPCR), the downstream signaling pathways of GLP-1Rs and GIPRs parallel those of insulin and lead to the induction of the neuroprotective phosphoinositide 3-Kinase (PI3K)/Akt (also known as protein kinase B)/mammalian target of rapamycin (mTor), cyclic adenosine monophosphate (cAMP)/protein kinase A (PKA)/cAMP-response element binding protein (CREB) as well as Raf/mitogen-activated protein kinase (MEK)_1/2_/extracellular signal-regulated kinase (ERK) pathways (for the GLP-1R pathways, see also Figure 1; Hölscher, 2020; Zhang and Holscher, 2020).

Given the role of GLP-1 in glycaemic control, the anti-apoptotic, growth-stimulating and insulin secretion-promoting effects on pancreatic β-cells, the ability to re-sensitize the cellular insulin-signaling, minor side effects, good tolerance by normoglycemic patients and the fact that GLP-1 does not desensitize, synthetic GLP-1R agonists have been on the market for decades to treat type 2 diabetes mellitus (T2DM) or, more recently, to improve weight loss (Drucker, 2018). Due to renal clearance and the cleavage of GLP-1 by dipeptidyl peptidase-4, GLP-1 exhibits a short half-life time (t_1/2_) of 1–2 min. Thus, multiple proteolysis-resistant GLP-1 analogs, either short- or long-lasting, have been developed for the clinical use. This includes the structurally similar liraglutide (once daily, t_1/2_ = 13 h), semaglutide (once weekly, t_1/2_ = 165–185 h), dulaglutide (once weekly, t_1/2_ = 90 h), the more dissimilar Exendin-4 (Exenatide; twice daily, t_1/2_ = 2.4 h), which is derived from the saliva of Gila lizards and shows ∼ 53% sequence homology with GLP-1, as well as Lixisenatide (once daily, t_1/2_ = 3 h) (Andreasen et al., 2021). Moreover, novel GLP-1R/GIPR dual agonists have been introduced for the clinical treatment of hyperglycaemia and T2DM, which show synergistic and superior metabolic effects compared to single incretin receptor agonists (Finan et al., 2013; Frias et al., 2017, 2018). In addition, we and others have reviewed the promising effects of GLP-1, GIP, and GLP-1R/GIPR dual agonists in animal and clinical studies for the treatment of various neurodegenerative diseases (see Hölscher, 2020, 2022b for an overview).

Insulin desensitization is found in the brains of patients with AD or PD (Talbot et al., 2012; Freiherr et al., 2013), and GLP-1 receptor agonists can re-sensitize insulin signaling. Therefore, the use of incretins to normalize insulin signaling in the brain has been tested in animal models of disease and in patients with AD and PD (see section “Clinical trials show good protective effects in patients with AD or PD”) (Hölscher, 2022b). In the context of AD and PD, we have investigated the effects of five GLP-1R/GIPR dual agonists with various modifications, termed DA1-DA5. Of these, the cerebral uptake of DA4-JC4 and DA5-CH was enhanced by the addition of a cell-penetrating sequence (poly-lysine) (Hölscher, 2022b). Crucially, all forementioned GLP-1 analogs and GLP-1R/GIPR dual agonists cross the BBB *in vivo*, albeit at varying rates. In comparison, DA4-JC4 (t_1/2_ = 151.94 min) and, similarly, DA5-CH showed the highest BBB penetration rates, followed by medium BBB translocation of Exendin-4, lixisenatide and DA3-CH as well as poor brain influx of the lipidated peptides liraglutide, semaglutide, DA1-JC as well as the PEGylated DA2 and NLY01, a variant of Exendin-4 with prolonged t_1/2_ (Li et al., 2020a; Salameh et al., 2020; Zhang et al., 2020; Lv et al., 2021). We confirmed that GLP-1R/GIPR dual agonists demonstrated superior neuroprotective effects compared to other synthetic GLP-1 analogs in AD (Maskery et al., 2020; Salles et al., 2020) and PD (Yuan et al., 2017; Feng et al., 2018; Zhang and Holscher, 2020; Lv et al., 2021; Zhang L.Y. et al., 2021) animal models, correlating with their greater ability to cross the BBB. A detailed review is found here (Hölscher, 2022b).

Oxyntomodulin is a natural dual GLP-1/glucagon receptor agonist (Vrang and Larsen, 2010). We and others tested a range of protease-resistant oxyntomodulin analogs (Clemmensen et al., 2013) in different animal models of AD, PD, or stroke (Liu et al., 2015b; Li et al., 2017; Wang et al., 2020; Yang G. Z. et al., 2022). While they showed good protective effects, there was no improvement over single GLP-1 receptor agonists, suggesting that the glucagon receptor binding site does not contribute to the neuroprotective effects (Jalewa et al., 2016). We furthermore tested triple GLP-1/GIP/glucagon receptor agonists (Finan et al., 2015) and did not find an improvement over dual GLP-1/GIP receptor agonists (Feng et al., 2018; Li et al., 2018, 2020b; Tai et al., 2018).

Herein, given the success of GLP-1 analogs in phase II clinical trials (see section “Clinical trials show good protective effects in patients with AD or PD” for details), we will illustrate the manifold neuroprotective, cognition and motor-enhancing signaling pathways that are induced by the GLP-1R in detail and place them in the context with AD and PD.

## Pro-cognitive effects

### GLP-1 receptor analogs protect synapses and drive synaptogenesis

Extensive synapse loss, already apparent during the mild cognitive impairment (MCI) preclinical stage, is one of the earliest pathologic alterations in AD (John and Reddy, 2021). Indeed, an exosome investigation revealed that the synaptic protein levels decreased years before clinical symptoms manifested and inversely correlated with cognition in AD and frontotemporal dementia patients, whereas Amyloid Beta (Aβ)_1–42_ or phospho-Thr^181^-Tau did not (Goetzl et al., 2016). The accumulation of amyloids in AD may harm synapses by various means, such as:

(i) The interaction of secreted (extracellular) Aβ with α-amino-3-hydroxy-5-methyl-4-isoxazolepropionic acid receptors (AMPARs) on postsynaptic terminals, leading to AMPAR endocytosis, ubiquitination and degradation to impair long-term potentiation (LTP), elongate long-term depression (LTD), and elicit the loss of dendritic spines.
(ii) The Aβ-induced intracellular Ca^2+^ amassment across N-methyl-D-aspartate receptors (NMDARs), voltage-dependent Ca^2+^ channel (VDCCs) and internal Ca^2+^ stores (see also section “GLP-1 mimetics suppress Ca2+ deregulation by amyloid beta and excitotoxicity”) that provokes the calcineurin-mediated phosphorylation of cytoskeleton components and spine loss as well as oxidative stress. The latter, in turn, further stimulates the activity of beta site amyloid precursor protein (APP) cleaving enzyme 1 (BACE1) to encourage the Aβ-generating cleavage of APP.
(iii) The accumulation of hyperphosphorylated and microtubule-detached Tau at dendrites (as accelerated by oligomeric Aβ), which may interfere with kinesin/dynein-mediated transport, synaptic AMPAR insertion and, thus, postsynaptic currents.
(iV) The interruption of the mitochondrial function by Aβ (elaborated in section “Oxidative stress and mitochondrial dysfunction”) and impaired mitochondrial transport to synapses due to Tau pathology, resulting in ATP-depletion and synaptic degeneration (see John and Reddy, 2021).

In this context, GLP-1R activation has repeatedly shown synaptoprotective properties. Various GLP-1R or GLP-1R/GIPR dual agonists prevented presynaptic synaptophysin and postsynaptic density protein 95 (PSD-95)/drebrin loss in the brains of mice injected with Aβ oligomers (Batista et al., 2018) as well as multiple hippocampal and cortical regions of APP/presenilin-1 (PS1) (McClean et al., 2011, 2015; Lourenco et al., 2013; McClean and Holscher, 2014a,b; Panagaki et al., 2018), 3 × Tg (APP_*swe*_, Tau_*P30*1L_, and PS1_*M*146V_) (Cai et al., 2021; Zheng et al., 2021) or 5xFAD (APP_*swe*_, APP_*I71*6V_, APP_*V71*7I_, PS1_1M146L/1L286V_) (An et al., 2019; Park et al., 2021) AD rodents. This further includes synaptic protection in the i.c.v. streptozotocin (STZ)-microinjected AD rat model (Li et al., 2020a), the hippocampus, frontal cortex, and amygdala of Aβ-infused primates (Batista et al., 2018) and the brains of 1-methyl-4-phenyl-1,2,3,6-tetrahydropyridine (MPTP)-injected PD animals (Cao et al., 2016; Feng et al., 2018). Indeed, *in vivo* ultra-structural investigations confirm that the administration of liraglutide or Exendin-4 partially or fully reversed the decline in synaptic vesicle numbers and postsynaptic areas, swollen membranes, enlarged synaptic clefts and more (Qi et al., 2016; An et al., 2019; Zheng et al., 2021). In addition, GLP-1R activation preserved the levels of the dendrite marker microtubule-associated protein 2 (MAP2) as well as dendritic spine numbers in AD animal models (Cai et al., 2021; Park et al., 2021).

Mechanistically, as shown in cultured hippocampal neurons, liraglutide rescued the Aβ oligomer-driven downregulation of synaptic proteins and synapse densities in a GLP-1R and cAMP/PKA-dependent manner (Batista et al., 2018). In this context, PKA selectively phosphorylates AMPAR-GluR1 subunits at Ser^845^ to promote their synaptic incorporation and sustain LTP in the cornu ammonis 1 (CA1) (Figure 1; Esteban et al., 2003; Fang et al., 2003; Oh et al., 2006; Lee H.K. et al., 2010). Interestingly, cAMP/PKA-signaling, as induced by the GLP-1 analog, also weakened the interaction of applied oligomeric Aβ with neuronal synapses, similar to insulin. This mechanism might contribute to synaptoprotection (Batista et al., 2018). Besides Aβ, liraglutide maintained GAP43 expression, a growth cone component found at the tips of axons, in H_2_O_2_-challenged RCG-5 retinal ganglion cells. The GLP-1 mimetic reduced axonal degeneration by protecting axonal mitochondria from oxidative damage, while preventing the aberrant enhancement of autophagy/mitophagy (Ma et al., 2017).

In addition to protecting synapses from amyloids and oxidative stress, GLP-1 directly supports neurite growth and synaptogenesis. In PC12, SH-SY5Y and adult sensory neurons, GLP-1R activation was shown to stimulate the cytoskeletal actin/tubulin polymerisation to elicit neurite multiplication, branching and outgrowth *in vitro* (Perry et al., 2002b; Liu et al., 2006; Luciani et al., 2010; Kan et al., 2012), comparable to NGF (Perry et al., 2002b). A GLP-1 mimetic further promoted the expression of various synaptic proteins, including synapsin 1, synaptophysin and PSD-95, in SH-SY5Y cells (Yang et al., 2020) and PSD-95 in the neocortex of adult mice (Ohtake et al., 2014). As we describe elsewhere (section “GLP-1R agonists promote neurogenesis”), these pro-neuritic and pro-synaptic effects are the result of (cAMP/PKA-supported) CREB activation and brain derived neurotrophic factor (BDNF) expression by GLP-1 mimetics.

### GLP-1 enhances hippocampal synaptic plasticity

There is clear evidence that the GLP-1R modulates both pre- and postsynaptic plasticity. Early *in vivo* studies in healthy animals demonstrated that GLP-1R activation led to a spontaneous and rapid enhancement of hippocampal firing (1 s), followed by a steady decline in the neuronal activity. Given that a non-NMDA-glutamate receptor inhibitor blocked these effects, it is implied that GLP-1R agonists modulate glutamatergic signaling in the hippocampus (Oka et al., 1999).

Generally, in pancreatic β cells and neurons, GLP-1 elicits insulin or neurotransmitter release, respectively, *via* (i) the cAMP/PKA-mediated opening of L-type VDCCs and Ca^2+^ influx, (ii) membrane depolarisation triggered by the PKA-induced inhibition of voltage-dependent potassium channels and possibly (iii) the PKA/Epac-facilitated priming of insulin or neurotransmitter-containing vesicles for their release upon Ca^2+^ accumulation (see Liu and Pang, 2016; Hölscher, 2022a for an in depth review of the hippocampal synaptic regulation by GLP-1).

Importantly, in a GLP-1R-dependent manner, long-term treatment of AD-like animals with incretin mimetics did not affect the baseline excitatory postsynaptic potentials, but facilitated the induction and maintenance of hippocampal LTP. These improvements were also observed in healthy, age-matched and GLP-1 analog-injected wilt-type (WT) animals, suggesting that GLP-1 enhances LTP even in the absence of neuropathological alterations (Gault and Holscher, 2008; McClean et al., 2010, 2011, 2015; Wang et al., 2010; Gengler et al., 2012; Han et al., 2013; Cai et al., 2014; McClean and Holscher, 2014a,b). Similar LTP-enhancing effects were reported for GLP-1R/GIPR dual agonists (Cao et al., 2018; Maskery et al., 2020; Cai et al., 2021). In contrast, the effects on paired pulse facilitation (PPF), which indicates presynaptic neurotransmitter release, were limited. PPF was not investigated (Gault and Holscher, 2008; McClean et al., 2010; Wang et al., 2010; Han et al., 2013) or was not altered to begin within Aβ-based animal models (Cai et al., 2014; McClean and Holscher, 2014a,b; McClean et al., 2015), albeit two studies reported that GLP-1 analogies elevated PPF in APP/PS1 mice (McClean et al., 2011) and in 18 month-old rodents (Gengler et al., 2012). In turn, GLP-1R knockout mice displayed grave impairments in LTP induction and short interval PPF (25 ms) (Abbas et al., 2009).

Moreover, GLP-1_9–36_*^amide^* and Val8-GLP-1 were shown to normalize the Aβ-induced deficits in LTP induction, postsynaptic excitatory and inhibitory currents as well as changes in LTD in hippocampal slices (Ma et al., 2012; Wang et al., 2013). These LTP improvements were mediated by preventing Aβ-induced Ca^2+^-overload and calpain activation (Figure 1 and details in section “GLP-1 mimetics suppress Ca2+ deregulation by amyloid beta and excitotoxicity”) (Wang et al., 2013) and by improving the mitochondrial function, oxidative stress and Akt/glycogen synthase kinase-3β (GSK-3β)-signaling (as expanded on in section “Oxidative stress and mitochondrial dysfunction”) (Ma et al., 2012).

Notably, the Aβ-associated induction of GSK-3β, as suppressed by GLP-1R/PI3K/Akt-signaling in the hippocampus (Cai et al., 2014; Qi et al., 2016; Cao et al., 2018; Wang et al., 2018; Zhou et al., 2019), provokes the loss of GluR1-AMPARs as well as dendritic and postsynaptic degeneration (Llorens-Martin et al., 2013). The administration of Exendin-4 did not alter glutamate receptor expression nor GluR2-AMPA and NR1-NMDA trafficking, yet promoted the synaptic insertion of GluR1 subunits of AMPA receptors and PSD-95 in the neocortex of both saline and i.c.v. Aβ oligomer-injected adult mice. Exendin-4 further upregulated CREB activity and subsequent BDNF synthesis. Furthermore, the synaptic GluR1-AMPA recruitment was mediated by CREB and independent of PI3K/Akt (Ohtake et al., 2014). In this context, BDNF co-induces GluR1 synthesis via the mTor pathway, modulates the synaptic AMPAR insertion in the hippocampus and improves memory formation (Li and Keifer, 2008; Slipczuk et al., 2009), while Aβ suppresses the conversion of proBDNF to BDNF to interfere with AMPAR delivery (see also Figure 1; Zheng et al., 2010).

As such, the evidence suggests that GLP-1Rs mainly act on a postsynaptic level to potentiate the hippocampal LTP induction and sustain glutamatergic neurotransmission over the long-term by promoting synaptic AMPAR insertion. GLP-1R agonists accomplish the latter by suppressing GSK-3β via the PI3K/Akt pathway and through stimulating the CREB/BDNF pathway. Additionally, by engaging the cAMP/PKA pathway, GLP-1R agonists can restore impairments in glutamate release at presynaptic terminals in the hippocampal CA1 region. GLP-1R agonists have clear protective effects on synapses in animal models of AD and PD (Hölscher, 2022a).

### Beneficial effects of GLP-1 receptor agonists on learning and memory

In agreement with lessening synaptic injury and plasticity deficits, the treatment with GLP-1R or GLP-1R/GIPR dual agonists rescued cognitive decline, including deficits in spatial learning, recall and memory consolidation in the Morris Water Maze (During et al., 2003; Wang et al., 2010, 2018, 2019; McClean et al., 2011, 2015; Chen et al., 2012; Li L. et al., 2012; Han et al., 2013; Cai et al., 2014, 2021; McClean and Holscher, 2014a; Qi et al., 2016; Chen S. et al., 2017; Palleria et al., 2017; Shi et al., 2017; Cao et al., 2018; Panagaki et al., 2018; An et al., 2019; Garabadu and Verma, 2019; Li et al., 2020a; Maskery et al., 2020; Yu et al., 2020; Park et al., 2021; Zheng et al., 2021), short-term spatial working memory in the Y-Maze (Qi et al., 2016; Cai et al., 2017, 2021; Shi et al., 2017; Cao et al., 2018; Garabadu and Verma, 2019; Li et al., 2020a; Yu et al., 2020; Park et al., 2021), hippocampus-mediated passive avoidance learning and memory retainment (During et al., 2003; Palleria et al., 2017; Park et al., 2021), cued and contextual fear learning and memory (Ma et al., 2012; Lourenco et al., 2013; Cai et al., 2021), active avoidance memory retention (Hansen et al., 2015), as well as object recognition memory (McClean et al., 2011, 2015; McClean and Holscher, 2014b; Batista et al., 2018; Cai et al., 2021) in various AD *in vivo* models and SAMP8 mice. A 4 week -long application of liraglutide further improved various cognitive measures in a small-scale pilot study that recruited adults with mood disorders (Mansur et al., 2017). On the other hand, the spatial and object memory-enhancing effects of GLP-1 or its synthetic analogs were blocked by the co-administration of GLP-1R antagonists (During et al., 2003; McGovern et al., 2012; Zhou et al., 2019), while GLP-1R^–/–^ mice exhibited deficiencies in spatial acquisition and recollection as well as associative and object discrimination memory (During et al., 2003; Abbas et al., 2009). Notably, the vast majority of studies suggest that acute injections of GLP-1R or GLP-1R/GIPR dual agonists do not adversely affect anxiety, visual function, exploratory or locomotor behavior (McClean et al., 2011, 2015; Li L. et al., 2012; McGovern et al., 2012; Han et al., 2013; McClean and Holscher, 2014b; Qi et al., 2016; Cao et al., 2018).

Besides direct effects on learning and memory, Exendin-4 or GLP-1/GIP dual agonists further restored Aβ_31–35_-triggered distortions in the hippocampal circadian rhythm, including improvements in the circadian regulatory and CREB-modulating proteins Per1/2 and the synaptic re-modeler growth associated protein 43 (GAP-43) (Wang et al., 2016, 2019). Moreover, GLP-1R activation enhanced the decreased theta band frequencies in response to the CA1 microinjection of STZ (Li et al., 2020a). Notably, theta rhythms are generated by projections of cholinergic and GABAergic neurons from the nucleus basalis toward the hippocampus, regulating the magnitude of evoked hippocampal action potentials (Wyble et al., 2000). Indeed, the disturbance of theta rhythm was shown to impair hippocampal spatial learning, memory and movement (Winson, 1978).

Due to its pivotal role in the neuronal growth, proliferation, differentiation, plasticity, neurogenesis and memory, the cAMP/PKA/CREB pathway is the most sought-after drug target for AD (Sharma and Singh, 2020). The induction of the cAMP/PKA/pCREB pathway not only mediates plasticity and long-term memory formation in the hippocampus (Bourtchuladze et al., 1994; Impey et al., 1996), but is also mandatory for neuronal survival (Ao et al., 2006). In the hippocampus, the CREB-associated expression of plasticity genes, including c-fos, activity-regulated cytoskeleton-associated protein and BDNF, drive declarative memory consolidation to convert short-term into long-term memory (Ortega-Martinez, 2015). Indeed, the genetic upregulation of CREB activity elevated long-lasting LTP and long-term memory consolidation in the hippocampal CA1 region, while the concomitant transcription of BNDF supports memory formation by CREB, while facilitating short-term memory (Suzuki et al., 2011).

Unsurprisingly, cAMP/PKA/pCREB-signaling is downregulated in AD patients in key areas such as the hippocampus (Yamamoto-Sasaki et al., 1999; Liang et al., 2007; Bartolotti et al., 2016). The latter signaling pathway is impeded by Aβ-triggered cytosolic Ca^2+^ overload and the concomitant induction of the Ca^2+^-sensitive and CREB-proteolyzing enzyme calpain. Likewise, CREB induction was shown to be responsible for glucose transporter type 3 (GLUT3) expression, negatively associated with astrogliosis and impaired by oxidative stress (Puzzo et al., 2005; Pugazhenthi et al., 2011; Jin et al., 2013).

From a mechanistic standpoint, a study demonstrated that the GLP-1 mimetic-evoked improvements in associate memory retainment post training were dependent on GLP-1R/ERK-signaling in the murine hippocampus (During et al., 2003). Also promoted by GLP-1R activation (Hölscher, 2020), it was recently shown that the activation of ERK following cAMP/Epac-signaling was required to convert short-lasting and protein synthesis-independent LTP into protein translation-dependent forms of LTP to sustain plasticity (Gelinas et al., 2008). Indeed, ERK controls cognition by navigating protein synthesis, gene expression, dendritic spine remodeling, ion channel regulation and receptor insertion, such as those of AMPARs in synapses (Sweatt, 2004). As we elaborate in the respective sections, GLP-1 analogs further maintain the activity of the memory master regulator CREB (which lies downstream of ERK) by protecting from amyloid-provoked Ca^2+^ deregulation (section “GLP-1 mimetics suppress Ca^2+^ deregulation by amyloid beta and excitotoxicity”), oxidative stress (section “”Oxidative stress and mitochondrial dysfunction”) and cerebral insulin resistance (section “Insulin resistance and the neuronal energy metabolism”). Moreover, GLP-1R activation concertedly stimulates the PI3K/Akt, ERK, and cAMP/PKA pathways to enhance CREB activity and BDNF synthesis in hippocampal neurons *in vitro* and *in vivo*, even in non-pathological conditions (see section “Other growth factors”) (Perry et al., 2002b; Velmurugan et al., 2012; Ohtake et al., 2014; Gumuslu et al., 2016; Tai et al., 2018; Park et al., 2021).

Interestingly, the s.c. injection of liraglutide or DDP-4-inhibitors (which are mostly poorly BBB-penetrant, but block the degradation of peripheral GLP-1) enhanced the expression of GLP-1Rs in the hippocampus of WT, APP/PS1 or Aβ_1–42_ i.c.v injected mice (Qi et al., 2016, 2017; Chen S. et al., 2019). Since the adenovirus-mediated overexpression of GLP-1Rs by hippocampal neurons enhanced spatial, but not associative, learning *in vivo* (During et al., 2003), it can be hypothesized that the GLP-1-mediated upregulation of GLP-1Rs might be beneficial for memory acquisition. Interestingly, GLP-1R mRNA expression was found to be increased more than 10-fold in the SN of patients with PD compared to controls after treatment with a GLP-1R agonist (Yun et al., 2018).

## Amyloid beta and Tau pathology in Alzheimer’s disease

### GLP-1 receptor agonists are neuroprotective and prevent amyloid beta accumulation *in vivo*

As stipulated by the amyloid hypotheses, the abnormal production, accumulation and aggregation of Aβ_1–40_ and Aβ_1–42_ monomers to oligomers and plaques, as a consequence of impaired Aβ degradation and clearance, has been thought to be a central pathological event in AD. Briefly, Aβ and the soluble fragment sAPPβ may be generated by the sequential cleavage of APP by BACE1 and γ-secretase (amyloidogenic pathway), whereas APP-processing by α-secretases [a disintegrin and metalloproteinase (ADAM) protein] and γ-secretase lead to the production of p3 and sAPPα (non-amyloidogenic and non-toxic pathway). As backed up by countless clinical failures, it is now clear that Aβ is a contributing, but not a leading, factor in the development of AD (Kametani and Hasegawa, 2018; Schneider, 2019; Knopman et al., 2020).

Multiple studies confirm that GLP-1 analogs prevent neuronal death and the Aβ pathology *in vivo*. GLP-1 analog treatment prevented neuronal atrophy in the hippocampus and cortex of 3 × Tg mice (Chen S. et al., 2017; Zheng et al., 2021) and 5xFAD mice (Xie et al., 2021) as well as the hippocampal CA1 region of rats following the i.c.v.-injection of STZ (Chen et al., 2012; Palleria et al., 2017). A GLP-1 analog further rescued the cerebral, renal and splenic vasculature from Aβ-induced lesions and leakage in APP/PS1 mice, indicating vasoprotective effects (Kelly et al., 2015). Moreover, various GLP-1R or GLP-1R dual agonists lowered the cerebral levels of APP in the cortex (Li et al., 2010b) or across the brain (McClean and Holscher, 2014a), reduced soluble monomeric Aβ in the prefrontal cortex (Li et al., 2010b; Garabadu and Verma, 2019), hippocampus (Garabadu and Verma, 2019) or centrally (Perry et al., 2003; McClean and Holscher, 2014a; Paladugu et al., 2021), decreased cortical (McClean et al., 2011) or hippocampal (Lourenco et al., 2013) Aβ oligomer pools and diminished the Aβ plaque load in the cortex (McClean et al., 2011, 2015; Gengler et al., 2012; McClean and Holscher, 2014b; Panagaki et al., 2018; Salles et al., 2018, 2020; Wang et al., 2018; Maskery et al., 2020), hippocampal CA1 area (Clemmensen et al., 2013; McClean et al., 2015; Cai et al., 2018, 2021; Cao et al., 2018; Panagaki et al., 2018; Salles et al., 2018, 2020; Wang et al., 2018; An et al., 2019; Maskery et al., 2020), dentate gyrus (DG) (Salles et al., 2018, 2020; Maskery et al., 2020)or globally (McClean and Holscher, 2014a) in APP/PS1, 3 × Tg-AD, 5xFAD and i.c.v. STZ-injected rodents. Interestingly, Liraglutide preserved hippocampal pyramidal neurons in SAMP8 mice, an animal model sporadic AD that does not exhibit Aβ or Tau pathologies. This suggests that the GLP-1R-meditated neuroprotection in Aβ-based animal models presumably involves Aβ-dependent and Aβ-independent neuroprotective mechanisms (Hansen et al., 2015).

As illustrated in Figure 1, GLP-1R activation suppresses the cerebral Aβ pathology by various means. First, GLP-1 analogs enhanced the cortical and hippocampal expression of insulin degrading enzyme (IDE) in WT, APP/PS1 and 5xFAD mice (McClean and Holscher, 2014a; Paladugu et al., 2021; Park et al., 2021). In this context, nephrilysin and IDE pose the main Aβ-degrading enzymes in the brain. The intraneuronal enzyme IDE preferably cleaves Aβ_1–40_ and Aβ_1–42_ monomers, but not Aβ oligomers or fibrils, implying that reductions in IDE increase the Aβ oligomer/monomer ratio (Saido and Leissring, 2012). Indeed, IDE levels and concentrations were shown to decline in the hippocampus of MCI and AD patients, inversely correlating with the amount of Aβ_1–42_ (Zhao et al., 2007). As a possible link, *in vivo* studies suggest that the development of T2DM accelerates Aβ accumulation in the brain by reducing IDE and enhancing γ-secretase activity (Ho et al., 2004). Notably, as demonstrated *in vitro*, the stimulation of IR by insulin results in the PI3K/Akt-mediated upregulation of IDE as a negative feedback loop to prevent chronic IR-signaling (Zhao et al., 2004). However, insulin-signaling and the induction of the PI3K/Akt pathway are impaired during AD (Holscher, 2019). Indeed, reduced PI3K levels, as indicative of CNS insulin resistance, were observed in the brains of AD patients and in animals, correlating with lessened IDE and elevated Aβ quantities *in vivo* (Zhao et al., 2004). In turn, GLP-1R agonists recover the expression of IDE by restoring Aβ-driven impairments in insulin sensitivity and boosting PI3K/Akt-signaling in the brain (Bomfim et al., 2012; Long-Smith et al., 2013; Shi et al., 2017; Paladugu et al., 2021).

Second, it was shown that Exendin-4 encouraged the membrane trafficking and cleavage activity of the APP-shedding enzyme ADAM10, the main α-secretase, in the neocortex of WT and oligomeric Aβ-injected mice (Ohtake et al., 2014).

Third, GLP-1 mimetics interfere with the amyloidogenic turnover of APP by BACE1. The application of GLP-1 or exendin-4 decreased amyloidogenic APP processing in PC12 cells, as implied by the reduced intracellular and secreted sAPPβ levels (Perry et al., 2003). An *in vitro* study demonstrated that, by re-sensitizing the insulin pathway in neurons, liraglutide diminished the abnormally increased activity of BACE1, the key amyloidogenic secretase that generates Aβ from its precursor APP in concert with γ-secretase (Zhang and Song, 2013). This led to reduce Aβ/APP conversion ratios and Aβ plaque formation (Jantrapirom et al., 2020). Likewise, liraglutide or NLY01 attenuated BACE1 expression and APP turnover in the hippocampus of 5xFAD mice (Park et al., 2021) and blocked the neuronal upregulation of BACE1 in response to okadaic acid (OA)-triggered Tau hyperphosphorylation *in vitro* and *in vivo* (Yu et al., 2020). Notably, similar to the pro-inflammatory cytokine tumor necrosis factor alpha (TNF-α), GSK-3β is implicated in the activation of NF-κB, resulting in the increased expression of BACE1, APP cleavage, Aβ production, plaque formation, and impaired memory. In turn, the latter adverse changes could be prevented with the pharmacological inhibition of GSK-3β in APP23/PS45 mice (Ly et al., 2013). In this context, GLP-1R activation was shown to promote PI3K/Akt-signaling in neurons to inactivate the BACE1-upregulating GSK-3β *in vitro* and *in vivo* (Cai et al., 2014; Qi et al., 2016; Cao et al., 2018; Wang et al., 2018; Zhou et al., 2019; Jantrapirom et al., 2020).

Fourth, as another link to amyloidogenesis, GLP-1R induction normalizes the rate of N-glycosylation. A recent study showed that GLP-1 agonists preserved β-catenin levels through the Akt-mediated inhibition of GSK-3β. As dependent on the Akt/GSK-3β/β-catenin pathway, this resulted in the normalization of the aberrantly elevated N-acetylglucosaminyltransferase III (GnT-III) activity and concomitant increase in bisecting N-acetylglucosamine (GlcNAc) levels in GLP-1 mimetic and Aβ_25–35_-co-treated neurons *in vitro* and the hippocampus and cortex of APP/PS1 mice *in vivo* (Wang et al., 2018). While the mechanism is still elusive, it was shown that the nuclear translocation of β-catenin, as maintained by Wnt-pathway agonism that disassembles the “destruction complex” (GSK-3β, axin, diversin, and polyposis coli) or the pharmacological inhibition of the GSK-3β-mediated phosphorylation of β-catenin that targets it for proteasomal degradation, attenuated GnT-III expression, Aβ accumulation, plaque formation, gliosis and spatial memory deficits in APP/PS1 mice (Toledo and Inestrosa, 2010; Xu et al., 2011; Rios et al., 2014). In the context of AD, amyloidogenic proteins, including BACE1 (Kizuka et al., 2015), APP (Akasaka-Manya et al., 2008), and Tau (Sato et al., 2001a), were shown to be N-glycosylated by GnT-III. Interestingly, the GlcNAc-modification of BACE1, as stimulated by Aβ-triggered oxidative stress, hampered its lysosomal targeting and degradation (Kizuka et al., 2015, 2016). In addition, elevated GnT-III activity was shown to impair growth factor signaling, such as the blockade of nerve growth factor (NGF) receptor dimerization and (Ihara et al., 1997) epidermal growth factor (EGF) receptor phosphorylation (Rebbaa et al., 1997) or aberrantly increased EGF receptor internalization, which seemingly upregulates the induction of ERK (Sato et al., 2001b). Given that AD patients displayed enhanced hippocampal GSK-3β activity, decreased β-catenin levels, heightened GnT-III expression and increased bisecting GlcNAc pools, treatment of Wnt pathway dysfunction, which might be achieved with GLP-1R activation, has been proposed as a therapeutic strategy for AD (Akasaka-Manya et al., 2010; Rios et al., 2014; Palmigiano et al., 2016).

Fifth, GLP-1 agonists block the induction of c-Jun N-terminal kinase (JNK) to prevent the generation of Aβ. In AD, JNK is induced by oxidative stress derived from various sources, for example in response to the Aβ or Tau pathology, neuroinflammation or mitochondrial dysfunction. Moreover, glial inflammation, as provoked by oligomeric Aβ and plaques, leads to the release of pro-inflammatory cytokines [i.e., interleukin (IL)-1β, IL-18, and TNF-α] that activate the corresponding pro-inflammatory cytokine receptors (PICRs) on neurons. Downstream inflammatory signaling by various modulators results in the mitogen-activated protein kinase kinase (MKK)-driven phosphorylation of p38 and JNK. Ultimately, JNK/p38 co-induce the activator protein 1 (AP-1)-mediated transcriptional upregulation of APP, while PICR stimulation separately provokes the NF-κB-driven expression of BACE1 in neurons (Ojala and Sutinen, 2017; Kheiri et al., 2018). As an insulin receptor substrate 1 (IRS-1)-inactivating serine kinase, JNK, in cooperation with the inflammation/PICR-induced serine kinases inhibitor of κB–kinase β (IKKβ) and protein kinase R (PKR), also induces neuronal insulin resistance to exacerbate the production, accumulation and aggregation of Aβ (see Figure 1 and details in section “Insulin resistance and the neuronal energy metabolism”) as well as Tau hyperphosphorylation (section “GLP-1R mimetics suppress Tau hyperphosphorylation and aggregation during AD”). Lastly, Aβ_1–42_-driven endoplasmic reticulum (ER) stress is linked to the activation of JNK3, which was shown to augment APP phosphorylation by JNK3 at Thr^668^ to encourage amyloidogenic processing (Yoon et al., 2012). Indeed, Aβ was shown to induce the JNK/TNF-α pathway in neurons to elicit the inactivation of IRS-1, CNS insulin resistance and memory decline (Ma et al., 2009; Bomfim et al., 2012), while Exendin-4 suppressed JNK, blocked the cerebral desensitization of insulin-signaling and restored spatial memory in APP/PS1 mice (Bomfim et al., 2012). Likewise, Liraglutide normalized the decreased ERK activity and the elevated levels of phosphorylated JNK_1/2_ in the brains of 3 × Tg triple transgenic mice (Chen S. et al., 2017). A study in rotenone-based PD models suggests that GLP-1R activation reduces oxidative stress in an Akt-dependent manner to prevent the detrimental activation of JNK (Li et al., 2020c). Moreover, as elaborated in section “Inflammation,” GLP-1 analogs resolve glial inflammation and cytokine production, which boost JNK activation via PICRs in neurons (Holscher, 2019), in various contexts.

### GLP-1 receptor mimetics suppress Tau hyperphosphorylation and aggregation during Alzheimer’s disease

It has been proposed that differences in the expression of insulin genes explain the vulnerability of distinct brain regions to the Aβ and Tau pathology, as exacerbated though the development of CNS insulin resistance during AD (Mullins et al., 2017). For example, cell culture studies have demonstrated that insulin resistance in neurons creates an AD-like phenotype that exhibits attenuated IR/IRS-1/PI3K/Akt-signaling, enhanced activity of the Tau kinase GSK-3β activity due to the loss of Akt-signaling, deregulated ERK_1/2_, acetylcholinesterase and pro-inflammatory nuclear factor kappa B (NF-κB) activities, impaired plasma membrane recruitment of GLUT4 by insulin, the reduced expression of insulin-independent GLUT3, diminished glucose uptake, elevated BACE1 activity, increased Aβ production, secretion and plaque formation as well as, of course, heightened Tau hyperphosphorylation and aggregation (Gupta et al., 2011; Jantrapirom et al., 2020). Notably, T2DM animal models show elevated Tau phosphorylation and inactivation of the insulin pathway (IR and Ser-IRS-1 phosphorylation) in the brain (summarized in Mullins et al., 2017), while phospho-Ser-inhibited IRS-1 co-localized with neurofibrillary tangles (NFTs) in pyramidal neurons in the brains of AD patients (Moloney et al., 2010).

As repeatedly shown, GLP-1R and GLP-1R/GIPR dual agonists reversed Tau hyperphosphorylation *in vivo*. This involved a decrease in the cortical or hippocampal Thr^181^ (Wang et al., 2018), Thr^181^/Ser^396^ (Chen et al., 2012), Thr^231^ (Chen S. et al., 2017; Holubova et al., 2019), Ser^199/202^/Ser^396^ (Qi et al., 2016), Ser^199/202^/Ser^404^ (Zhou et al., 2019), Ser^202^/Thr^205^ (Li L. et al., 2012; Cao et al., 2018), Thr^212^/Ser^214^ (Hansen et al., 2015), Ser^396^ (Shi et al., 2017; Li et al., 2020a), Ser^199/202^/Ser^396^ (Yu et al., 2020), and Ser^199/202^/Ser^214^/Ser^396^-phosphorylated Tau levels (Chen S. et al., 2017) as well as hyperphosphorylated Tau neurofilament pools, as accumulating in the cell bodies and neurites of neurons (Chen S. et al., 2017; Zhou et al., 2019), in APP/PS1 mice, 3 × Tg mice, Aβ_1–42_-microinjected rodents, i.c.v. STZ-injected rats, a 3 × Tg AD/T2DM crossover animal model, OA-microinjected rodents or hTauP301L mice. Liraglutide further diminished the numbers of neurons containing Ser^202^ and Ser^212/214^-phosphorylated Tau as well as oligomeric Tau and fragmented Tau in the DG and amygdala of primates (Batista et al., 2018). Moreover, GLP-1R induction reduced the numbers of Tyr^231^- pTau-positive neurons and the formation of Tau inclusions in the hippocampal tissue of Tau APP/PS1 and 3 × Tg mice (Shi et al., 2017; Cai et al., 2018, 2021; Cao et al., 2018; Wang et al., 2018). Notably, the injection of GLP-1 mimetics lowered the elevated total Tau levels in some AD animal studies (Li L. et al., 2012), but had no effect on soluble Tau in others (Li et al., 2010b; Chen et al., 2012). In support of their Tau pathology-ameliorating properties, liraglutide reduced clasping behavior and massively extended the survival of hTauP301L mice, whereby the GLP-1 analog-mediated reduction in phospho-Tau levels correlated with life span improvements (Hansen et al., 2015).

Mechanistically, the latter AD-like rodents displayed reduced PI3K and Akt, but enhanced activity of the Tau kinase GSK-3β in the hippocampus, as indicative of desensitized insulin signaling. On the other hand, the application of incretin analogs re-induced the PI3K/Akt pathway in a GLP-1R-mediated manner and increased the expression of GLP-1 and GLP-1R. This led to the Akt-driven Ser^9^-phosphorylation and inactivation of GSK-3β, while the levels of activated (Tyr^216^-phosphorylated) GSK-3β, as stimulated by Aβ, were reduced (Cai et al., 2014; Qi et al., 2016; Cao et al., 2018; Wang et al., 2018; Zhou et al., 2019). Indeed, liraglutide re-invigorated the insulin-evoked phosphorylation and activation of IRs, IRS-1, Akt as well as the inhibitory Ser^9^-phosphorylation of GSK-3β by Akt in insulin-resistant SH-SY5Y cells. The GLP-1-elicited normalization of insulin-signaling resulted in lessened Ser^396^ phospho-Tau pools, albeit it did not normalize the cellular glucose uptake (Jantrapirom et al., 2020). Notably, the effects of GLP-1 mimetics in AD models are mixed, which might be related to the treatment protocol. For example, GLP-1_9–36_*^amide^* and Exendin-4 enhanced the hippocampal Akt and reduced GSK-3β activity in APP/PS1 mice (Ma et al., 2012; Wang et al., 2018), while liraglutide elevated the inactivated phopsho-Ser^9^-GSK-3β levels in the cortex or hippocampus of WT, 3 × Tg and 5xFAD animals (Paladugu et al., 2021). However, liraglutide failed to affect the induction of GSK-3β in other studies (McClean and Holscher, 2014a; Holubova et al., 2019). Also, while Exendin-4 normalized the hippocampal expression levels of total GSK-3β in the i.c.v. STZ-injected rat AD/T2DM model (Chen et al., 2012), liraglutide did not reduce the elevated GSK-3β pools in Aβ-based AD animals (McClean and Holscher, 2014a; Qi et al., 2016; Holubova et al., 2019). Notably, GLP-1 mimetics do not affect the cerebral Tau protein phosphatase 2A (PP2A) levels *in vitro* or *in vivo* (An et al., 2015; Holubova et al., 2019). However, the administration of liraglutide prevented neuronal apoptosis and cognitive deficits in response to the PP2A-inhibitor OA *in vitro* and *in vivo* (Yu et al., 2020). Synoptically, the presented evidence suggests that GLP-1 agonists do not affect Tau phosphatase activity and predominantly suppress Tau hyperphosphorylation by enhancing insulin-sensitivity and the Akt-driven inhibition of GSK-3β during AD (Figure 1).

Another indirect mechanism that prevents Tau hyperphosphorylation is the suppression of the p38 and JNK pathways as well as neuroinflammation by GLP-1R agonists (pathways depicted in Figure 1). It was discovered that increased JNK and p38 activities co-localize with NFT-bearing, but not apoptotic, neurons in tissue samples of patients with various Tauopathies, including AD (Atzori et al., 2001). For example, the pro-inflammatory cytokine IL-1β, as released by Aβ or lipopolysaccharide (LPS)-stimulated microglia, induces the activation of p38 to drive Tau phosphorylation and synapse loss (Sheng et al., 2001; Li et al., 2003). Similarly, IL-18 elevated the expression of the Tau serine/threonine kinases GSK-3β, cyclin-dependent kinase 5 (Cdk5) as well as its regulatory subunit p35 (Ojala et al., 2008). Notably, along with the Tau phosphorylation at Thr^212^ and Ser^214^ by PKA, p38δ also appears to phosphorylate Tau at Thr^217^ to create the AT100 epitope that is commonly detected with Tau antibodies (Yoshida and Goedert, 2006). Of note, p35 may be truncated to produce the more proteolytically resistant p25, which seems to accumulate in the brains of AD patients and constitutively activates Cdk5 (Patrick et al., 1999; Tseng et al., 2002). It is thought that the Ca^2+^-sensitive calpain, as induced by Aβ-triggered Ca^2+^-overload, cleaves p35 into p25 (Lee et al., 2000). Other negative events include the calpain-mediated cleavage of GSK-3β to increase its activity, Tau fragmentation [which may be neurotoxic and/or impair oxidative phosphorylation (OXPHOS)], the joint downregulation of dynamin 1, a presynaptic vesicle recycler, with Aβ, the proteolysis of synaptic proteins [i.e., PSD-95, NMDAR subunits NR1/2A/2B, metabotropic glutamate receptor subtype 1 (mGluR1)] and cleavage of PKA to interrupt CREB activity (Ferreira, 2012). On the other hand, studies in various disease models indicate that JNK is selectively stimulated by Aβ as well as proinflammatory cytokine exposure, but not Tau, in neurons *in vivo* and post-mortem hippocampal brain tissue of AD patients (Ma et al., 2009; Bomfim et al., 2012; Killick et al., 2014; Ojala and Sutinen, 2017). The JNK isoforms (JNK1/2/3) act as Tau kinases, phosphorylating Tau at Ser^199^, Thr^212^ and Ser^422^ to accelerate Tau aggregation and microtubule depolarisation (Zhao et al., 2022). Importantly, inflammatory cytokine/PICR-signaling in neurons and the concomitant activation of the IRS-1-inactivating serine kinases JNK, IKKβ, and PKR lead to cerebral insulin resistance in AD which, in turn, promotes Tau hyperphosphorylation by enhancing the activity of the Tau kinase GSK-3β (Holscher, 2019; Akhtar and Sah, 2020). JNK further drives apoptosis by phosphorylating and activating the apoptosis-drivers BIM [a Bax and Bak activator and B-cell lymphoma 2 (Bcl-2)/myeloid cell leukemia 1 (Mcl-1) inhibitor (Chi et al., 2020)] and Bcl-2-modifying factor (BMF) as well as suppressing the activity of their anti-apoptotic counterparts, i.e., Bcl-2 (Zhao et al., 2022). Aberrant JNK activation, due to the phosphorylation of PSD-95, also disrupts the recruitment of AMARs and NMDARs, triggers synapse (PSD-95 and drebrin) and spine degeneration and induces LTP impairments (Sclip et al., 2014; Zhao et al., 2022). In the aforementioned context, GLP-1 analogs were shown to inhibit the Tau pathology-associated activities of p38 in the hippocampus of APP/PS1 mice (Cai et al., 2018) and JNK in multiple Aβ-induced AD animal models (Bomfim et al., 2012; Chen S. et al., 2017). Additionally, GLP-1R activation on glial cells is immunosuppressive (section “Inflammation”), thus forestalling the cytokine/PICR-induced activation of p38 and JNK, the upregulation of GSK-3β, Cdk5 and p35 as well as, of course, the desensitization of insulin-signaling in the brain (as expanded on in section “Insulin resistance and the neuronal energy metabolism”).

## GLP-1 mimetics suppress Ca^2+^ deregulation by amyloid beta and excitotoxicity

Amongst other toxic implications, soluble oligomeric Aβ provokes neuronal death by stimulating Ca^2+^-overload in AD. Aβ may drive Ca^2+^ accumulation by forming pores in the plasma membrane. Furthermore, Aβ triggers reactive oxygen species (ROS) production due to the reaction with iron/copper ions or by impairing the mitochondrial electron transport chain (ETC), leading to plasma membrane lipid peroxidation, the subsequent inhibition of Ca^2+^ ATPases, membrane depolarisation and Aβ/ROS-induced NMDAR and L-type VDCC opening and excessive Ca^2+^-influx (Ueda et al., 1997; Fu et al., 2006; Zundorf and Reiser, 2011). The Aβ-induced Ca^2+^-influx across NMDARs seems to evoke a compensatory transcriptional downregulation of ryanodine receptor (RyR) subunits in the ER membrane, while, simultaneously, stimulating ER Ca^2+^ efflux into the cytosol across RyRs and inositol 1,4,5-trisphosphate (IP3R) receptors. This resulted in oxidative stress, mitochondrial fragmentation, mitochondrial membrane permeabilization and intrinsic apoptosis *in vitro* (Ferreiro et al., 2008; Paula-Lima et al., 2011). Indeed, a recent study discovered that AD patients displayed upregulated efflux and downregulated influx Ca^2+^ transporter gene expression in mitochondria, suggesting that these are adaptive responses to maintain the mitochondrial function in response to the pathological cytosolic Ca^2+^ amassment in AD (Calvo-Rodriguez et al., 2020). Notably, Aβ-induced Ca^2+^ overload impairs the synaptic function by inducing calpain and the degradation of dynamin 1, which is implicated in synaptic vesicle recycling (Kelly and Ferreira, 2006).

*In vitro* studies confirm that GLP-1 treatment prevents pathological Ca^2+^ overload in neurons. GLP-1 and Exendin-4 dose-dependently rescued from Aβ_25–35_ or Aβ_1–42_-evoked apoptosis in primary hippocampal neurons and SH-SY5Y cells (Perry et al., 2003; Qin et al., 2008; Li et al., 2010b; Cai et al., 2017). The latter neuroprotection involved GLP-1R-induced cAMP accumulation, Akt and MEK_1/2_ activation. Importantly, Aβ suppressed the activation of the Akt/MEK_1/2_-signaling pathway and triggered Ca^2+^ release from the ER stores, whereas GLP-1R activation, as dependent on MEK_1/2_, prevented Ca^2+^ deregulation. Moreover, GLP-1 blocked the transcriptional increase in p53 and Bax that was provoked by Aβ (Qin et al., 2008; Cai et al., 2017). In this context, the pro-apoptotic protein Bax is upregulated upon cell stress by p53-mediated gene expression (Antonsson, 2001), while the p53/Bax pathway is downregulated in response to the activation of Akt (Rai et al., 2019). Besides shielding against Aβ-mediated ER Ca^2+^ efflux, GLP-1 prevented excitotoxic death by blocking glutamate-elicited Ca^2+^ influx through VDCCs and membrane depolarization in cultured hippocampal neurons (Gilman et al., 2003). Notably, while GLP-1R activation maintains the neuronal Ca^2+^ homeostasis and prevents excessive L-VDCC activity and Ca^2+^ instream following K^+^-ion overload, Aβ or glutamate-associated stress (Gilman et al., 2003; Qin et al., 2008; Cai et al., 2017), GLP-1-mediated cAMP/PKA-signaling evokes a rapid, but transient (2 min), elevation of intracellular Ca^2+^ through the opening of L-type VDCCs and ionotropic glutamate receptor channels (AMPA, NMDA, and kainite receptors) in hippocampal neurons (Kavalali et al., 1997; Gilman et al., 2003; Hölscher, 2020). The short-term Ca^2+^ accumulation that is triggered by GLP-1 may evoke the spontaneous presynaptic release of glutamate in the hippocampus (see section “GLP-1 enhances hippocampal synaptic plasticity”).

**FIGURE 2:**
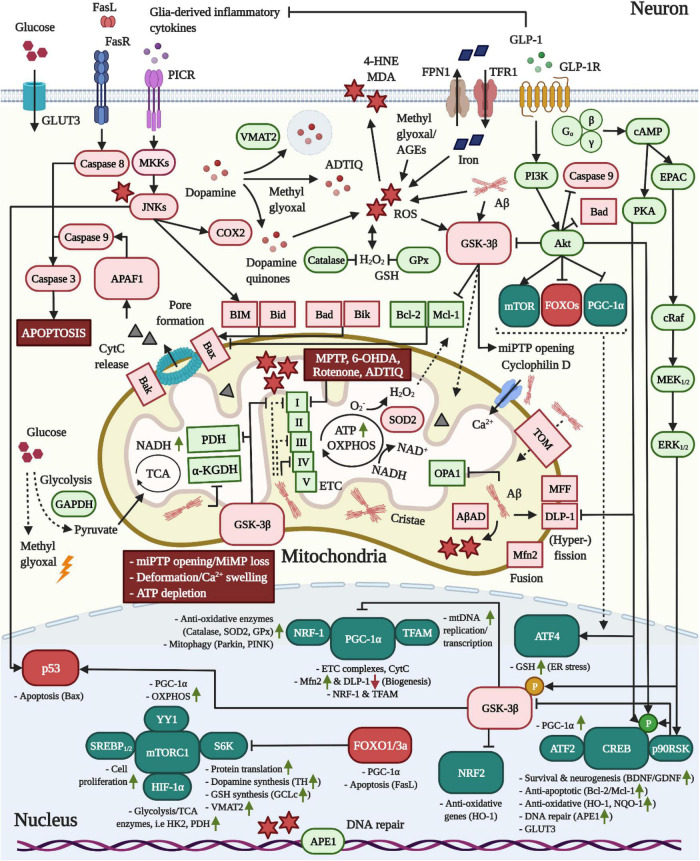
Pro-mitochondrial, anti-oxidative and anti-apoptotic effects of GLP-1 in neurons. **1** In AD, Aβ is translocated into mitochondria via TOM and accumulates in cristae, leading to elevated ROS production through the interaction with AβAD and the impairment of the TCA enzymes PDH/α-KGDH as well as complex VI, but also I and II, of the ETC. Moreover, Aβ triggers mitochondrial Ca^2+^ instream and swelling by binding to cyclophilin D and stimulates mitochondrial fragmentation by altering the expression of fusion/fission-modulating proteins. **2** As common for both neurodegenerative diseases, pro-inflammatory cytokine signaling across PICRs stimulates JNK to activate BIM and Bax-expression via p53. While dopamine is packaged into synaptic vesicles by VMAT2 in dopaminergic neurons, JNK may induce COX2 to encourage the production of reactive dopamine quinones. Pathologic alterations in the expression and localization of GAPDH as well as insulin resistance-associated impairments in the expression of glycolytic enzymes may accelerate the build-up of the AGE and ROS-generating compound methyl glyoxal. The latter was shown to react with dopamine to create ADTIQ, which amasses in nigrostriatal brain areas and, similar to the PD-toxins MPTP, 6-OHDA or rotenone, inhibits complex I of the ETC to stimulate ROS production in neurons. Metal ion accumulation in the brain, in particular the iron-mediated ROS production, lipid peroxidation, mitochondrial dysfunction and ferroptosis are implicated in AD and PD. **3** Crucially, as apparent in AD, Aβ and ROS activate GSK-3β, which promotes the trafficking of GSK-3β into mitochondria to induce the opening of miPTPs, interfere with ATP production/OXPHOS by inhibiting PDH and ETC complexes and drive apoptosis by stimulating the p53-mediated synthesis of Bax and inactivating the anti-apoptotic Mcl-1. GSK-3β further suppresses NRF2-driven anti-oxidative gene transcription and elicits the degradation of PGC-1α via SCF-Cdc4 E3 ligase. **4** Metabolic stress following TCA/OXPHOS/ETC impairments and enhanced ROS load ultimately trigger miPTP opening/Ca^2+^ deregulation, deformation, MMP loss, ATP depletion and Bax/Bak-mediated pore formation in mitochondria, resulting in APAF1/Caspase 9/Caspase 3-mediated apoptosis. **5** The induction of the GLP-1R prevents all of the pathological alterations in neurons described above. First, the activation of the survival modulator Akt leads to the direct inactivation of GSK-3β, caspase 3, Bad and FOXOs. The Akt-induced stimulation of mTOR/mTORC1, in conjunction with various other transcription factors, augments the global protein translation, including that of the dopamine-synthesizing TH and VMAT2 in dopaminergic neurons, the GSH-producing GCLc, the mitochondrial biogenesis and fusion/fission-navigating PGC-1α as well as glycolytic/TCA enzyme expression. Notably, Akt further phosphorylates HKII to recruit it to the outer mitochondrial membrane to prevent miPTP opening, whereas GSK-3β induces the liberation of HKII, evoking the opposite result (not shown) (Rasola et al., 2010). Second, cAMP/PKA-signaling inhibits DLP-1, thus suppressing mitochondrial fragmentation. Third, PI3K/Akt, cAMP/PKA, and MEK/ERK-signaling lead to the induction of CREB to improve BDNF/GDNF expression (chapter “Other growth factors”), elevate the expression of anti-apoptotic Bcl-2/Mcl-1, upregulate anti-oxidative defense genes, and encourage deoxyribonucleic acid (DNA) repair via APE1. Fourth, GLP-1 blocks pro-inflammatory cytokine production by glial cells (chapter “Inflammation”) and, hence, PICR/JNK-signaling in neurons. Given the pro-mitochondrial and dopamine-enhancing effects, animal and clinical studies support the benefits of GLP-1 treatment in PD (see chapter “GLP-1 mimetics rescue nigrostriatal dopamine neuron death and dopamine depletion in PD”). For the anti-ferroptosis-associated effects of GLP-1 in AD and PD, see section GLP-1 analogs protect from iron and dopamine-induced oxidative stress and ferroptosis.

The above studies suggest that the neuroprotective effects of GLP-1 against Ca^2+^ overload are mediated by CREB. Previous studies have shown that CREB induction in response to metabolic stress and increased Ca^2+^ influx across NMDA receptors mediates survival under hypoxic and excitotoxic conditions by heightening the expression of activity-regulated inhibitor of death survival genes, such as *bdnf*, *atf3*, *btg2*, *gadd45β/γ* or *Bcl-2*. Indeed, the overexpression of CREB improves the neuronal tolerance of apoptotic stimuli, while non-functional CREB accelerates death. Notably, CREB is inactivated and degraded in hippocampal neurons following NMDA treatment, suggesting that glutamate overload drives apoptosis by impairing CREB activity (Mabuchi et al., 2001; Tan et al., 2012). Likewise, oxidative stress (H_2_O_2_) impairs CREB induction by growth factors and dose-dependently downregulates CREB expression, thus enhancing the neuronal vulnerability to apoptosis (Zhang and Jope, 1999; Fu et al., 2019). Moreover, Tau accumulation appears to interfere with CREB and drives synapse and memory impairments by stimulating the activity of the Ca^2+^-induced phosphatase calcineurin that dephosphorylates both CREB and calcium/calmodulin-dependent protein kinase IV (Yin et al., 2016). Given that MEK_1/2_ inhibitors suppressed the neuroprotective effects of lixisenatide against Aβ (Cai et al., 2017), it is implied that GLP-1 agonists achieve neuronal survival through the cAMP/exchange protein activated by cAMP (EPAC)/Raf/MEK_1/2_ and PI3K/Akt/Raf/MEK_1/2_ pathways. MEK_1/2_ subsequently induces ERK_1/2_, the latter activates p90RSK (also known as MAPKAP-K1) and p90RSK activates CREB through phosphorylation at Ser^133^. cAMP/PKA-signaling following GLP-1R induction is further involved in neuroprotection, with PKA regulating L-type VDCC activity and inducing the Ser^133^-phosphorylation of CREB (see also Figures 1, 2; Frodin and Gammeltoft, 1999; Walton and Dragunow, 2000; Murphy et al., 2014; Hölscher, 2020). As a side note, while GLP-1 agonists stimulate short-term (2 min) Ca^2+^ influx via the opening of L-type VDCCs (Gilman et al., 2003), studies in β-cells indicate that the local activation of L-type VDCCs, but not ER Ca^2+^ efflux or changes in the intracellular Ca^2+^ levels *per se*, are necessary for GLP-1R ligands to sustain ERK activation (Gilman et al., 2003; Selway et al., 2012). Lastly, given that the amyloid intracellular C-terminal domain that is produced following APP/Aβ-cleavage interferes with Ca^2+^ gene transcription (Zundorf and Reiser, 2011), the reduction in APP expression and processing by GLP-1 mimetics (Perry et al., 2003; Li et al., 2010b; McClean and Holscher, 2014a) might contribute to the maintenance of the Ca^2+^ homeostasis in neurons.

Therefore, GLP-1 agonists protect from excessive Ca^2+^ accumulation and mitochondrial dysfunction by preventing the Aβ-driven Ca^2+^ release from the ER as well as aberrant Ca^2+^ influx across NMDARs/VDCCs in response to glutamate overload. Moreover, GLP-1R activation stimulates various key modulators (PI3K/Akt, cAMP/PKA and MEK_1/2_/ERK_1/2_/p90RSK) that jointly induce CREB-mediated survival gene expression.

## GLP-1 analogs counteract endoplasmic reticulum stress

ER stress is a common pathological feature in most neurodegenerative diseases, including AD and PD. In the latter diseases, the accumulation of abnormally folded proteins, including protease-resisting Aβ, Tau and α-synuclein oligomers, may exceed the buffering capacity of cytosolic chaperones and the proteasomal degradation system to exacerbate amyloid amassment and trigger ER stress. To prevent damage, ER stress initiates the unfolded protein response (UPR), leading to the transcriptional upregulation of protein-re-folding ER chaperones and the general repression of protein translation. However, while initially protective, chronic UPR activation results in proteasomal dysfunction, oxidative stress, intracellular Ca^2+^ overload, mitochondrial damage and, ultimately, apoptosis (Lindholm et al., 2006; Ogen-Shtern et al., 2016).

ER stress and the UPR involve the activation of three major pathways that both mediate cell survival by inducing autophagy, but also trigger apoptosis under prolonged cells stress. First, the PERK/eIF2a/activating transcription factor 4 (ATF4) pathway results in eukaryotic translation initiation factor 2a (eIF2a) phosphorylation, inhibition of eIF2a-dependent protein translation as well as the ATF4-conveyed expression of autophagy genes, but also the transcription of pro-apoptotic agents, such as CAAT/enhancer-binding protein (C/EBP) homologous protein (CHOP). Second, the accumulation of unfolded proteins is detected by the ER sensor binding immunoglobulin protein (BiP, also known as GRP78), resulting in inositol-requiring enzyme 1α (IRE1α)-associated signaling cascades. This leads to the autophagy-enabling and IRE1α-elicited degradation of XBP-1, the IRE1α-mediated induction of apoptosis signal-regulating kinase 1/JNK-signaling and the JNK-mediated dissociation of Bcl-2/Beclin-1 complexes to initiate autophagy. On the other hand, IRE1α may enhance pro-apoptotic stimuli, for example by inducing bcl-2 interacting mediator of cell death (BIM) and upregulating CHOP levels. Third, the activation of ATF6 enhances XBP-1-associated autophagy, but also induces CHOP. As a key driver of UPR-related cell death, CHOP evokes (i) the decreased transcription of anti-apoptotic Bcl-2, which is coupled to enhanced ROS-production by mitochondria and concomitant glutathione (GSH) depletion; (ii) the direct enhancement of pro-apoptotic BIM and PUMA synthesis, which stimulates Bax/Bak to induce pore formation and pro-apoptotic mitochondrial cytochrome c (CytC) release; (iii) the upregulation of the Akt-inhibitor tribbles-related protein 3 to enable forkhead box protein (FOXO)3a-driven PUMA induction; (iV) the increased expression of death receptor 5 and, thus, caspase 8-mediated apoptosis and (V) the activation of oxidoreductase 1α (Ero1α) to drive the IP3R-mediated liberation of ER Ca^2+^ stores, cytosolic Ca^2+^ amassment and the induction of the Ca^2+^-sensitive calpain/caspase 12 apoptosis pathway. Notably, Aβ-triggered cytosolic Ca^2+^ accumulation, as also induced by oxidative stress-induced mitochondrial injury, result in the Ca^2+^-induced swelling of mitochondria, membrane potential collapse, mitochondrial dysfunction and exacerbated ROS-production. Mitochondrial dysfunction, in turn, stimulates CHOP expression and apoptosis via the mitochondrial pathway [Bax/Bak-driven membrane-permeabilization, CytC release, apoptotic protease-activating factor-1 (APAF-1) and caspase 3 activation, as depicted in Figure 2; Youle and Strasser, 2008; Liu et al., 2013; Song et al., 2017]. Moreover, the chronically enhanced GSK-3β activity that is found in neurons during AD facilitates ER stress by upregulating CHOP synthesis, whereas the inhibition of GSK-3β was shown to prevent ER stress-induced apoptosis (Brewster et al., 2006; Hooper et al., 2008; Meares et al., 2011).

Notably, the UPR response and expression of ER chaperons, for instance BiP and phospho-eIF2α, were shown to be elevated in APP/Aβ-based cell and animal models as well as the temporal cortex and hippocampus of AD patients. As an unfolded protein sensor, BiP initially has a useful function and was reported to detect Aβ_1–42_ and interact with APP to suppress the generation and secretion of Aβ by β/γ-secretase (Yang et al., 1998; Yoo et al., 2001; Kakimura et al., 2002; Hoozemans et al., 2005, 2009; Hoshino et al., 2007). On the other hand, phospho-eIF2α was demonstrated to aggravate BACE1 expression, Aβ generation and plaque formation in neurons. Increased phospho-eIF2α levels were also found in the brains of AD patients, seemingly triggered by insulin resistance-associated impairments in the neuronal ATP production and glucose metabolism. The increase in phospho-eIF2α pools further correlated with elevated BACE1 and plaque levels *in vivo* and in post-mortem brain tissue of AD patients (O’Connor et al., 2008).

Animal experiments have confirmed that GLP-1R activation guards against amyloid-triggered ER stress and apoptosis. In the APP/PS1 animal model, the injection of a GLP-1R/GIPR dual agonist restored the reduced cerebral Akt and (inactivated) Ser^9^-phospho-GSK-3β levels to the levels of control mice, without affecting ERK_1/2_ (Panagaki et al., 2018). Indeed, GLP-1R agonists are potent stimulators of the neuroprotective modulator Akt that directly suppresses the abnormally increased GSK-3β activity in the brain of AD animal models (Cai et al., 2014; Qi et al., 2016; Cao et al., 2018; Wang et al., 2018; Zhou et al., 2019; Jantrapirom et al., 2020). Moreover, the synthetic dual incretin supressed the Aβ-triggered upregulation of the unfolded protein-sensor BiP, CHOP as well as caspase 12 in APP/PS1 mice, implying that ER stress was resolved (Panagaki et al., 2018). Liraglutide further downregulated the levels of BiP and phospho-eIF2α in the hippocampus of APP/PS1 mice (Lourenco et al., 2013).

GLP-1 further prevents Ca^2+^-associated ER stress. As we describe in section “GLP-1 mimetics suppress Ca2+ deregulation by amyloid beta and excitotoxicity,” GLP-1 protects from Aβ or excitotoxicity-driven aberrant Ca^2+^ efflux from ER stores and Ca^2+^ instream though NMDARs/L-VDCCs. Cellular experiments with thapsigargin, which triggers excessive ER Ca^2+^-release, give further insight into the signaling mechanisms linked to ER stress. Following thapsigargin treatment, liraglutide suppressed the aberrantly upregulated expression of the ER stress indicator BiP [which lies upstream of IRE1α, PERK, and ATF6 (Lee, 2005)], activating transcription factor 6 and CHOP, while restoring the impaired activation of IRE1α and ATF6 in SH-SY5Y cells (Panagaki et al., 2017). Mechanistically, liraglutide induced Akt, normalized the lowered (Akt-induced) inhibitory phosphorylation of GSK-3β and pro-apoptotic Bcl-2-associated death promoter (Bad), recovered the reduced anti-apoptotic Bcl-2, p53 and signal transducer and activator of transcription (STAT)3 activities and diminished the basal expression of the apoptosis-eliciting protein BH3 interacting-domain death agonist (Bid). The impeded phosphorylation of ERK_1/2_ could not be restored, however (Panagaki et al., 2017). The GLP-1 mimetic further normalized the enhanced protein levels of the protein quality-control chaperones Ero1α and lessened calnexin pools (Panagaki et al., 2017), which indicates that the Ero1α/IP3R-driven, aberrant Ca^2+^ secretion from the ER and calnexin deprivation-associated protein misfolding and proteasome overload were ameliorated (Liu et al., 2013; Song et al., 2017). The excessive activity of the stress survival transcription factor NF-E2-related factor (NRF-2), which is provoked by ER and oxidative stress, the amassment of misfolded or aggregated proteins or mitochondrial ROS generation (He et al., 2020), was also quenched by liraglutide (Panagaki et al., 2017). As such, liraglutide rescued cell death, improved the cellular proliferation, blocked the apoptosis-inducer caspase 12 and the subsequent degradation of poly ADP ribose polymerase in thapsigargin-assaulted neuroblastoma cells (Panagaki et al., 2017).

Notably, despite the fact that GLP-1R activation suppresses ER stress, Exendin-4 enhanced the expression of the ER stress-associated transcription factor ATF4 to protect H_2_O_2_-subjected neuroblastoma cells from death (Li et al., 2010c). In this context, the PERK/eIF2a/ATF4 axis is required for the adaption and resistance of cells toward oxidative stress through the ATF-4 mediated transcription of autophagy and redox genes, such as GSH. However, ATF4 also controls the synthesis of pro-apoptotic factors, including CHOP, to initiate cell death upon persistent ER stress (Harding et al., 2003; Song et al., 2017). Studies in pancreatic β-cells give further insight. As expected, Exendin-4 enhanced the β-cell survival by resolving ER stress in obese mice and isolated rat β cells. Intriguingly, while the GLP-1R agonist did not affect PERK, the incretin analog further potentiated the expression of ATF4/CHOP, yet diminished the inhibitory phosphorylation of the translation repressor eIF2a upstream of ATF4, in thapsigargin or tunicamycin-treated INS-1 and MIN6 cells. GLP-1 further upregulated the XBP-1-controlled expression of the chaperone DnaJ Heat Shock Protein Family (DnaJb9), interestingly without affecting the levels or induction of the ER stress mediator XBP-1, and augmented growth arrest and DNA damage-inducible protein (GADD34) levels following ER stress. The GLP-1R-evoked increase in ATF4/CHOP levels and the dephosphorylation of eIF2a were cAMP/PKA-dependent (Yusta et al., 2006). Notably, PKA phosphorylates both human inhibitor 1 (I-1) and type 1 protein serine/threonine phosphatase (PP1), leading to the association of I-1 and PP1 with GADD34, the nuclear translocation of the latter protein complex and the PP1-mediated dephosphorylation of eIF2a to re-evoke protein translation (Connor et al., 2001). Furthermore, PKA mediates the direct phosphorylation of ATF-4 (Elefteriou et al., 2005) and the survival transcription factor CREB (Hölscher, 2020).

Therefore, GLP-1R agonists modulate the cellular fate in favor of survival during ER stress. While GLP-1R activation resolves ER stress, blocks apoptosis and re-elicits protein synthesis via the cAMP/PKA/GADD34-PP1/eIF2a axis, GLP-1R-mediated PKA-signaling seems to selectively potentiate redox gene expression via the ER stress-associated transcription factor ATF4. That aside, GLP-1R agonists stimulate chaperone expression (calnexin and DnaJb9) following Ca^2+^ deregulation (Yusta et al., 2006; Panagaki et al., 2017), which likely contributes to protein folding and forestalls proteasome dysfunction. The latter chaperone upregulation might be related to general increases in protein translation through the PI3K/Akt/mTor pathway by incretins (Garza-Lombo et al., 2018; Hölscher, 2020).

## Inflammation

### Neuroinflammation is apparent in Alzheimer’s disease and Parkinson’s disease

In their ramified or resting state, microglia monitor the CNS for the presence of damage-associated molecular patterns (DAMPs; including neuronal, glial, endothelial, or oligodendrocyte-derived material) or pathogen-associated molecular patterns (PAMPs; i.e., LPS), scavenge and phagocytose foreign particles, regulate the synaptic architecture and growth, support neurons and more. Similar to microglia, astrocytes undertake various important functions in the brain, such as the provision of the energy substrate lactate to neurons (“lactate shuttle”) or clearing synapses from excessive excitotoxic glutamate (Smith et al., 2012; Morales et al., 2014; Xu et al., 2021).

Microglia and astrocytes can adopt a pro-inflammatory M1 profile in the presence of oxidative stress, amyloids, infections, injury or during the aging process, as characterized by the upregulation of reduced ionized calcium-binding adapter molecule 1 (IBA-1) (microgliosis) or glial fibrillary acidic protein (GFAP) (astrogliosis). The accumulating DAMPs, including neuron-derived Aβ, Tau, α-synuclein, myelin sheath fragments from damaged axons, neuron-specific enolase, advanced glycation end products (AGEs) and more, as well as PAMPs (i.e., the toll-like receptor 4 (TLR4) ligand LPS result in the activation of TLRs, inflammatory p38 and JNK-signaling, the induction of the master transcription factor NF-κB, the subsequent transcription and release of pro-inflammatory cytokines, such as IL-1β, IL-6, IL-12, interferon γ (IFN-γ) or TNF-α, and chemokines as well as the generation of ROS and inducible nitric oxide synthase (iNOS)-derived NO during AD and PD. In turn, microglia and astrocytes may adopt an anti-inflammatory M2 state to resolve such inflammatory conditions and enhance tissue healing, for example involving the production of IL-4, IL-5, IL-10, IL-13, transforming growth factor β1 (TGF-β) or the neurotrophins BDNF, NGF, and others (Morales et al., 2014; Cortes et al., 2018; Xu et al., 2021).

In AD, oligomeric Aβ was shown to engage with cluster of differentiation (CD)14/TLR2/TLR4 on microglia to induce p38-conveyed ROS-production and the activation of NF-κB (Reed-Geaghan et al., 2009), while the binding of Aβ to CD36 led to the heterodimerisation of TLR4/TLR6 and the NLR family pyrin domain containing 3 (NLRP3) inflammasome-evoked generation of mature IL-1β (El Khoury et al., 2003; Stewart et al., 2010; Sheedy et al., 2013). Aβ fragments further synergised with IFN-γ to exacerbate reactive nitrogen species (RNS) formation and the microglial TNF-α expression (Meda et al., 1995). Similarly, aggregated forms of Tau drive pro-inflammatory cytokine (IL-6) and NO production by microglia (Morales et al., 2013), while in PD, neuron-derived, fibrillated α-synuclein was shown to activate TLR2 (Kim et al., 2013). Aβ and α-synuclein were further shown to trigger inflammatory responses by astroglia, involving, but not limited to, the upregulation of microglia-attractant chemokines (such as monocyte chemoattractant protein-1 or regulated upon activation, normal T Cell expressed and presumably secreted), pro-inflammatory cytokines (IL-1α/IL-1β, IL-6, TNF-α), NF-κB, TLR2, matrix metalloproteinase (MMP)3/9 and ROS/nitrate, with phagocytosed α-synuclein accumulating intracellularly in astrocytes (Johnstone et al., 1999; Lee H.J. et al., 2010; Lim et al., 2013).

Importantly, *in vivo* studies in PD models have demonstrated that the abnormally increased secretion of TNF-α and IFN-γ perpetuates microglial and astroglial neuroinflammation by sustaining the TNF-α/janus kinase/STAT- and IFN-γ/MEK/ERK-mediated activation of NF-κB (Bezzi et al., 2001; Mir et al., 2008; Barcia et al., 2011). Ultimately, chronic neuroinflammation in AD and PD evokes the TNFα and IL-1β-mediated permeabilization of the BBB, immune cell infiltration in to the CNS, mitochondrial and axonal defects, synaptic damage, insulin resistance in the brain as well as cytokine/chemokine/ROS/NO-provoked microglial, astroglial and neuronal dysfunction and death (Smith et al., 2012; Morales et al., 2014; Reich and Holscher, 2020).

### *In vivo* evidence for the anti-inflammatory effects of GLP-1 receptor agonists

Various studies confirm that GLP-1 analogs show potent anti-inflammatory effects. Liraglutide, lixisenatide, semaglutide, exendin-4 or NLY01 [a pegylated and rather poorly BBB-penetrant version of Exendin-4 (Yun et al., 2018; Lv et al., 2021; Park et al., 2021)] reduced IBA-1 or Mac-1 immunoreactivity, indicative of microgliosis, in the hippocampus (Cai et al., 2018; Holubova et al., 2019; Salles et al., 2020; Paladugu et al., 2021; Park et al., 2021), cortex (McClean et al., 2011, 2015; Long-Smith et al., 2013; Paladugu et al., 2021) and global brain tissue (McClean and Holscher, 2014a) of aged WT, APP/PS1, 5xFAD, 3 × Tg-AD or sporadic STZ AD mice. Likewise, microgliosis was attenuated by GLP-1R agonists the SN pars compacta (SNpc) (Kim et al., 2009; Feng et al., 2018; Zhang et al., 2018, 2019, 2020), striatum (Kim et al., 2009; Yun et al., 2018) or cortex (Yuan et al., 2017) of MPTP or α-synuclein-injected mice. In addition, GLP-1 mimetic treatment diminished astrogliosis in the hippocampus (Salles et al., 2018; Paladugu et al., 2021; Park et al., 2021) and cortex (Long-Smith et al., 2013; McClean and Holscher, 2014b; Holubova et al., 2019; Paladugu et al., 2021) of various AD animal models plus the hippocampus (Salles et al., 2018, 2020) of old WT mice, while lessening astroglial GFAP-immunoreactivity in the SNpc, striatum (Yun et al., 2018; Zhang et al., 2018, 2019, 2020; Lv et al., 2021; Zhang L.Y. et al., 2021) or cortex (Yuan et al., 2017) of MPTP-, 6-hydroxydopamine (6-OHDA) or α-synuclein-injected PD rodent models as well as hA53T α-synuclein transgenic mice. In agreement with suppressing glial activity, Exendin-4, NLY01 or liraglutide reduced the expression of NF-κB (Feng et al., 2018; Yun et al., 2018; Lv et al., 2021) and the subsequent synthesis of the pro-inflammatory cytokines IL-1α, IL-1β, IL-6, TNF-α, IFN-γ, and complement component 1q (C1q) in the hippocampus and nigrostriatal regions of various AD and PD animal models (Kim et al., 2009; Feng et al., 2018; Yun et al., 2018; Lv et al., 2021; Park et al., 2021; Zhang L.Y. et al., 2021). Moreover, the expression of anti-inflammatory IL-10 was partially rescued by NLY01 in MPTP-treated animals (Lv et al., 2021).

Similar to GLP-1 mimetics, although superior in their effect, GLP-1R/GIPR receptor dual agonists suppressed microgliosis (Cao et al., 2016; Shi et al., 2017; Feng et al., 2018; Panagaki et al., 2018; Maskery et al., 2020; Salles et al., 2020; Lv et al., 2021), astrogliosis (Cao et al., 2016; Shi et al., 2017; Panagaki et al., 2018; Maskery et al., 2020; Salles et al., 2020; Lv et al., 2021; Zhang L.Y. et al., 2021), the expression of TLR4 (Lv et al., 2021) and NF-κB (Feng et al., 2018; Lv et al., 2021), the production of pro-inflammatory cytokines, such as IL-1β, IL-6, and TNF-α (Feng et al., 2018; Maskery et al., 2020; Lv et al., 2021; Zhang L.Y. et al., 2021), and the restoration of anti-inflammatory TGF-β1 and IL-10 pools (Lv et al., 2021) in the hippocampus, cortex of elderly WT and AD-like animals or the SNpc and striatum of PD rodent models.

### GLP-1 receptor agonists suppress microglial and co-induced astroglial inflammation

Studies have shown that isolated neurons, microglia and astrocytes express GLP-1R (Iwai et al., 2006; Hamilton and Holscher, 2009; Spielman et al., 2017; Yun et al., 2018; Park et al., 2021). While the GLP-1R is widely present on the dendrites of neurons, such as pyramidal neurons in the hippocampal CA1-3 region and neocortex, the granule cell layer of the DG or Purkinje neurons in the cerebellum, the GLP-1R is only sparsely expressed by astroglia and microglia (Hamilton and Holscher, 2009). However, i*n vivo* studies indicate that GLP-1Rs are conditionally upregulated by glial cells following cerebral injury, as further enhanced by GLP-1 analog treatment, seemingly to quench inflammatory responses (Chowen et al., 1999; Lee et al., 2011a). Interestingly, aged mice showed an age-associated decline in the levels of GLP-1 and its precursor proglucagon, but not GLP-1Rs, in the medial prefrontal cortex, further accompanied by heightened numbers of microglia in this brain area, stronger co-localization of microglia with GLP-1Rs and impeded spatial learning. This suggests that attenuated GLP-1 production and, thus, anti-inflammatory GLP-1R activation are linked to cognitive impairments during aging (Ohshima et al., 2015). Notably, cortical and hippocampal proglucagon/GLP-1 was shown to be predominantly derived from microglia and induced by cAMP/PKA-signaling (Kappe et al., 2012). Given that only primary amoeboid microglia, but not ramified (resting) microglia or astrocytes, showed GLP-1 immunoreactivity *in vitro*, it is further implied that the endogenous GLP-1 expression is selectively upregulated by activated microglia to reverse inflammatory responses in the brain (Iwai et al., 2006). However, conditions of insulin resistance during obesity and the chronically increased presence of pro-inflammatory fatty acids were demonstrated to interfere with the microglial proglucagon synthesis and GLP-1 secretion (Kappe et al., 2012).

GLP-1R agonists were shown to directly suppress microglial inflammation, while indirectly forestalling microglia-evoked astroglial activation, to protect neurons from amyloid toxicity. Interestingly, albeit astrocytes were unaffected and neurons showed reduced expression, the treatment of mice with α-synuclein preformed fibrils selectively doubled the mRNA levels of GLP-1R in microglia. Given that GLP-1R levels were 10-fold elevated and predominantly co-localized with IBA-1/transmembrane protein 119 (TMEM119)-positive cells in the SNpc of PD patients, it is implied that GLP-1R is upregulated by pathology-induced microglia during PD. As such, it has been suggested that the predominant site of action of GLP-1 mimetics is mostly microglial (Yun et al., 2018). Notably, the application of α-synuclein fibrils induced the transition into the reactive M1 state and the secretion of IL-1α, IL-1ß, IL-6, C1q, TNFα, and leptin into the culture medium by primary microglia (Yun et al., 2018). Of these, IL-1α, C1q, TNFα, were reported to convert astrocytes into the pro-inflammatory M1 (A1) state, while antibodies for these cytokines blocked the shift of astrocytes into the A1 state and the subsequent death of co-cultured cortical and dopaminergic neurons (Liddelow et al., 2017; Yun et al., 2018). Indeed, when astrocytes were cultured in α-synuclein-containing microglia conditioned medium (MWM), the resulting astrocyte conditioned medium (ACM) was more toxic to primary cortical neurons than MWM. Furthermore, when NLY01 was given to microglia, which led to the reduced secretion of all earlier mentioned pro-inflammatory cytokines, the ACM-driven cell death in primary cortical neurons was prevented. Therefore, given that the direct application of NLY01 to cortical primary neurons failed to rescue them from death by ACM, it was concluded that GLP-1R agonists act on reactive microglia to reduce the secretion of pro-inflammatory cytokines. This, in turn, blocks the conversion of astrocytes into the reactive A1 state, thus forestalling the inflammation-evoked death of neurons. These anti-inflammatory and neuroprotective effects of NLY01, including reduced microglial and astroglial immunoreactivity and IL-1a/b, IL-6, TNFα, and C1qa synthesis, were replicated in α-synuclein-injected rodents and hA53T α-synuclein transgenic mice. As further confirmed, the GLP-1 mimetic was incapable of rescuing neuronal degeneration when GLP-1R was depleted in microglia. Additionally, NLY01 did not block α-synuclein uptake or the inflammatory TLR2/α-synuclein-interaction in microglia, but, instead, suppressed the activation of NF-κB through GLP-1R-signaling (Yun et al., 2018). Comparable to the SNpc of PD patients and the results of the latter study, post-mortem investigations revealed heightened GLP-1R mRNA levels in the hippocampus of AD patients and *in vivo*, likely as a countermeasure to reduce chronic inflammation, whereby GLP-1R was primarily exhibited by IBA-1-positive microglia (Park et al., 2021). As observed for PD cell and animal models (Yun et al., 2018), NLY01 suppressed the Aβ_1–42_-evoked stimulation of hippocampal microglia, the release of multiple pro-inflammatory cytokines in a GLP-1R-mediated manner, forestalled microglia-driven astrocyte activation and the induction of various genes associated with astrogliosis, blocked inflammation/astrocyte-evoked neuronal death *in vitro*/*in vivo* and rescued learning and memory in 5xFAD and 3 × Tg-AD rodents (Park et al., 2021).

As further evidence for an anti-inflammatory and M2-encouraging role, liraglutide prevented irradiation-induced microglial and astroglial immunoreactivity in the cortex and hippocampus, inhibiting the secretion of IL-1β, IL-6, IL-12, and NO (Parthsarathy and Holscher, 2013b). However, when microglia were stimulated with LPS, the effects of Exendin-4 were mixed, either failing to reduce cytokine expression (Ventorp et al., 2017; Wu et al., 2018) or mimicking the effect of anti-inflammatory polyphenols, preventing TNF-α release by microglia (Gullo et al., 2017). Nonetheless, liraglutide suppressed micro-/astrogliosis and shifted reactive M1 microglia toward the anti-inflammatory and ramified M2 phenotype *in vivo*, which dampened palmitate-induced IL-6 and TNF-α synthesis in the DG and hippocampal CA1 (Barreto-Vianna et al., 2017). Indeed, GLP-1R activation on primary microglia led to the expression of M2-associated markers, including IL-4 (cAMP/PKA/CREB-dependent) and Arg 1 and CD206 (alternative Gs-cAMP/PKA/p38β/CREB-mediated). Notably, GLP-1R agonism selectively induces the non-inflammatory p38β isoform and β-endorphin expression, but does not stimulate the pro-inflammatory isoform p38α, in microglia (Bachstetter et al., 2011; Wu et al., 2017, 2018).

### GLP-1 prevents glial inflammation in an indirect manner

Generally, *in vivo* studies imply that reactive microglia (Long-Smith et al., 2013; Salles et al., 2018) and astrocytes (Long-Smith et al., 2013; Holubova et al., 2019) are induced by and gather around Aβ plaques, co-localizing with markers of insulin resistance in neurons (Long-Smith et al., 2013). In turn, GLP-1R agonist treatment was repeatedly shown to diminish the total soluble Aβ, Aβ oligomer or cerebral plaque burden in AD-like rodents (summarized in section “GLP-1R agonists are neuroprotective and prevent amyloid beta accumulation *in vivo”*), reduce α-synuclein accumulation in PD animal models (Yun et al., 2018; Zhang et al., 2019; Zhang L.Y. et al., 2021) and re-sensitize the neuronal insulin sensitivity *in vitro* (Jantrapirom et al., 2020) and in the brains of AD and PD *in vivo* models (see also section “Insulin resistance and the neuronal energy metabolism”) (Long-Smith et al., 2013; Shi et al., 2017; Batista et al., 2018; Paladugu et al., 2021; Zhang L.Y. et al., 2021). This suggests that GLP-1R agonists forestall neuroinflammation by preventing insulin resistance and the mutually exacerbated amyloid pathology.

Moreover, besides quenching microgliosis and cytokine production, Exendin-4 downregulated the expression of MMP3 in the SNpc of MPTP-treated mice (Kim et al., 2009). MMP3 represents a DAMP that is released by apoptotic neurons, similar to α-synuclein. *In vitro* studies suggest that cell stress-induced apoptosis enhances the expression of the pro-form of MMP3 in dopaminergic neurons, which is cleaved into MMP3 following pro-apoptotic JNK-signaling by serine proteases (Choi et al., 2008). MMP3 further accelerates α-synuclein fragmentation and aggregation in dopaminergic neurons, co-localizing with Lewy bodies in the brains of PD patients (Choi et al., 2011). Importantly, MMP3 that is liberated by dying neurons stimulates ERK/NF-κB-signaling, pro-inflammatory cytokine (IL-1β, IL-6, TNFα) and nicotinamide adenine dinucleotide phosphate oxidase-mediated superoxide production by nearby microglia. Indeed, MPTP-induced microglial inflammation, ROS, BBB damage, cerebral immune cell infiltration and dopamine neuron death in the nigrostriatal region were largely attenuated or fully prevented in MMP3^–/–^ mice (Kim et al., 2005, 2007; Chung et al., 2013). In the context of AD, the expression of MMP3 was also upregulated by the PI3K/Akt pathway in microglia in response to Aβ_1–42_ (Ito et al., 2007), while MMP3 was shown to correlate with total and phospho-Tau levels in the CSF of AD patients (Stomrud et al., 2010). Notably, MMP3 cleavage attenuates Tau aggregation, whereas MMP9, as activated by MMP3, promotes Tau oligomerization (Wang et al., 2014).

Additional evidence indicates that the GLP-1R inhibits inflammatory cascades indirectly. A recent study showed that the application of Aβ_25–35_ resulted in NF-κB and NLRP3-mediated inflammatory cytokine expression, while the inhibition of TLR4 prevented the pathological microglial transition from M2 to M1 *in vitro* and in the APP/PS1 mouse model (Cui et al., 2020). Indeed, TLR4 plays a critical pathological role, given that a TLR4 polymorphism was shown to attenuate the risk of late-onset AD 2.7-fold (Minoretti et al., 2006). In this context, the GLP-1R/GIPR dual agonist DA5-CH downregulated TLR4 expression in the MPTP mouse model (Lv et al., 2021), while MPTP was shown to upregulate TLR4 levels by inducing the transcription factor AP-1 *in vivo* (at least in astrocytes) (Roger et al., 2005; Zhao et al., 2016). Furthermore, as a positive feedback loop, TLR4/NF-κB-signaling (i.e., as stimulated by AβCui et al., 2020) potentiate the transcription of TLR4 under inflammatory conditions (Li et al., 2019). Synoptically, by suppressing oxidative stress, mitochondrial dysfunction and apoptosis in neurons, the release of death-associated DAMPs that would otherwise stimulate TLRs are reduced (see section “Oxidative stress and mitochondrial dysfunction” and Figure 2). Moreover, the activation of the GLP-1R receptor quenches oxidative stress in glial cells (see section “GLP-1 exerts direct anti-inflammatory and cytoprotective effects on astrocytes” for the cytoprotective effects in astrocytes), thus forestalling the stress-provoked induction of NF-κB and AP-1, the concomitant expression of TLR4/pro-inflammatory cytokines and the inflammatory switch from M2 to M1 in glial cells.

Notably, inflammasome activation and the associated synthesis of NLRP3, caspase 1 and IL-1β are triggered by the Aβ/TLR4/NF-κB pathway in microglia (Boaru et al., 2015; Liu et al., 2020). Interestingly, even though liraglutide did not affect the interaction of α-synuclein with TLR2 on microglia, the activation of the GLP-1R suppressed NF-κB activation in response to α-synuclein and MPTP-driven oxidative stress in neurons *in vivo* (Feng et al., 2018; Yun et al., 2018; Lv et al., 2021). Notably, GLP-1R activation also suppressed TNFα- or LPS-driven cell death, ROS and NO production, while upregulating antioxidant gene expression and protein levels of glutathione peroxidase 1 (GPx1) and superoxide dismutase (SOD)1 in a cAMP/PKA-, but not PI3K/Akt-, dependent manner in various microglial cell lines (Spielman et al., 2017). Such studies strengthen the concept that GLP-1 mimetics exert indirect anti-inflammatory effects on microglia and astrocytes by protecting glial cells (and neurons) from oxidative and amyloid-driven injury.

### GLP-1 exerts direct anti-inflammatory and cytoprotective effects on astrocytes

Multiple studies confirm beneficial consequences of GLP-1R activation on astrocytes. The application of GLP-1 induced the cAMP/CREB pathway in astrocytes to inhibit the LPS-provoked transcription and release of IL-1β, with trends for reduced IL-6 and iNOS, in a cAMP-dependent manner (Iwai et al., 2006). Indeed, pro-inflammatory cytokine production, including IL-1ß and TNFα, was downregulated by and dependent on CREB activation in primary cortical astrocytes (Zhao and Brinton, 2004). Moreover, GLP-1 mimetics show clear protective effects. Liraglutide, by activating GLP-1R and recovering cAMP/PKA/CREB signaling, dose-dependently reducde ROS production, blocked pro-apoptotic caspase 3 cleavage, raised cell viability and blocked IL-1ß and TNFα secretion by cultured rat cortical astrocytes following AGE treatment (Bao et al., 2015). In this context, AGEs were shown to trigger oxidative stress and a reduction in anti-oxidative modulators in astrocytes *in vitro*, as marked by diminished GSH and SOD and elevated malondialdehyde (MDA), monoamine oxidase B and NO levels, leading to pro-inflammatory cytokine production (Wang et al., 2002; Jiang et al., 2004). Additionally, liraglutide activated the cAMP/PKA pathway in astrocytes, which, in a PKA-dependent manner, reverted the Aβ_1–42_-induced downregulation of the mitochondrial fusion enhancers mitofusin-2 (Mfn2), optic atrophy 1 (OPA1) and the fission-inhibiting phosphorylation of dynamin-1-like protein 1 (DLP1, also known as DRP1) at Ser^637^ by PKA. The GLP-1R agonist further prevented Aβ_1–42_-driven mitochondrial fragmentation, normalized the collapsed mitochondrial membrane potential, rescued proton leakage, enhanced ATP production, reduced ROS generation and improved astroglial survival *in vitro* (Xie et al., 2021). Notably, the mitochondrial fusion/fission-ameliorating properties of GLP-1 mimetics in astrocytes are comparable to those in Aβ-assaulted neurons (see section “GLP-1R agonists suppress amyloid beta and GSK-3β-driven mitochondrial damage in AD” and Figure 2).

Interestingly, in contrast to Aβ-induced mitochondrial fragmentation, the opposite might be the case in PD. Astrocytes and neurons isolated from the SNpc of PD patients showed an early decrease in DLP-1 activity (Hoekstra et al., 2015), while another study identified an over twofold reduction of DLP-1 in mitochondria-enriched fractions of the SNpc of PD patients (Jin et al., 2006). In this context, albeit not accounting for any possible defects in mitochondrial fusion proteins, the deletion of astroglial DLP-1 led to mitochondrial elongation, fusion and increased motility, intracellular Ca^2+^ amassment and impaired glutamate removal, which resulted in excitotoxic injury of co-cultured dopaminergic neurons *in vitro* (Hoekstra et al., 2015). Therefore, it would be interesting to assess the effects of GLP-1R activation on fusion/fission modulators in astrocytes and neurons in future PD studies.

Besides cAMP/PKA-induced cellular protection, GLP-1-triggered PI3K/Akt-signaling prevented Aβ_1–42_-provoked increases in oxygen consumption, ATP and ROS production as well as a detrimental switch from aerobic glycolysis to excessive OXPHOS in astrocytes *in vitro* and *in vivo* (Zheng et al., 2021). Notably, in contrast to OXPHOS-utilizing neurons, astrocytes prefer aerobic glycolysis (which is coupled to lactate production) as main bioenergetic pathway. On the other hand, astrocytes compensate with OXPHOS in response to bioenergetic and mitochondrial dysfunction (inverse Warburg effect) (Bolanos et al., 2010; Demetrius and Simon, 2013).

Synoptically, by stimulating the cAMP/PKA/CREB pathway in astrocytes, GLP-1R agonists elicit direct anti-inflammatory effects. Additionally, GLP-1 induces cytoprotective cAMP/PKA- and PI3K/Akt-signaling that forestalls pro-inflammatory responses by astrocytes in response to oxidative stress, mitochondrial dysfunction, bioenergetic deficits and/or pro-apoptotic signaling.

### GLP-1 enhances the neurosupportive function of astrocytes

Interestingly, by improving the astroglial energy metabolism and survival, GLP-1 analogs enhance the neuronal supply with lactate, BDNF and GSH, while possibly preventing excitotoxic damage. As implied in the previous section, GLP-1 prevented the Aβ_1–42_-induced mitochondrial dysfunction and the compensatory switch from the lactate-producing aerobic glycolysis, the main bioenergetic pathway in astrocytes, to the oxidative stress-associated OXPHOS in cultured astrocytes. Indeed, in a PI3K/Akt-dependent manner, GLP-1 prevented the downregulation of glycolytic enzymes [pyruvate kinase (PKM), PKM2, hexokinase 2 (HK2), pyruvate dehydrogenase kinase 2 (PDK2)], the transcription factor hypoxia-inducible factor 1 alpha (HIF-1α), lactate and NAD^+^ levels as well as the NAD^+^/NADH ratio in Aβ_1–42_-assaulted astrocytes. Similarly, 5xFAD mice showed elevated ROS generation, diminished redox capabilities (GSH), impaired ATP production, the alteration of copious bioenergetic genes, the reduced synthesis of glycolytic enzymes and excessive OXPHOS, as characterized by the abnormally heightened phosphorylation of the OXPHOS-inducer pyruvate dehydrogenase (PDH), and reduced activity of the PDH-modifier PDK (Zheng et al., 2021). The latter restricts the PDH-mediated conversion of pyruvate into Acetyl-CoA for OXPHOS in mitochondria and stimulates the conversion of pyruvate into lactate by lactate-dehydrogenase (LDH) in the cytoplasm instead (Jha et al., 2016). All of the latter bioenergetic impairments could be reversed with liraglutide injections *in vivo* which, as observed *in vitro*, led to the stimulation of Akt (Zheng et al., 2021). In this context, Akt stimulates the mTOR/HIF–1α pathway (Cheng et al., 2014; Carlessi et al., 2017), with HIF–1α controlling the expression of the forementioned glycolytic enzymes as well as PDK and LDH-A (an isoform enriched in astrocytes and weakly expressed in neurons), thus favoring the processing of glucose into pyruvate and lactate (Jha et al., 2016). As a side note, astrocytes generally express higher levels of PDK_2/4_ and LDH than neurons, explaining the preference of astrocytes for aerobic glycolysis (Jha et al., 2016). Indeed, although failing to affect brain glucose metabolism in 3 × Tg AD mice, the administration of Exendin-4 led to elevated LDH activities (likely in astrocytes), pyruvate/lactate conversion and lactate levels in the brains of PS1K-KI mice, as accompanied by enhanced short- and long-term spatial memory (Bomba et al., 2013).

Interestingly, Aβ-stressed astrocytes decreased the viability of co-cultured primary neurons, whereas astroglia GLP-1 treatment not only prevented neuronal death, but also enhanced the axonal and dendritic outgrowth and synapse formation. In turn, the latter favorable effects of GLP-1 on neurons were inhibited by blocking astroglia glycolysis with 2-DG (Zheng et al., 2021). Liraglutide further restored the secrteion of BDNF by Aβ_1–42_-stressed primary astrocytes in a PKA-dependent manner. Importantly, the GLP-1 mimetic only forestalled the astrocyte-induced death of co-cultured primary neurons and improved the neuronal numbers of neuritic interactions, somal neurites, secondary branches and total axon plus neurite length when Aβ_1–42_-assaulted astrocytes were treated with liraglutide in the absence of a PKA inhibitor (Xie et al., 2021). Thus, in the presence of amyloid pathology, GLP-1 analogs seem to stimulate the general energy turnover, the cAMP/PKA/CREB/BDNF pathway and BDNF secretion by astrocytes to evoke BDNF-driven synaptogenesis and neurite outgrowth in neurons.

Moreover, the astroglia and NRF-2-mediated enzyme expression for the production and release of the antioxidant GSH, whose levels were restored by liraglutide in the cortex of 5xFAD mice (Zheng et al., 2021), are crucial for the shielding of nearby neurons from oxidative damage (Bolanos, 2016).

Therefore, GLP-1 protects astrocytes from amyloid toxicity and enhances the supportive function of astrocytes toward neurons. This involves the PI3K/Akt/mTOR/HIF–1α-dependent downregulation of OXPHOS-triggered oxidative stress, the re-invigoration of glycolysis and the neuronal lactate shuttle, the cAMP/PKA/CREB-driven synthesis and liberation of pro-synaptic and neuroprotective BDNF as well as the provision of anti-oxidative GSH by astrocytes (Xie et al., 2021; Zheng et al., 2021).

Notably, Aβ disturbs the homeostasis between astrocytes and neurons by impairing the astroglia expression of glutamate transporters excitatory amino acid transporter 1 (EEAT1) and EAAT2, which impedes the clearance of glutamate from the synaptic cleft, and glutathione synthase (GS), an enzyme that converts glutamate into glutamine. This leads to the accumulation of excitotoxic glutamate in the synaptic space and the reduced neuronal supply with glutamine, which is a crucial bioenergetic and neurotransmitter substrate for neurons (Acosta et al., 2017). Intriguingly, while Aβ_1–42_ diminished glutamate uptake, EEAT2 and GS levels, GLP-1 could restore the astroglia expression of GS in an *in vitro* study (Xie et al., 2021). This indicates that the pro-metabolic and neuroprotective effects of GLP-1R agonists on astrocytes may protect neurons from excitotoxicity.

Notably, GLP-1R activation on neurons directly prevents excitotoxicity (section “GLP-1 mimetics suppress Ca2+ deregulation by amyloid beta and excitotoxicity”). Indeed, the use of GLP-1 analogs rescued from the kainite- or ibotenic acid-driven excitotoxic apoptosis of hippocampal and basal forebrain cholinergic neurons *in vivo* (Perry et al., 2002a; During et al., 2003). An *in vitro* study indicated that the neuroprotective effects of GLP-1 were linked to the blockade of glutamate and VDCC currents, the associated Ca^2+^ instream and membrane depolarisation in response to exogenous neuronal glutamate overload (Gilman et al., 2003). For other excitotoxicity-suppressing effects of GLP-1 in the context of epilepsy, see also (Koshal et al., 2018).

## GLP-1 mimetics rescue nigrostriatal dopamine neuron death and dopamine depletion in Parkinson’s disease

In PD, mitochondrial dysfunction may arise as a consequence of gene mutations that exacerbate oxidative stress, impair the mitochondrial function and impede ATP generation, such as DJ1, leucine-rich-repeat kinase 2 (LRRK2) or the mitophagy-associated modulators PTEN-induced kinase 1 (PINK1) and Parkin. Other hypothesized reasons for mitochondrial damage include ROS-triggered mtDNA damage, environmental toxins that interfere with OXPHOS or non-mutant α-synuclein accumulation, which seems to enhance ER-mitochondria interactions and, hence, cause excessive Ca^2+^ transfer to mitochondria. It is thought that SNpc-located dopaminergic neurons selectively degenerate during PD due to their high energy demands, a concomitant increase in ROS production, the synthesis of autoreactive catecholamines (dopamine), poor anti-oxidant and Ca^2+^-buffering capabilities as well as weakly or non-myelinated axons (Sulzer and Surmeier, 2013; Bose and Beal, 2016). In this context, PD models rely on the use of complex I inhibitors, such as MPTP, rotenone or 6-OHDA, that are selectively taken up by dopaminergic neurons and interfere with the mitochondrial ATP production by the ETC, while elevating ROS generation (Zeng et al., 2018). Given that an estimated ∼30% of dopaminergic neurons in the SNpc, ≤ ∼60% of the external SNpc projections, especially toward the striatum, and ≤ ∼70% of the dopamine supply of the dorsal striatum are lost when the characteristic motor symptoms occur in PD (Cheng et al., 2010), it is evident that pharmacological interventions must ensue early.

There is abundant *in vivo* evidence that GLP-1R activation protects nigrostriatal neurons and replenishes the production of dopamine in PD. Various synthetic incretin analogs, such as exendin-4, liraglutide, lixisenatide, semaglutide, (Val8)GLP-1-Glu-PAL, NLY01, or GLP-1R/GIPR dual agonists, were shown to prevent the atrophy of dopaminergic neurons in the SN and striatum (Oh et al., 2006; Bertilsson et al., 2008; Harkavyi et al., 2008; Kim et al., 2009; Li et al., 2009; Liu et al., 2015a; Zhang et al., 2015, 2018, 2020; Cao et al., 2016; Ji et al., 2016; Jalewa et al., 2017; Yun et al., 2018; Lv et al., 2021; Zhang L.Y. et al., 2021) and preserve dopaminergic fibers in PD animal models (Bertilsson et al., 2008; Kim et al., 2009). Moreover, these GLP1R and GLP1R/GIPR dual agonists restored the nigral expression of TH, the rate-limiting enzyme of dopamine-synthesis (Bertilsson et al., 2008; Harkavyi et al., 2008; Li et al., 2009, 2020c; Liu et al., 2015a; Zhang et al., 2015, 2018, 2019, 2020; Cao et al., 2016; Ji et al., 2016; Jalewa et al., 2017; Yuan et al., 2017; Feng et al., 2018; Yun et al., 2018; Lv et al., 2021; Zhang L.Y. et al., 2021), dopamine transporter (Yun et al., 2018), monoamine transporter 2 and vesicular monoamine transporter 2 (VMAT2) (Bertilsson et al., 2008), while improving the production of the dopamine precursor l-3,4-dihydroxyphenylalanine (L-DOPA), dopamine, the pools of other dopamine metabolites [dihydroxyphenylacetic acid (DOPAC), homovanillic acid (HVA), and 3-methoxytyramine (3MT)], dopamine turnover (Harkavyi et al., 2008; Li et al., 2009, 2020c; Jalewa et al., 2017; Yun et al., 2018; Zhang L.Y. et al., 2021) as well as norepinephrine and the serotonin breakdown product 5-hydroxyindoleacetic acid (5-HIAA) (Li et al., 2020c) in the striatum or basal ganglia of MPTP, 6-OHDA, rotenone, α-synuclein pre-formed fibril, or LPS-induced mouse models of PD. Interestingly, Exendin-4 massively increased TH expression (60%) even in the absence of 6-OHDA *in vitro*, implying that GLP-1 not merely protects TH-expressing dopaminergic neurons, but also improves dopamine synthesis on a transcriptional level (Li et al., 2009). GLP-1R or GLP-1R/GIPR dual agonists further decreased the production of the lipid peroxidation product and oxidative stress marker 4-hydroxynonenal (4-HNE) (Zhang et al., 2018, 2019, 2020), raised the synthesis of the neurotrophins glial cell line-derived neurotrophic factor (GDNF) (Jalewa et al., 2017; Yuan et al., 2017; Feng et al., 2018; Zhang et al., 2019; Lv et al., 2021) and BDNF (Ji et al., 2016; Lv et al., 2021) (more details in section “Other growth factors”) and prevented the accumulation of α-synuclein (Zhang et al., 2019; Lv et al., 2021; Zhang L.Y. et al., 2021), phospho-α-synuclein and insoluble α-synuclein in TH-positive striatal and midbrain dopaminergic neurons *in vivo* (Yun et al., 2018).

Mechanistically, GLP-1R activation enhanced the phosphorylation of Akt (Ji et al., 2016; Jalewa et al., 2017), induced CREB (Jalewa et al., 2017), heightened the synthesis of anti-apoptotic Bcl-2 (Liu et al., 2015a; Zhang et al., 2015, 2018; Ji et al., 2016; Lv et al., 2021), reduced the levels of pro-apoptotic Bax (Liu et al., 2015a; Zhang et al., 2015, 2018; Ji et al., 2016; Li et al., 2020c; Lv et al., 2021) and CytC (Li et al., 2020c), normalized the lowered Bcl-2/Bax ratio (Ji et al., 2016; Zhang et al., 2018; Lv et al., 2021) and diminished the levels of the apoptosis-effector caspase 3 (Zhang et al., 2015; Li et al., 2020c; Lv et al., 2021) in the nigrostriatal brain region of PD rodent models. We will investigate the underlying neuroprotective pathways in section “Oxidative stress and mitochondrial dysfunction.”

In agreement with their neuroprotective effects, GLP1 or GLP1/GIP dual modulators preserved the motor function of the PD animals, including better balance and motor coordination in the rotarod (Li et al., 2009, 2020c; Liu et al., 2015a; Zhang et al., 2015, 2018; Cao et al., 2016; Ji et al., 2016; Jalewa et al., 2017; Yuan et al., 2017; Feng et al., 2018; Yun et al., 2018; Lv et al., 2021) or pole test (Li et al., 2009; Yun et al., 2018), improved grip strength (Cao et al., 2016; Ji et al., 2016; Yuan et al., 2017; Feng et al., 2018), lowered motor activity in the swimming test (Zhang et al., 2015), lessened gait and postural abnormalities in the footprint or gait test (Zhang et al., 2018; Lv et al., 2021), heightened spontaneous locomotor behavior in the open field test (Li et al., 2009, 2020c; Liu et al., 2015a; Zhang et al., 2015, 2018; Cao et al., 2016; Jalewa et al., 2017; Lv et al., 2021; Zhang L.Y. et al., 2021) decreased rigidity in the catalepsy trial (Liu et al., 2015a), normalized grooming behavior and rearing (Yun et al., 2018), improved sensory motor function in the cylinder test (Yun et al., 2018) as well as accelerated functional recovery, as indicated in the apomorphine/amphetamine tests (Bertilsson et al., 2008; Harkavyi et al., 2008; Jalewa et al., 2017; Yun et al., 2018; Zhang L.Y. et al., 2021). The latter animal studies showed that GLP-1/GIPR dual agonists had superior effects compared to GLP-1R analogs (Yuan et al., 2017; Feng et al., 2018; Zhang et al., 2020; Lv et al., 2021; Zhang L.Y. et al., 2021).

Strikingly, as a proof of concept, Exendin-4 preserved the motor abilities of PD patients in a randomized and double-blind phase II clinical trial, wherein the beneficial effects were still visible after a 12 week washout phase (Athauda et al., 2017). Exosome analysis indicated that these PD patients showed elevated Ser-phosphorylated IRS-1 levels and impaired insulin-signaling in the brain. In turn, the application of Exendin-4 improved the impeded insulin sensitivity, as implied by enhanced Akt and phospho-mTor levels in treated PD patients. Indeed, the motor improvements in these PD patients were positively associated with the levels of mTor and phopho-mTor levels (Athauda et al., 2019). Given the rising acknowledgment of insulin resistance as an early pathological key step in AD and PD, we have devoted a stand-alone section to the insulin-re-sensitizing mechanisms of GLP-1R agonists (see section “Insulin resistance and the neuronal energy metabolism” and Figure 1).

## Oxidative stress and mitochondrial dysfunction

### GLP-1 receptor-signaling protects from external oxidative stress, reactive oxygen species production and the mitochondrial apoptosis pathway

Mitochondria may produce ATP by several pathways. While it has yet to be proven that neurons utilize lipid β-oxidation, they preferably metabolize glucose via glycolysis to create pyruvate. The latter is subsequently funneled into the tricarboxylic acid (TCA) cycle to generate a few units of ATP and, importantly, reduce nicotinamide adenine dinucleotide (NAD^+^) to NADH. In turn, NADH participates in OXPHOS that is executed by complex I–V of the ETC. Briefly, electrons (e^–^) won from NADH at complex I are moved toward complex IV within the inner-mitochondrial membrane, resulting in the reaction of e^–^ and H^+^ to H_2_O at complex IV. Simultaneously, H^+^ ions are pumped outwards across complex I, III, and IV from the inner matrix to the intermembrane space. This establishes a gradient, leading to the re-flux of H^+^ ions into the inner matrix through complex V (ATP synthase) to produce ATP. Importantly, e^–^ may leak at complex I and III, but also IV. ∼1–2% of the consumed oxygen reacts with these escaping e^–^ to evoke the generation of the O_2_^–^. The latter ROS may be converted by SOD1 or SOD2 into H_2_O_2_, followed by detoxification into H_2_O by mitochondrial GPx or cytosolic catalase. As such, ironically, mitochondria pose the greatest cellular source of oxidative stress (West et al., 2011; Tracey et al., 2018).

As death signals accumulate in response to mitochondrial defects, including elevated ROS production, bioenergetic deficiencies, loss of ATP production and Ca^2+^ deregulation, pro-apoptotic Bcl-2 proteins are induced. Generally, anti-apoptotic members of the Bcl-2 family, for example Bcl-2 or Mcl-1, sequester their pro-apoptotic counterparts, including Bad, Bax, Bak, Bid, Bik, and others, on the outer mitochondrial membrane (but also ER) to manage the cellular survival. However, cellular and mitochondrial dysfunction stimulate pro-apoptotic Bcl-2 family members, mitochondrial pore formation by Bax and Bak, permeabilization of the outer mitochondrial membrane, the release of pro-apoptotic factors, especially CytC, and the sequential activation of APAF-1, caspase 9 and caspase 3 to drive intrinsic apoptosis. Notably, as an alternative, cells may be subject to extrinsic apoptosis across caspase 8 and caspase 3, as driven by ligand binding to death receptors on the cellular membrane, including Fas/Fas ligand (FasL) and tumor necrosis factor receptor-1/TNF-α (Youle and Strasser, 2008; Lindsay et al., 2011; Wu et al., 2019).

Ample evidence shows that GLP-1R or GLP-1R/GIPR dual activators guard against external oxidative stress, while preventing internal ROS production by injured mitochondria. *In vitro*, GLP-1, liraglutide, Exendin-4 and GLP-1R/GIPR dual agonists shielded PC12 neuronal, RGC-5 retinal and SH-SY5Y cells from H_2_O_2_-induced oxidative damage (Li et al., 2010c,^2015^; Luciani et al., 2010; Chen et al., 2012; Ma et al., 2017; Salles et al., 2020) or Aβ-driven oxidative stress in a GLP-1R-dependent manner (Li et al., 2010b). Moreover, incretin hormones prevented H_2_O_2_-provoked mitochondrial injury and consequential ROS production by the mitochondria (Ma et al., 2017; Salles et al., 2017, 2020). Indeed, TEM images confirmed that Liraglutide prevented mitochondrial swelling and the disintegration of cristae upon H_2_O_2_ exposure in RCG-5 cells (Ma et al., 2017). In PD-specific contexts, with GLP-1R/GIPR dual agonists showing the best effects, GLP-1R induction by various incretin mimetics rescued SH-SY5Y cells and primary hippocampal, cortical or dopaminergic neurons from 6-OHDA or rotenone-provoked mitochondrial complex I dysfunction, the associated intercellular ROS accumulation and apoptosis (Li et al., 2009, 2010c,2020c; Jalewa et al., 2016; Zhang L.Y. et al., 2021).

As illustrated in Figure 2, a recent study in GLP-1-treated HT22 hippocampal cells indicated that the neuroprotective effects of incretin hormones on H_2_O_2_-induced oxidative stress and mitochondrial dysfunction, glutamate overload, tunicamycin or thapsigargin-triggered ER stress or Aβ_1–42_-provoked neuronal death involve the activation of Akt and ERK_1/2_ (Yoshino et al., 2015). More specifically, GLP-1R induction blocks caspase-dependent apoptosis through the mitochondrial pathway by modulating Bcl-2 family members (Li et al., 2015). As supported by various studies, the neuroprotection from H_2_O_2_- or mitotoxin-induced oxidative stress necessitated the GLP-1R-induced activation of PI3K/Akt and PKA, resulting in attenuated levels of each member of the pro-apoptotic Bax/CytC/Caspase 3 pathway, decreased Bad phosphorylation, increased anti-apoptotic Bcl-2 expression and phosphorylation, elevated Bcl-2/Bax ratios and raised TH synthesis (Li et al., 2010c,2020c; Jalewa et al., 2016; Salles et al., 2018; Zhang et al., 2020; Zhang L.Y. et al., 2021).

Indeed, PI3K/Akt and ERK_1/2_, as induced by growth hormones and neurotrophins such as insulin, GLP-1 or BDNF, are well-known survival pathways that are deregulated in neurodegenerative diseases. Neuroprotective PI3K/Akt-signaling activation enhances mTor/ribosomal protein S6 kinase beta-1 (S6K)-mediated protein translation and cell proliferation, which involves the Akt-mediated inactivation of the translation repressor eukaryotic translation initiation factor 4 (eIF4E)-binding protein (4E-BP) (Rai et al., 2019; Hölscher, 2020). This general elevation in protein translation is likely responsible for the increase in TH expression (60%) that has been observed in GLP-1 mimetic-treated primary mesencephalic cell cultures (which are enriched in dopaminergic neurons) *in vitro* (Li et al., 2009). Akt further activates Raf/MEK_1/2_ to reinforce ERK activity and phosphorylates CREB to drive the expression of the anti-apoptotic effectors Bcl-2 and Mcl-1. Furthermore, Akt phosphorylates and inhibits (i) pro-apoptotic Bad, a counter-regulator of the pro-survival mediator Bcl-2, (ii) caspase 9, which is the upstream activator of caspase 3, (iii) GSK-3β, a major apoptosis pathway in neurons and (iV) death-associated FOXOs that upregulate the apoptosis-inducing FasL, whilst (V) suppressing the pro-apoptotic JNK/p53/Bax axis (Pugazhenthi et al., 2000; Walton and Dragunow, 2000; Mori et al., 2004; Rai et al., 2019; Hölscher, 2020). On the other hand, while chronic ERK_1/2_-signaling alone, similar to its serine/threonine protein kinase family members JNK and p38, may exert pro-apoptotic effects in some instances, for example during PD (Kulich and Chu, 2001; Gomez-Santos et al., 2002), ERK_1/2_ induces CREB-driven plasticity and survival gene expression (Hermann et al., 2000; Rai et al., 2019; Hölscher, 2020). Notably, ERK further phosphorylates GSK-3β at Thr^43^. This primes GSK-3β for the inactivating Ser^9^ phosphorylation by p90RSK, followed by the upregulation of β-catenin (Ding et al., 2005).

In the context of oxidative stress, the neuroprotective effects of GLP-1 involved the upregulation of the transcription factor ATF4, which mediates the expression of anti-oxidative and redox genes, for example GSH (Harding et al., 2003; Li et al., 2010c). Other studies suggest that GLP-1 agonists enhance the levels and activity of ATF4 through cAMP/PKA-signaling to prevent oxidative injury, as observed in response to cytosolic Ca^2+^ overload or ER stress (Elefteriou et al., 2005; Yusta et al., 2006). A study in H_2_O_2_-stressed human umbilical vein endothelial cells reported that Exendin-4, by activating the GLP-1R/cAMP/PKA pathway, evoked the CREB-mediated transcription of the antioxidant defense genes heme oxygenase 1 (HO-1) and NAD(P)H Quinone Dehydrogenase 1 (NQO-1) (Oeseburg et al., 2010). Studies in methyl glyoxal-injured PC12 cells further indicate that GLP-1 ameliorates the cellular redox balance by stimulating the PI3K/Akt/mTOR-dependent transcriptional upregulation of glutamate-cysteine ligase catalytic subunit (GCLc), the rate-limiting enzyme for GSH synthesis (Kimura et al., 2009). Indeed, *in vitro* studies in GLP-1R-expressing mesenchymal stem cells support that GLP-1 analogs, as dependent on PI3K/Akt, dose-dependently shield against H_2_O_2_-driven loss of mitochondrial membrane potential, thus forestalling ROS production by defective mitochondria, intrinsic apoptosis across the mitochondrial pathway (increased Bcl-2 and reduced Bax, caspase 9/3 induction and CytC release) and upregulate the expression of SOD and the anti-oxidant GSH, while preventing ROS-associated lipid peroxidation and MDA formation (Zhou et al., 2014).

Neuroprotection in PD further involves the suppression of JNK by incretins. In this context, elevated levels of pJNK have been detected in the SN of PD rodent models and patients. Moreover, MPTP-induced dopamine neurons death necessitated JNK_2/3_/COX2-signaling, which seemingly enhances neurotoxicity by eliciting the COX2-induced oxidation of dopamine to create highly redox-reactive dopamine-quinones (Teismann et al., 2003; Hunot et al., 2004). In PD, JNK drives neuronal death across the mitochondrial intrinsic (Bax/CytC/caspase 3) and extrinsic (c-Jun/AP-1/FasL and more) apoptosis pathways. JNK is activated in response to various forms of stress in neurons, including oxidative stress, as reinforced by enhanced ROS-production from dysfunctional mitochondria or complex I inhibition by PD-toxins, and the induction of neuronal PICRs by the glia-derived pro-inflammatory cytokines IL-1 and IL-18 (Wang et al., 2012; Ojala and Sutinen, 2017; Kheiri et al., 2018). This suggests that GLP-1 agonists suppress the pro-apoptotic activation of JNK in dopaminergic neurons by enhancing anti-oxidative mechanisms (as elucidated below), preventing ROS-generation due to mitochondrial dysfunction and quenching microglial and astroglia inflammation during PD (more insight in section “Inflammation”). Indeed, GLP-1R-signaling prevented JNK activation following rotenone treatment *in vitro/in vivo*, whereas the pharmacological enhancement of JNK interfered with the GLP-1R-driven Akt phosphorylation and exacerbated the induction of the CytC/Bax/Caspase 3 cell death axis (Li et al., 2020c).

Synoptically, in light of oxidative stress and summarized in Figure 2, GLP-1 agonists engage PI3K/Akt to suppress effectors of mitochondrial apoptosis pathway and, indirectly, pro-apoptotic JNK-signaling, induce the cAMP/PKA/ATF4 pathway to stimulate anti-oxidative gene transcription (i.e., GSH) and activate ERK_1/2_ and CREB for the transcription of survival and antioxidant defense genes (such as HO-1 and NQO-1). The latter anti-oxidative properties following GLP1R activation, in turn, forestall mitochondrial injury through oxidative stress and concomitant ROS-production as well as deficits in ATP production.

### GLP-1 analogs protect from iron and dopamine-induced oxidative stress and ferroptosis

The deposition of metal ions in the brain might contribute to oxidative stress in AD and PD. A major question is what triggers iron accumulation in the first place. In this context, chronic neuroinflammation may be an initiating trigger during AD and PD. Generally, iron is present in a bound form, as sequestered by both intracellular and macrophage-secreted ferritin in the blood plasma, as well as a free form (Fe^2+^), also known as labile iron pool (LIP). Free iron co-mediates essential cellular processes, such as mitochondrial respiration or the synthesis of nucleic acids. Iron is an essential co-factor for pathogens. As such, under inflammatory conditions, the release of pro-inflammatory cytokines alters the expression of iron-associated genes in the mononuclear phagocyte system to sequester iron in macrophages (including microglia). For instance, ROS, IL-1β and, in particular, IL-6, stimulate the STAT3-mediated expression and secretion of hepcidin antimicrobial peptide (HAMP), which is secreted, binds to and initiates the proteasomal degradation of the iron exporter ferroportin 1 (FPN1) in in surrounding cells. TNF-α and IFN-γ further elevate the transcription of the ion-importer transferrin receptor 1 (TFR1) by macrophages. While IL-1β and IL-6 further promote ferritin expression in macrophages to induce the storage of iron, ferritin, in both macrophages and non-immune cells, will eventually be saturated and the LIP increases (Nairz and Weiss, 2020). Notably, HAMP was not only shown to downregulate FPN1, but also the iron-internalizing TFR1 and divalent metal transporter 1 in cultured astrocytes, brain microvascular endothelial cells and neurons. Indeed, HAMP overexpression or treatment, in the absence of pro-inflammatory cytokines, prevented both the uptake and release of transferrin-sequestered or free iron *in vitro* and iron translocation across the BBB *in vivo* (Du et al., 2011, 2015). As such, the main function of HAMP is to limit bidirectional iron transport in cells. Arguably, considering the presence of chronic neuroinflammation during the aging process as well as in AD and PD, HAMP, in combination with the production of other pro-inflammatory cytokines, may trigger cerebral iron accumulation. Therefore, it can be hypothesized that the anti-inflammatory effects of GLP-1 analogs (section “Inflammation”) might forestall the age- and neurodegeneration-associated iron deposition in the brain.

Indeed, both MCI patients and APOE4 carriers showed higher cortical iron load that was positively correlated with increased Aβ plaque load (van Bergen et al., 2016). Another study found correlations between the abnormally elevated levels of iron, senile plaques and Tau inclusions, with iron sequestered in plaques and microglia, in frontal and mid-cortical layers of AD patients (van Duijn et al., 2017). Furthermore, iron and Aβ co-pathology was associated with reduced neuropsychological test scores in individuals with MCI or AD (Ayton et al., 2017), while iron deposition in the frontal lobe was correlated with cognitive decline in AD (Damulina et al., 2020). Therefore, given that iron chelators, for example deferrioxamine, have seen some success for the treatment of AD (Guo et al., 2013, 2017) and PD animal models (Dexter et al., 1999) and patients (see i.e., Dusek et al., 2016; Ward et al., 2021), the metal ion hypothesis of AD has been proposed (Liu et al., 2018).

As summarized elsewhere, iron interacts with iron regulatory elements in APP mRNA to enhance the translation of APP, blocks furin expression to enhance BACE1 activity, interacts with γ-secretase to elevate Aβ generation, binds to Tau ad activates CDK5 and GSK-3β to induce Tau hyperphosphorylation and aggregation into NFTs (Liu et al., 2018). Of note, Aβ_1–42_ was demonstrated to bind, create and concentrate Fe_3_O_4_ within plaques, also known as iron oxide or magnetite (Tahirbegi et al., 2016). The latter has also been detected in polluted air and linked to dementia. However, while external magnetite was hypothesized to enter the brain via the olfactory bulb, it is not clear whether this is relevant and contributes to the magnetite that accumulates in the AD brain tissue (Maher et al., 2016; Plascencia-Villa et al., 2016; Chen H. et al., 2017). Interestingly, while Aβ monomers were shown to bind Fe_3_ in the region between Ser^8^ to Gly^25^, they could only do so when Fe_3_ was stabilized by an iron-chelator (Lermyte et al., 2019). In this context, Aβ_1–40_ and Aβ_1–42_ were shown to capture Fe_3_ from ferritin, whereby Aβ-bound Fe_3_ is readily converted into ROS-generating Fe^2+^ (Everett et al., 2014; Balejcikova et al., 2018). Other studies added that Aβ-Fe^2+^ interactions promote beta-sheet conformation and the self-polymerisation of Aβ monomers (Boopathi and Kolandaivel, 2016). Moreover, increased ratios of the Fe^2+^-oxidizing and detoxifying H-ferritin over the L-ferritin isoform were observed in the frontal cortex of AD and the caudate and putamen of PD patients (Connor et al., 1995; Mesquita et al., 2020). Thus, there appears to be a dynamic interaction between iron, the attempt to sequester iron by ferritin and the capture of ferritin-incorporated Fe_3_ by Aβ that triggers oxidative stress and plaque formation.

Besides other adverse mechanisms (see Lane et al., 2018), Fe^2+^ is known to produce ROS in the presence of H_2_O_2_, resulting in the generation of the neurotoxic membrane lipid peroxidation product 4-HNE that is prominent in the brains of AD, but also PD, patients (Kruman et al., 1997; Mattson and Pedersen, 1998; Di Domenico et al., 2017). Ultimately, intracellular iron overload leads to ferroptosis, a recently discovered form of cell death that is characterized by excessive iron-mediated ROS generation, lipid peroxidation and mitochondrial damage. Furthermore, ferroptosis was shown to be promoted by the downregulation of the anti-oxidative enzyme phospholipid glutathione peroxidase 4 (GPx4) (Sui et al., 2018; Sumneang et al., 2020).

There is evidence that GLP-1R activation prevents ferroptosis and oxidative stress. A recent study showed that iron deposition in hippocampal tissue as well as across the caudate nucleus, SN and putamen of T2DM patients have been correlated with cognitive decline (Yang Q. F. et al., 2018). In this context, the treatment db/db diabetic rodents with liraglutide, besides various synaptoprotective, neuroprotective and spatial memory-enhancing effects, rescued neurons and their mitochondria from ferroptosis, as indicated by the normalization of the increased serum and hippocampal CA1, CA3, and DG iron levels. The latter iron-decreasing effects of the GLP-1 analog involved raising the downregulated expression of the iron-storing H-ferritin and mitoferritin, lifting the decreased synthesis of the iron-exporting FPN1 and attenuating the elevated levels of the iron-uptake protein TFR1 (An et al., 2021). In addition, the GLP-1R agonist recovered the decreased expression of the ferroptosis-inhibitor GPx4, downregulated that of the lipid membrane re-modeling and ferroptosis-encouraging acyl-CoA synthetase long-chain family member 4 (Doll et al., 2017) and reverted the decreased transcription of SLC7A11, a cysteine-translocating protein that is necessary for GSH synthesis (An et al., 2021; Koppula et al., 2021). In agreement with the reduction in iron, liraglutide reduced the circulatory and hippocampal MDA and ROS generation, while increasing the levels of SOD2 and GPx, in db/db mice (An et al., 2021). Indeed, the anti-oxidative capabilities of GLP-1 (see section “GLP-1R-signalling protects from external oxidative stress, ROS production and the mitochondrial apoptosis pathway” and Figure 2) protected primary hippocampal neurons from Fe^2+^ or Aβ-triggered oxidative cell damage (Perry et al., 2003).

Notably, as an autoreactive catecholamine neurotransmitter and similar to H_2_O_2_, intra- and extraneuronal dopamine may generate massive amounts of ROS (^1^O_2_, O^2–^, and H_2_O_2_) by reacting with metal ions such as copper and iron, resulting in severe DNA oxidation and damage in dopaminergic neurons (Spencer et al., 2011). Generally, there is an age-dependent accumulation of iron in the basal ganglia, including the SN, putamen and globus pallidus (Callaghan et al., 2014). Beyond this age-related increase, although copper levels seem to be deceased, PD patients exhibit abnormally heightened iron deposition, lowered anti-oxidative GSH levels and decreases in the iron-chelator ferritin especially in the SN, but also related brain regions (Dexter et al., 1989, 1991; Riederer et al., 1989; Chen Q. et al., 2019; Mochizuki et al., 2020). It has been argued that the reduction in copper levels impairs the activity of iron-removing ferroxidases, thus augmenting the nigral amassment of iron, ROS production and tissue injury during PD (Montes et al., 2014). Indeed, the SN-microinjection of iron triggers parkinsonism in animals (Ben-Shachar and Youdim, 1991). Notably, post-mortem investigations showed that PD patients lose VMAT2 expression in the putamen, caudate and nucleus accumbens. VMAT2, whose expression is lost during PD, packages dopamine into presynaptic vesicles (Miller et al., 1999). This suggests that dopaminergic neurons are more vulnerable to the auto-oxidation of dopamine and concomitant DNA injury during PD. On the other hand, Exendin-4 was shown to preserve VMAT2-positive dopaminergic neurons in the SN of 6-OHDA-injected animals (Bertilsson et al., 2008), proposing that GLP-1 prevents the cytosolic accumulation of redox-reactive dopamine species (Figure 2).

### GLP-1 receptor induction prevents deoxyribonucleic acid damage and enhances deoxyribonucleic acid repair

Interestingly, GLP-1 analogs not only suppress oxidative stress to protect DNA integrity, but also elicit DNA repair mechanisms. Liraglutide and GLP-1R/GIPR dual agonists rescued cultured neurons from DNA fragmentation by H_2_O_2_ (Salles et al., 2018, 2020) or AGE-associated ROS production (An et al., 2015). Similarly, *in vivo*, the TUNEL assay indicated that GLP-1R agonist treatment reduced apoptosis-associated DNA fragmentation in the hippocampus or SN following the microinjection of kainate or MPTP, respectively (During et al., 2003; Zhang et al., 2015, 2020). Moreover, a study using menadione, a mitochondrial ETC inhibitor that enhances ROS production, showed that GLP-1R agonism in cortical neurons suppressed oxidative stress and evoked the CREB-mediated expression of apurinic/apyrimidinic endonuclease 1 (APE1), a member of the base excision DNA repair pathway, in an Akt (but not MEK_1/2_)-dependent manner (see Figure 2). Notably, APE1 expression was enhanced by GLP-1 or GLP-1 mimetics even in the absence of any stressors (Yang et al., 2016).

In this context, the transcription of APE1 was shown to be reduced in the entorhinal cortex and mononuclear blood cells of AD patients (Maynard et al., 2015; Lillenes et al., 2016), while the nuclear translocation of APE1 in the cerebral cortex was elevated in another study, as provoked by oxidative DNA damage in response to defective mitochondrial respiration and increased ROS production (Maynard et al., 2015). Likewise, by driving mitochondrial dysfunction and ROS/RNS-generation, Aβ_25–35_ attenuated APE1 levels *in vitro* (Kaur et al., 2015). In PD, genetic APE1 variants may be risk factors that accelerate the degeneration of dopamine neurons (Gencer et al., 2012), whereas the overexpression of APE1 counteracted the MPTP-induced ROS amassment and apoptosis of PC12 cells. The knockdown of APE1 accomplished the opposite result (Kang et al., 2017). APE1 further appears to be the only upregulated BER enzyme in response to glutamate-associated oxidative stress (Yang et al., 2010) as well as BDNF (Yang et al., 2014), while GLP-1-inducing agents were shown to raise the expression of BDNF in WT (Ohtake et al., 2014; Park et al., 2021), AD (Tai et al., 2018; Park et al., 2021) and PD-like animals (Ji et al., 2016; Lv et al., 2021) (see section “GLP-1 mimetics stimulate BDNF synthesis in neurons and glia”). As such, GLP-1R activation ameliorates oxidative stress-induced DNA damage by stimulating the CREB/APE1 axis in neurons in AD and PD.

### GLP-1 analogs induce PGC-1α to restore mitochondrial biogenesis in Alzheimer’s disease and Parkinson’s disease

Indeed, mitochondrial biogenesis, which is a process in which mitochondria adapt to greater energetic demands by growing in size and numbers, is disrupted during AD and PD. In this context, a key effector of the mitochondrial biogenesis is peroxisome proliferator-activated receptor gamma coactivator 1-alpha (PGC-1α). For instance, the decreased expression of PGC-1α has been detected in the SNpc of PD patients (Jiang et al., 2016), while AD patients displayed diminished CytC oxidase/complex IV levels, whose expression is under control of PGC-1α (Xu et al., 2014; Yang et al., 2017), in their post-mortem brain tissue and isolated platelets (Parker et al., 1990; Cardoso et al., 2004). A decline in PGC-1α and NRF-1/2 levels has also been observed in the hippocampus of APPswe/PS1dE9 mice (Pedros et al., 2014). PGC-1α is strongly expressed in energy-demanding tissues, including dopaminergic neurons in the brain (Corona and Duchen, 2015). Besides regulating the mitochondrial biogenesis, given that PGC-1α further co-induces other mitochondrial effectors [NRF-1/2 and transcription factor A, mitochondrial (TFAM)], modulates anti-oxidant gene expression (SOD, catalase, GSH), prevents mitochondrial dysfunction and blocks α-synuclein oligomerisation and apoptosis, PGC-1α plays a key role especially in PD (Ebrahim et al., 2010; Shin et al., 2011; Mudo et al., 2012; Corona and Duchen, 2015; Ye et al., 2017).

As confirmed in SH-SY5Y cells stressed with the PD toxin 6-OHDA, the application of a GLP-1R/GIPR dual agonist reverted the downregulation of PGC-1α and NRF-1 (Zhang L.Y. et al., 2021). Similarly, Liraglutide blocked H_2_O_2_-provoked mitochondrial ROS production, membrane potential loss and structural damage, while potentiating the expression of PGC-1α to raise the number of mitochondria in RGC-5 cells (Ma et al., 2017). These biogenic effects were replicated *in vivo*, where a GLP-1R/GIPR dual agonist normalized the quantities and volume of neuronal mitochondria in the brains of 3 × Tg (APP/PS1/Tau) AD mice (Cai et al., 2021).

Notably, in contrast to Akt/mTor-activating hormones that are associated with growth and nutrient abundance, such as insulin or GLP-1, conditions of caloric restriction and the release of Ghrelin induce the nutrient sensor AMPK to allow neurons to adapt to the lack of bioenergetic substrates (glucose). This involves the AMPK-mediated shut down of Akt/mTor-driven cell proliferation, growth and protein translation, the stimulation of PGC-1α/NRF-1/2-induced mitochondrial gene transcription and a switch toward lipid β-oxidation (see Reich and Holscher, 2020 for details).

Nonetheless, insulin resistance was found to impair the expression of PGC-1α and NRF-1 in T2DM patients (Patti et al., 2003). There seems to be a reciprocal relationship, with impeded PGC-1α activity triggering insulin resistance. In turn, IR knockout experiments and the use of insulin-re-sensitizing agents confirm that the resolution of insulin resistance restores the impaired activity of PGC-1α, energy metabolism and mitochondrial biogenesis (Pagel-Langenickel et al., 2008). In this context, CREB/CREB-regulated transcription coactivator 2 (CRTC2)-co-signaling drives the expression of PGC-1α. It appears that insulin resistance heightens the activity of salt inducible kinases (SIKs) that phosphorylate CRTCs, leading to their sequestration by 14-3-3 proteins in the cytoplasm. Hence, this diminishes the nuclear translocation of CTRCs and the expression of PGC-1α under insulin-resistant conditions. GLP-1, in turn, not only improves insulin sensitivity, but also induces cAMP/PKA-signaling to inhibit SIKs and directly activate CREB to restore the CREB/CRTC2-driven transcription of PGC-1α (Hogan et al., 2015; Rahnert et al., 2016; Wein et al., 2018; Hölscher, 2020). Indeed, in the sporadic UCD-T2DM rat model, the development of peripheral insulin resistance was associated with the hippocampal desensitization of the insulin pathway, resulting in enhanced lipid peroxidation (4-HNE) levels that were inversely correlated with those of PGC-1α. Liraglutide reversed these abnormal changes in the brain and further restored the levels of TFAM and other metabolic energy markers (Agrawal et al., 2014). Since cerebral insulin resistance is an early pathological event during AD and PD, while GLP-1 mimetics were shown to re-sensitize insulin-signaling in the brain (section “Insulin resistance and the neuronal energy metabolism”), this represents another mechanism that preserves the mitochondrial function during neurodegenerative diseases.

### GLP-1 receptor agonists suppress amyloid beta and GSK-3β-driven mitochondrial damage in Alzheimer’s disease

Similar to PD, mitochondrial dysfunction is an early pathologic event in AD and promoted by Aβ. Indeed, mitochondrial abnormalities have been well documented in the brains of AD patients (Silva et al., 2012) and reviewed elsewhere (see Chaturvedi and Flint Beal, 2013). Aβ, which may enter mitochondria across cyclophilin D-containing mitochondrial permeability transition pores (miPTPs) or import by the translocase of the outer membrane (TOM) complex (Hansson Petersen et al., 2008; John and Reddy, 2021), contributes to mitochondrial injury by (i) the accumulation of APP/Aβ in the inner-mitochondrial membrane and import channels, which impairs oxidative phosphorylation (OXPHOS) and ATP generation, disrupts the mitochondrial membrane potential (MiMP) and increases ROS generation, (ii) the ROS-enhancing interaction of Aβ with Aβ-binding alcohol dehydrogenase (AβAD) within mitochondria and (iii) the binding of Aβ to cyclophilin D that evokes the opening of miPTPs, leading to, as further accelerated by oxidative stress or cytosolic Ca^2+^ overload, oxidative stress-associated mtDNA, lipid and protein damage, Ca^2+^ amassment in the mitochondrial matrix, swelling, depolarisation of the mitochondrial membrane potential, mitochondrial leakage and neuronal death (Mammucari et al., 2018; Perez Ortiz and Swerdlow, 2019). Aβ was further shown to impair the activities of complex I (in conjunction with Tau), complex III and, especially, complex IV of the ETC as well as the TCA enzymes α-ketoglutarate dehydrogenase (α-KGDH) and PDH (Casley et al., 2002; Caspersen et al., 2005; Rhein et al., 2009). In turn, an impaired ETC heightens APP/Aβ processing (Mammucari et al., 2018; Perez Ortiz and Swerdlow, 2019). Other harmful events include that Aβ interferes with the anterograde axonal transport of mitochondria toward synapses, thus accelerating synaptic degeneration (Calkins and Reddy, 2011), and drives mitochondrial depletion and fragmentation due to the Aβ-induced deregulation of fusion and fission proteins (Wang et al., 2008, 2009). Many of the detrimental effects of Aβ are linked to the activation of GSK-3β, as shown in Figure 2 and explained below.

Besides protecting from oxidative stress-associated mitochondrial damage, as discussed in the previous sections, GLP-1R induction shields mitochondria against Aβ and the associated harmful activation of GSK-3β. An *ex vivo* study demonstrated that the administration of GLP-1 protected mitochondria from Aβ_1–42_, as indicated by lessened mitochondrial ROS generation, in hippocampal slices. *In vivo*, GLP-1 further rescued the abnormally lowered Akt and heightened GSK-3β activities in the hippocampus of APP/PS1 rodents (Ma et al., 2012). Notably, the latter normalizing effects on Akt and GSK-3β, as related to the re-sensitization of the insulin pathway in the brain, were replicated by several GLP-1R or GLP-1R/GIPR dual agonists in different AD cell (Jantrapirom et al., 2020) and animal models (Chen et al., 2012; Ma et al., 2012; Cai et al., 2014; Qi et al., 2016; Cao et al., 2018; Wang et al., 2018; Zhou et al., 2019; Paladugu et al., 2021).

The pathologic induction of GSK-3β is thought to be a key event in AD. The activation of GSK-3β, as driven by the Aβ-provoked impairment of insulin-signaling and the IR/PI3K/Akt pathway that suppress GSK-3β, negatively affects the mitochondrial biogenesis, motility, bioenergetics, integrity and mitochondria-associated apoptosis pathways (Takashima, 2006; Yang et al., 2017). There are four mechanisms with which Aβ-associated GSK-3β-signaling injures mitochondria and all of them are resisted with incretin hormone treatment (Figure 2).

First, GSK-3β opposes the insulin pathway to trigger reductions in the levels of PGC-1α, impairments in the mitochondrial biogenesis and mitochondrial fragmentation. PGC-1α is responsible for the transcription of key biogenesis genes, such as ETC enzymes (subunits of CytC oxidase/complex IV or the ATP5B subunit of ATP synthase/complex V), ANT1 (an ADP/ATP translocation channel), CytC (implicated in e^–^ carriage and ATP synthesis by the ETC), NRF-1α (anti-oxidative transcription factor) and TFAM (mtDNA regulator and replication-inducer) (Xu et al., 2014; Yang et al., 2017). The GSK-3β mediated degradation of PGC-1α seems to involve GSK-3β cleavage by Omi, which, in a GSK-3β-dependent manner, enhances the interaction of PGC-1α with SCF-Cdc4 E3 ligase to ubiquitinate and target PGC-1α for proteasomal degradation (Xu et al., 2014). On the other hand, GSK-3β (and p38) drive mitochondrial fragmentation by upregulating the fission-enhancers DLP1 and mitochondrial fission factor (MFF) levels and decreasing those of the fusion-modulator Mfn2 (Yang et al., 2017). In this context, insulin-mediated PI3K/Akt-signaling regulates PGC-1α and mitochondrial biogenesis through the Akt-driven inhibition of GSK-3β, but also FOXO1 and PGC-1α. Furthermore, insulin was shown to augment the expression of important mitochondrial modulators, including Mfn2, TFAM or a subunit of CytC oxidase/complex IV (Litwiniuk et al., 2016). Of note, even though FOXO1 is responsible for the transcription of PGC-1α, FOXO1 may impede the activity of PGC-1α (Yang et al., 2017).

There is evidence that GLP-1 mimetics restore the PGC-1α-driven mitochondrial biogenesis and rescue mitochondrial hyperfission in response to the activation of Aβ/GSK-3β and oxidative stress. A recent study showed that the application of GLP-1 agonists blocked the induction of GSK-3β and enhanced the transcription of PGC-1α, NRF-1, and TFAM to heighten the expression of the anti-oxidative enzymes catalase, SOD2 and GPx and prevent Tau hyperphosphorylation in AGE-injected animals. Moreover, this led to reduced mitochondrial ROS production, cristae damage and vacuole formation following AGE-treatment *in vitro* (An et al., 2015). In this context, besides enhancing mitochondrial biogenesis, PGC-1α further buffers the mitochondrial ROS production by navigating the expression of the aforementioned anti-oxidative enzymes (Rius-Perez et al., 2020). Interestingly, AGE-induced oxidative stress interrupted the interaction of PGC-1α with GSK-3β, whereas GLP-1 agonists restored protein binding. It is likely that the Akt-mediated (and inactivating) Ser^9^-phosphorylation of GSK-3β evokes this interaction with PGC-1α, although further studies are needed to decipher the downstream effect (An et al., 2015).

Notably, the cAMP/PKA pathway protects from mitochondrial fragmentation though the PKA-driven phosphorylation and inhibition of the fission protein DLP-1 at Ser^637^ (Chang and Blackstone, 2007; Monterisi et al., 2017). Both Liraglutide and Exendin-4 prevented the decrease in cAMP, phospho-PKA and phospho- Ser^637^-DLP-1 levels and restored those of the mitochondrial fusion inducers Mfn2 and OPA1 in the cortex of 5xFAD mice (An et al., 2019; Xie et al., 2021), which suppressed ROS overproduction, raised ATP generation and rescued cortical and hippocampal neuronal loss (Xie et al., 2021). The latter effects, including the cAMP/PKA-dependent inhibition of DLP-1, increase in fusion proteins as well as the protection from mitochondrial fragmentation, dysfunction and cell death were replicated in GLP-1 and Aβ_1–42_-co-treated cortical astrocytes *in vitro* (Xie et al., 2021). Indeed, the application of the GLP-1/GIP dual agonist DA4-JC enhanced the mitochondrial size and reduced mitochondria numbers in the hippocampus of 3 × Tg mice, confirming that GLP-1R activation protects from Aβ/GSK-3β-provoked mitochondrial fragmentation (Cai et al., 2021).

To connect the improvements in PGC-1α and mitochondrial fission/fusion, the GLP-1R-mediated neuroprotection in AD animal models was shown to involve the PKA-mediated phosphorylation and activation of CREB (Cai et al., 2018; Li et al., 2020a). The latter transcription factor is jointly induced by the GLP-1R-mediated stimulation of the PI3K/Akt, Ras/Raf/MEK/ERK and cAMP/PKA pathways. Moreover, as dependent on CREB, Exendin-4 protected from caspase 3-driven apoptosis by the mitochondrial pathway in Aβ oligomer-stressed human neuroprogenitor cells (Velmurugan et al., 2012). In the context of mitochondria, a study showed that the enhancement of cAMP/PKA-signaling restored phospho-CREB and PGC-1α levels as well as mitochondrial defects in APP_*swe*_ M17 cells (Sheng et al., 2012). Indeed, ATF2 and CREB jointly induce the transcription of the mitochondrial biogenesis-inducer PGC-1α (Fernandez-Marcos and Auwerx, 2011), while PGC-1α enhances the transcription of Mfn2 and downregulates the expression of DLP-1 and phospho- Ser^637^-DLP-1 levels (Peng et al., 2017). Multiple studies further confirm that GLP-1R-induced cAMP/PKA-signaling is necessary for the protection from Aβ and mitochondrial damage in the brains of AD and T2DM rodents (Batista et al., 2018; Candeias et al., 2018; Xie et al., 2021).

Synoptically, GLP-1R agonists restore the neuronal insulin sensitivity and induce Akt to suppress the GSK-3β-mediated degradation of PGC-1α, oxidative stress and mitochondrial fragmentation following Aβ-exposure. Moreover, GLP-1R activation drives cAMP/PKA-signaling and the activation of CREB to normalize the expression of PGC-1α and fission/fusion-modulating proteins.

Second, the induction of GSK-3β elicits the oxidative stress-promoted phosphorylation of the miPTP components cyclophilin D and voltage-dependent anion channel 2 to provoke excessive Ca^2+^-influx, swelling and mitochondrial membrane potential collapse (Yang et al., 2017). Indeed, the Akt pathway and inhibitory Ser^9^ phosphorylation of GSK-3β were shown to prevent miPTP opening and mitochondrial swelling in response to excessive ROS production (Juhaszova et al., 2004). GSK-3β further counteracts Akt/NRF-2-mediated anti-oxidative gene transcription (such as HO-1 Zhao et al., 2021c), elicits the joint inactivation of the key TCA enzyme PDH with TPKI and inhibits mitochondrial complex I-IV and ATP synthesis. The impaired mitochondrial function, in turn, exacerbates ROS generation (see Yang et al., 2017; Figure 2).

In an Aβ_1–42_-induced AD rat model, Exendin-4 engaged PI3K/Akt-signaling to re-invigorate the impaired Akt phosphorylation, mitochondrial function, integrity, respiratory control ratio and ADP phosphorylation, while normalizing mitochondrial complex I, IV, and V activities (which were aberrantly enhanced by the acute Aβ challenge in this study, however) (Garabadu and Verma, 2019). Similarly, Exendin-4 rescued the reduced complex I expression, ATP production and oxidative stress, as indicated by lowered SOD and increased MDA levels, in the hippocampus of 5xFAD rodents (An et al., 2019). Indeed, the mitochondrial enhancements by Exendin-4 correlated with spatial and working memory improvements in i.c.v. Aβ_1–42_-injected rodents (Garabadu and Verma, 2019). Besides Aβ, the latter GLP-1 mimetic blocked cell death, restored the mitochondrial biogenesis and the synthesis of mitochondrial ETC-related enzymes (complex I/NADH dehydrogenase 1, complex II/succinate dehydrogenase, complex III/cytochrome b 6, complex V/ATPase 6) via the PI3K/Akt pathway in INS-1E cells that were stressed with human islet amyloid polypeptide, which is a pancreatic amyloid (Fan et al., 2010).

Transmission electron microscopy further confirmed that GLP-1 agonists protect from Aβ_1–42_-induced swelling, the loss of surface area and cristae as well as the deformation of mitochondria in hippocampal neurons *in vivo* (Qi et al., 2016; An et al., 2019). Importantly, mitochondrial cristae are sites for ETC complex formation. OPA1 remodels cristae by fusing parts of the inner-mitochondrial membrane (Baker et al., 2019), whereas Aβ predominantly accumulates in cristae (Hansson Petersen et al., 2008) and downregulates OPA1 levels (An et al., 2019; Xie et al., 2021). Notably, the insulin pathway regulates APP and Aβ trafficking across the trans-Golgi network to the plasma membrane through the MEK/ERK pathway, thus preventing the intraneuronal amassment of Aβ (Gasparini et al., 2001). As such, the re-sensitization of the insulin pathway in neurons by GLP-1 mimetics (section “Insulin resistance and the neuronal energy metabolism” and Figure 1) is likely to forestall Aβ accumulation and mitochondrial damage.

Third, GSK-3β drives cell death by the mitochondrial apoptosis pathway through the direct inactivation of the anti-apoptotic Mcl-1 and activation of Bax, p53 and p21*^Cip1^*. Moreover, the activity of GSK-3β correlated with that of caspase 2/8, subsequent Bid cleavage and CytC liberation (Maurer et al., 2006; Nie et al., 2015; Yang et al., 2017). On the other hand, the pharmacological inhibition of GSK-3β rescued caspase 3-induced apoptosis in SH-SY5Y cells following treatment with the mitochondrial complex I inhibitors MPTP and rotenone (King et al., 2008).

As indicative of the suppression of the mitochondrial apoptosis pathway, the administration of GLP-1 analogs or GLP-1R/GIPR dual agonists enhanced the anti-apoptotic Bcl-2 levels in the hippocampus of WT, 3 × Tg, and 5xFAD rodents (Park et al., 2021), normalized the increased Bax/Bcl-2 ratio in rats with hippocampal STZ-injections (Shi et al., 2017; Li et al., 2020a) and lowered caspase 3 protein levels in the hippocampus of APP/PS1 rodents (Holubova et al., 2019). In this context, the activation of the GLP-1R/PI3K/Akt leads to the Akt/CREB-conveyed transcriptional upregulation of Bcl-2 (Pugazhenthi et al., 2000) as well as the Akt-driven direct inactivation of GSK-3β and other pro-death effectors, such as the Bcl-2-sequestering Bad, pro-caspase 9 upstream of caspase 3, Bax and p53 (as part of the JNK/p53/Bax death axis) and FOXO transcription factors, for instance the FasL-synthesizing FOXO1 or the mTORC1-inhibitor FOXO3a (Kaplan and Miller, 2000; Garza-Lombo et al., 2018; Rai et al., 2019; Hölscher, 2020).

Fourth, as rescued by GLP-1 mimetics, GSK-3β is implicated in cholinergic dysfunction. As reversible with GSK-3β inhibitors, cellular stress elicits the detrimental mitochondrial translocation of GSK-3β to interfere with the mitochondrial biogenesis, dynamics, energy metabolism, mitochondrial membrane integrity, and survival pathways (Yang et al., 2017). As one of these stressors, Aβ_1–42_ provokes the activation of GSK-3β in mitochondria, which led to the GSK-3β-mediated inhibition of PDH and, hence, lowered levels of the acetylcholine-precursor acetyl-CoA in a study (Hoshi et al., 1997). In turn, Exendin-4 restored cholinergic dysfunction by engaging the GSK-3β-inactivating PI3K/Akt pathway, resulting in the normalization of the attenuated acetylcholine levels, lowered choline acetyltransferase and heightened acetylcholinesterase activities in the prefrontal cortex and hippocampus of i.c.v. Aβ_1–42_ infused rats (Garabadu and Verma, 2019).

## Insulin resistance and the neuronal energy metabolism

### GLP-1 mimetics upregulate the expression of mitochondrial bioenergetic enzymes

As master regulator of the cellular energy metabolism, the mTor pathway is responsible for the cellular growth and survival, protein translation, redox balance, and autophagy. Upon activation of growth factor receptors, including insulin and GLP-1, the induction of PI3K/Akt-signaling leads to the inactivation of the Rheb/mTORC1-repressor TSC_1/2_. Once activated, mTORC1 phosphorylates S6K and inactivates 4E-BP. This leads to increased protein translation, the mTORC1/4E-BP-specific induction of glycolytic genes via HIF1α, mTORC1/S6K/sterol regulatory element-binding protein (SREBP_1/2_)-mediated cell proliferation, lipid and nucleotide biosynthesis as well as the induction of the aerobic arm of the pentose phosphate pathway (PPP) that neurons utilize to generate NADPH for oxidative protection. Indeed, the induction of the Akt/mTor axis enhances the synthesis of glycolytic enzymes, for example HK2, PKM2, or LDH, as well as related effectors, such as GLUT1, and PPP enzymes, i.e., 6-phosphofructo-2-kinase/fructose-2,6-biphosphatase 3 (PFKFB3) (Duvel et al., 2010; Garza-Lombo et al., 2018; Hölscher, 2020; Liu et al., 2021). In addition, mTORC1, by inhibiting 4E-BP, enhances the translation of nuclear, mitochondria-associated mRNAs, for example those for TFAM that augment the replication of mtDNA and transcription of ATP synthase (mitochondrial complex 5) of the ETC. Lastly, the mTORC1/yin-yang 1 (YY1)-driven induction of PGC-1α is mandatory to sustain ATP production via oxidative phosphorylation (OXPHOS) and the mitochondrial biogenesis (Cunningham et al., 2007; Blattler et al., 2012; Morita et al., 2013).

An *in vitro* study indicated that the treatment with GLP-1 agonists upregulates the expression of pyruvate dehydrogenase even in unstressed SH-SY5Y cells (Jalewa et al., 2016), suggesting that GLP-1R-signaling directly supports the expression of glycolytic enzymes and, hence, the neuronal energy metabolism. Besides directly upregulating the activity of the mTor-inducer Akt in AD (Chen et al., 2012; Ma et al., 2012; Cai et al., 2014; Qi et al., 2016; Cao et al., 2018; Wang et al., 2018; Zhou et al., 2019; Paladugu et al., 2021) and PD (Ji et al., 2016; Jalewa et al., 2017) animal models, GLP-1 re-invigorated the impaired IR/PI3K/Akt/mTor insulin pathway, as impeded by inflammation and Aβ, in neurons (Figure 1 and details in section “GLP-1R activation re-sensitises insulin signaling in the brain during AD and PD”). As a proof of concept, a phase II clinical trial in PD patients demonstrated that Exendin-4 enhanced insulin sensitivity, Akt and phospho-mTor levels in the brains of PD patients (Athauda et al., 2019). Furthermore, liraglutide prevented a decline in the cerebral glucose metabolization and BBB glucose transfer rates in AD patients (Gejl et al., 2016, 2017).

### GLP-1 receptor activation re-sensitizes insulin signaling in the brain during Alzheimer’s disease and Parkinson’s disease

While ignored in light of the amyloid cascade hypothesis in AD over the last decades, cerebral insulin resistance has now been acknowledged as an independent and early pathologic key event in both AD and PD. Multiple studies have demonstrated that AD patients exhibit pronounced deficits in the neuronal glucose metabolism and blood flow velocities in memory and cognition-processing brain regions, for example the temporoparietal and posterior cingulate cortex, correlating with the clinical progression of AD (Lying-Tunell et al., 1981; Hoyer et al., 1988; Ogawa et al., 1994; Drzezga et al., 2003; Mosconi et al., 2008). Worsened blood flow and glucose hypometabolism, were also identified in the brains of PD patients which, interestingly, not only correlated to PD severity, but also the occurrence of dementia during PD (Huang et al., 2008; Hosokai et al., 2009; Liepelt et al., 2009; Xu Y. et al., 2015). Moreover, both AD and PD patients showed the impaired expression of glycolytic and TCA-associated enzymes, while the formation of protein and lipid oxidation products was enhanced, symbolizing defects in the neuronal energy metabolism and elevated ROS-production in response to the bioenergetic dysfunction (Iwangoff et al., 1980; Dunn et al., 2014; Zhao et al., 2015). The latter energetic impairments are believed to be an early consequence of insulin resistance in the brain, proposed to precede mitochondrial dysfunction and amyloid pathology. Importantly, it has been hypothesized that neuronal stress initially elicits glucose hypermetabolism in AD, followed by the development of cerebral insulin resistance and associated impairments in glucose metabolism that enforce a detrimental shift from glucose to alternative, but less efficient, energy substrates, for example ketone bodies and lactate (Neth and Craft, 2017; Zilberter and Zilberter, 2017; Holscher, 2019). Indeed, markers of insulin resistance, such as increased quantities of Ser-phosphorylated and inhibited IRS-1 downstream of the IR, have been identified in the hippocampus and cortex of AD patients, correlating with plaque deposition, disease progression and memory decline (Steen et al., 2005; Moloney et al., 2010; Talbot et al., 2012). Likewise, PD patients displayed premature attenuations in IR receptor synthesis and insulin pathway inactivation in the SNpc, basal ganglia and other brain areas (Moroo et al., 1994; Takahashi et al., 1996; Tong et al., 2009; Morris et al., 2014).

Given that T2DM is a well-established AD and PD risk factor (Hölscher, 2020), cerebral insulin resistance is considered to be an important pathological event in neurodegenerative diseases. As a pivotal growth factor in the brain, the detrimental consequences of desensitized IR-signaling [IRS-1/PI3K/Akt/mTor and MAPK (ERK) pathways] result in synaptic, plasticity and memory deficits, accelerated plaque deposition, GSK-3β-mediated Tau hyperphosphorylation and aggregation, cellular growth, protein synthesis, mitochondrial and autophagy impairments, reduced protection from oxidative stress, diminished neuronal GLUT4 translocation (as regulated by the insulin pathway and inversely correlating with insulin resistance) and astroglia GLUT1 expression (which co-localizes with insulin resistance-associated Tau pathology), lowered cerebral glucose uptake, decreased blood flow due to the endothelial disturbance of insulin/NO-mediated vasoconstriction and, of course, bioenergetic defects due to the loss of glycolytic enzyme expression (Craft, 2009; Mullins et al., 2017; Neth and Craft, 2017; Holscher, 2019; Akhtar and Sah, 2020). Both AD patients and 3 × Tg AD mice displayed early peripheral glucose intolerance that was followed by attenuated PI3K/Akt-signaling, glycolytic flux, neuronal GLUT3 levels (but not astroglia GLUT1) and GLUT3 translocation to the plasma membrane as well as elevated glucose levels in the brain, suggesting a link between peripheral and cerebral insulin resistance (An et al., 2018; Griffith et al., 2019). Notably, CREB regulates the expression of GLUT3, which is the predominant neuronal glucose transporter in the CNS (Jin et al., 2013). The cAMP/PKA/CREB pathway was shown to be impaired in the prefrontal cortex or hippocampus of AD patients (Yamamoto-Sasaki et al., 1999; Liang et al., 2007; Bartolotti et al., 2016), while the Aβ/Ca^2+^/calpain-mediated degradation of CREB, oxidative stress and astrogliosis reduce CREB levels, lead to CREB truncation and, as such, decrease the expression of GLUT3 during AD (Puzzo et al., 2005; Pugazhenthi et al., 2011; Jin et al., 2013). Indeed, a study showed that the PKA-enhancer forskolin elevated the CREB-driven GLUT3 expression, glucose uptake and intracellular protein *O*-GlcNAcylation in SH-SY5Y cells (Jin et al., 2013).

The latter studies suggest that the insulin resistance-associated reduction in the cerebrovascular blood flow and astroglia GLUT1 expression reduce the BBB translocation of glucose, while the neuronal impairment in the PI3K/Akt pathway disturbs the exocytosis of GLUT3 and promotes the extraneuronal accumulation of glucose in the brain. Moreover, defects in PI3K/Akt/mTor-signaling interfere with the global protein translation and glycolytic energy metabolism in neurons. On the other hand, as a secondary consequence of reduced growth factor signaling, pathology-associated stress, including Aβ-induced Ca^2+^ overload, oxidative stress and astrogliosis, disturb the neuronal CREB activity to reduce GLUT3 expression and exacerbate glucose amassment in the brain tissue.

Moreover, the IR contributes to declarative memory consolidation. Mice haploinsufficient for an allele of the IR β-subunit displayed selective deficits in PI3K/Akt/mTor-dependent, translational and long-lasting forms of LTP, which led to impairments in the hippocampal consolidation of object recognition memory (Nistico et al., 2012). Indeed, memory-enhancing effects were observed following intranasal treatment in healthy volunteers (Benedict et al., 2004), older adults (Reger et al., 2006) as well as early AD patients that presumably had not developed cerebral insulin resistance yet (Craft et al., 2012).

Although not a focus of this review, there is plentiful evidence that GLP-1 agonists protect from cerebral and peripheral insulin resistance as well as T2DM-associated hippocampal damage *in vivo* (see Gault and Holscher, 2018). First, besides T2DM-associated animals, GLP-1 mimetics were shown to ameliorate markers of peripheral insulin resistance, including abnormally raised plasma insulin (Li et al., 2010b), circulatory glucose (Li et al., 2010b; Holubova et al., 2019) and glycated hemoglobin A1c levels (Li et al., 2010b), which is a clinical marker for T2DM that reflects the plasma glucose levels over the last 3 months (Sequeira and Poppitt, 2017), in AD animal models. Importantly, the chronic application of GLP-1 mimetics or GLP-1R/GIPR agonists do not affect insulin sensitivity, circulatory or hippocampal insulin, blood glucagon and leptin as well as fasting nor brain glucose pools in animals without metabolic impairment, although the anorexic effects of GLP-1 may promote mild weight loss (Chen et al., 2012; McGovern et al., 2012; Bomba et al., 2013; Lourenco et al., 2013; McClean et al., 2015; Qi et al., 2016; Chen S. et al., 2017; Yuan et al., 2017; Feng et al., 2018; Panagaki et al., 2018; Holubova et al., 2019; Zhou et al., 2019; Cai et al., 2021; Park et al., 2021). Second, in T2DM animal models, GLP-1R activation further resolved the cerebral insulin resistance, leading to the reversal of PI3K/Akt-signaling impairments, GSK-3β overactivation and concomitant Tau hyperphosphorylation (Yang et al., 2013; Ma et al., 2015; Spolcova et al., 2015; Xu W. et al., 2015), oxidative stress (heightened MDA and diminished SOD activities), apoptosis mediator induction (decreased Bcl-2/Bax ratios and Bcl-2 expression, with elevated Bax and active caspase 3), interrupted autophagy [deregulated AMPK/mTor pathway with lessened microtubule-associated protein light chain 3 (LC3)-II-containing autophagosomes and Beclin-1 plus exaggerated p62 levels], synaptic injury, neuronal atrophy as well as learning and memory deficits (Kong et al., 2018; Yang Y. et al., 2018; Yan et al., 2019).

Importantly, GLP-1 agonists have shown insulin-re-sensitizing effects in the brains of AD animal models. For example, GLP-1 agonists prevented the loss of dendritic IRs *in vitro* and insulin receptors (IR) in the prefrontal cortex and/or hippocampus of rodents and non-human primates *in vivo* following exposure or infusion of Aβ-oligomers (Batista et al., 2018). Furthermore, neurons in the frontal cortex of APP/PS1 exhibited aberrantly distributed IRs, forming dense receptor bundles in the neuropil, as well as increased levels of IRS-1 with inhibitory Ser^616^-phosphorylation. The latter indications of cerebral insulin resistance were not observed in age-matched control mice and co-localized with plaques and plaque-attracted glial cells. Treatment with liraglutide, however, ameliorated the plaque burden, diminished the microglial and astroglia activation, reduced plaque-interaction with glial cells and, most importantly, decreased the levels of inactivated phospho-Ser^616^-IRS-1 as well as IR inclusions (Long-Smith et al., 2013). Similarly, the GLP-1/GIP dual incretin DA-JC4 reverted the elevated phopsho-Ser^1101^-IRS-1 and diminished phospho-Akt levels in the cortex and hippocampus of the i.c.v. STZ-injected AD rodent model (Shi et al., 2017), while liraglutide elevated the levels of phosphorylated IRs in cortical areas of WT and 5xFAD mice (Paladugu et al., 2021).

There are three mechanisms with which GLP-1R induction protects from cerebral insulin resistance, as illustrated in Figure 1. First, GLP-1R-signaling forestalls Aβ production, accumulation and secretion by neurons through the inhibition of BACE1, the upregulation of IDE and normalization of the impaired autophagy function that is responsible for amyloid removal (section “GLP-1R agonists are neuroprotective and prevent amyloid beta accumulation *in vivo*”).

Second, previous studies indicate that Aβ-oligomers bind to hippocampal synapses, provoke Ca^2+^-influx, the activation of Ca^2+^-sensitive enzymes calcium calmodulin-dependent kinase II plus casein kinase II, and the weakening of insulin-signaling by eliciting the internalization of membrane IRs, in particular those on dendrites. These effects could be replicated with glutamate or K^+^-evoked neuronal depolarisation and Ca^2+^-influx, while the Aβ-mediated endocytosis of IRs could be prevented with NMDAR antagonists or a Ca^2+^-chelator (Zhao et al., 2008, 2009; De Felice et al., 2009; Chan et al., 2016). Furthermore, the APOE4 allele, but also a high fat diet, accelerate the Aβ_1–42_-enforced trapping of IRs in endosomes in hippocampal neurons, which impaired insulin-signaling, the mitochondrial function and glycolysis, AMPAR-GluR1 phosphorylation and synaptic insertion, postsynaptic plasticity and spatial memory (Chan et al., 2016; Zhao et al., 2017). In turn, oxidative stress, the loss of IRs and synaptic spines as well as LTP impairments could be rescued with insulin or insulin-re-sensitizer treatment in an IR-dependent-manner (Zhao et al., 2008, 2009; De Felice et al., 2009; Lee et al., 2009; Chan et al., 2016). Synoptically, Aβ evokes IR internalization by stimulating aberrant Ca^2+^ accumulation in the cytosol, whereas insulin/IR-signaling seemingly reduces the synaptic binding of oligomeric Aβ by preserving the synaptic exposition of α-amino-3-hydroxy-5-methyl-4-isoxazolepropionic acid receptors (AMPARs; steric hindrance) and through mediating the endocytosis of Aβ oligomers for the intracellular reduction to Aβ monomers and degradation via IDE (Zhao et al., 2008, 2009; De Felice et al., 2009; Chan et al., 2016).

Importantly, GLP-1R agonists protect from the Aβ/Ca^2+^-induced IR defects in neurons. Notably, a cAMP-inducer rescued insulin-signaling in ApoE3xAPP mouse-derived hippocampal slices (Zhao et al., 2017), while the induction of GPCRs, in a cAMP/PKA-dependent manner, was shown to prevent the K^+^-triggered excessive Ca^2+^ influx through L-VDCCs (Cheng et al., 1998). In this context, GLP-1R-driven cAMP/PKA-signaling and CREB gene induction protect from the Aβ, K^+^ ion or excitotoxicity-enforced opening of NMDARs and VDCCs which, besides preventing the depletion of ER Ca^2+^ stores and Ca^2+^ overload (section “GLP-1 mimetics suppress Ca2+ deregulation by amyloid beta and excitotoxicity”) (Gilman et al., 2003; Qin et al., 2008; Cai et al., 2017). Notably, unlike IRs, GLP-1Rs were not downregulated by oligomeric Aβ *in vitro*/*in vivo* (Bomfim et al., 2012). Moreover, Exendin-4, in contrast to insulin, did not prevent the interaction of soluble Aβ aggregates with neurons (Bomfim et al., 2012), albeit liraglutide reduced the interaction of oligomeric Aβ with synapses in another study (Batista et al., 2018). This suggests that (i) the GLP-1 pathway does not seem to desensitize in response to the Aβ pathology or in general [which is also why GLP-1, but not GIP, analogs were chosen as clinical T2DM treatments (Gault and Holscher, 2018)], (ii) GLP-1 mimetics prevent IR endocytosis through the GLP-1R-mediated suppression of the Ca^2+^ overload that is provoked by Aβ oligomers and (iii) GLP-1 may prevent the harmful interaction of soluble and extracellular Aβ species with neurons, possibly by promoting insulin-signaling (Bomfim et al., 2012). As discussed elsewhere, GLP-1R stimulation also aids the removal of Aβ (section “GLP-1R agonists are neuroprotective and prevent amyloid beta accumulation *in vivo*”).

Third, while insulin exerts anti-inflammatory effects, inflammation is also the primary cause of insulin resistance in the brain (see Figure 1; Holscher, 2019). For example, the i.c.v-injection of LPS caused memory impairments and desensitized insulin-signaling in the hippocampus of rats, as implied by Ser^307^-phosphorylated IRS-1 and decreased phospho-Akt levels, whereas insulin co-treatment rescued the latter (Iloun et al., 2018). Mechanistically, similar to what happens in the periphery during T2DM, the TLR/NF-κB-mediated production and release of pro-inflammatory cytokines by stimulated microglia and astrocytes, including IL-1β, IL-6, IL-18, and TNF-α, result in PICR activation on neurons during AD and PD. Neuronal PICR induction leads to the MKK-mediated induction of JNK and p38, which both enhance the AP-1-driven transcription of APP, as well as the activation of IKKβ, resulting in IKKβ/NF-κB-conveyed inflammatory cytokine and BACE1 expression. NF-κB and AP1 may also be activated in response to oxidative stress. Importantly, besides enhancing the neuronal Aβ production, JNK and IKKβ directly elicit the insulin-desensitizing Ser-phosphorylation of IRS-1, whereas TANK-binding kinase 1 (TBK1), the upstream activator of IKKβ, was shown to trigger the insulin resistance-associated phosphorylation of IR at Ser^994^ (Munoz et al., 2009; Maldonado-Ruiz et al., 2017; Ojala and Sutinen, 2017; Holscher, 2019). Aβ was further shown to potentiate the TNF-α/PICR-dependent activation of PKR, another IRS-1 Ser-kinase in neurons, to drive IRS-1 phosphorylation and insulin resistance as well as ER stress-associated eIF2α induction and subsequent synaptic damage, LTP impairment and memory deficits *in vivo*. Indeed, the latter adverse effects following i.c.v. Aβ oligomer-infusion were absent in PKR^–/–^ or TNFR1^–/–^ rodents. The same study also suggested that the ER stress provoked in hippocampal neurons by oligomeric Aβ, as evident by enhanced XBP1s, BiP, and CHOP levels, contributed to insulin resistance (Lourenco et al., 2013). Indeed, ER stress, as suppressed by GLP-1 agonists (section “GLP-1 analogues counteract endoplasmic reticulum stress”) (Lourenco et al., 2013), augments insulin pathway desensitization by potentiating inflammatory responses, such as the IRE1α-mediated activation of JNK, PERK, and ATF6 that jointly induce NF-κB-driven pro-inflammatory cytokine production (Hotamisligil, 2010).

Importantly, incretin hormones shield from the negative effects of inflammation on insulin sensitivity. A study that used a mixture of Aβ-based AD models, including hippocampal primary neurons, murine brain slices and monkeys, demonstrated that Exendin-4, in a GLP-1R-dependent manner, diminished the levels of phospho-Ser^312^/ Ser^616^/Ser^636^ IRS-1, enhanced the activating Tyr^465^ phosphorylation of IRS-1 and restored (JNK-associated) axonal transport deficiencies as well as spatial learning and memory retainment (Bomfim et al., 2012). Aβ oligomers were shown to trigger IRS-1 phosphorylation and insulin resistance by stimulating JNK activity (Ma et al., 2009; Bomfim et al., 2012), whereas a GLP-1 analog protected from insulin pathway desensitization in a manner that was dependent on the suppression of JNK as well as the expression and release of TNF-α by hippocampal neurons (Bomfim et al., 2012). As elaborated in section “Inflammation,” GLP-1 mimetics further exert potent anti-inflammatory effects on microglia and astrocytes in both AD and PD models. Therefore, GLP-1 agonists prevent (Aβ-induced) pro-inflammatory cytokine production by neurons and glial cells, resulting in decreased neuronal PICR activation, reduced JNK/IKKβ/PKR-driven Ser-phosphorylation of IRS-1 and, hence, improved insulin pathway sensitivity.

Besides AD, Exendin-4 and a GLP-1R/GIPR dual agonist reversed PD-related and 6-OHDA-induced insulin pathway impairments, including lessened Akt activity, CREB induction and elevated Ser^312^-phosphorylated IRS-1 levels *in vitro* plus lowered Ser^129^ pIRS-1/IRS-1 ratios *in vivo* (Zhang L.Y. et al., 2021). Moreover, Exendin-4 re-sensitized the Insulin/Akt/mTor pathway, as indicated by exosome analysis, in a phase II clinical trial with PD patients, leading to enhanced motor function (Athauda et al., 2017, 2019) (see section “Clinical trials show good protective effects in patients with AD or PD”).

### GLP-1 mimetics rescue insulin resistance/methyl glyoxal-driven neuronal damage in Alzheimer’s disease and Parkinson’s disease

Notably, during T2DM, hyperglycaemia provokes increased AGE formation that, in turn, contributes to systemic oxidative stress, AGE/RAGE-induced inflammation and insulin resistance (Vlassara and Uribarri, 2014). Indeed, all of these harmful alterations during T2DM have been clinically, epidemiologically and pathologically connected to the accelerated progression of AD and PD (Butterfield et al., 2014; De Felice and Ferreira, 2014; Hassan et al., 2020). Post-mortem examinations have confirmed the enlarged presence of cytotoxic AGEs in neurons (Choei et al., 2004; Takeuchi and Yamagishi, 2008), astrocytes and microglia (Takeda et al., 1998) in the hippocampus and parahippocampal gyrus of AD patients as well as the neocortex of individuals with PD (Dalfo et al., 2005). Moreover, AGE-driven oxidative stress exacerbates Aβ expression, while AGE-driven amyloid glycation enhanced Aβ aggregation, α-synuclein crosslinking and Lewy body formation as well as Tau hyperphosphorylation by activating GSK-3β (summarized in Li J. et al., 2012). Notably, AGEs showed a threefold higher binding affinity to ApoE4 compared to the ApoE3 allele, which might be a mechanism that accelerates plaque formation, the development of AD and cognitive decline in ApoE4 carriers (Li and Dickson, 1997).

Both *in vitro* and *in vivo* evidence implies that a decline in glyceraldehyde-3-phosphate dehydrogenase (GAPDH), which is the sixth glycolytic enzyme that converts glyceraldehyde-3-phosphate to 1,3-diphosphoglycerate, enhances the build-up of methyl glyoxal (Figure 2). The latter is a glycating compound that reacts with other proteins and DNA to generate AGEs, as observed during T2DM and hyperglycaemia (Beisswenger et al., 2003; Du et al., 2003; Muronetz et al., 2017). Albeit increased GAPDH activity was reported in a small-scale post-mortem study (Soucek et al., 2003), substantially decreased GAPDH activity and elevated quantities of ROS-inactivated and S-glutathionylated GAPDH were detected in the temporal cortex, inferior parietal lobule or isolated skin fibroblasts of individuals with AD (Kish et al., 1998; Mazzola and Sirover, 2001; Newman et al., 2007). Likewise, nuclear GAPDH was identified in the SNpc of PD patients (Tatton, 2000; Sekar and Taghibiglou, 2020).

As concluded elsewhere, GAPDH forms multimeric complexes with Aβ, while Aβ-associated and Aβ-independent oxidative stress leads to posttranslational disulphide bride formation, nuclear trapping and the enzymatic inactivation of GAPDH (El Kadmiri et al., 2014). Comparably, GAPDH-containing and insoluble paired helical filament Tau was found in the temporal cortex of AD and temporal lobe of Tauopathy patients (Wang et al., 2005; Yang et al., 2008). It is likely that the insulin resistance-associated downregulation of glycolytic enzymes, including GAPDH, and the associated worsening of the Aβ and Tau pathology play a major role in GAPDH dysfunction, subsequent glyceraldehyde-derived AGE formation, oxidative stress and inflammation.

Indeed, the GAPDH/glyceraldehyde-derived AGE methyl glyoxal was demonstrated to be toxic *in vitro* and shown to build up in the cytoplasm of neurons, but not astrocytes, in hippocampal and parahippocampal brain regions of AD patients (Choei et al., 2004). Methyl glyoxal also reacts with dopamine to produce 1-acetyl-6,7-dihydroxy-1,2,3,4-tetrahydro-isoquinaline (ADTIQ) (see Figure 2). The latter not only imitates the effects of the PD-toxin MPTP, but ADTIQ was further heightened in hyperglycaemia-stressed cells and the brains of T2DM rats (Naoi et al., 1997; Song et al., 2014; Hipkiss, 2017). Moreover, ADTIQ was found to amass in the putamen, SNpc, caudate nucleus and other brain areas of PD patients (Naoi et al., 1997; Deng et al., 2012). Synoptically, methyl glyoxal depletes the cellular stores of the anti-oxidant GSH for its detoxification by the glyoxalase system, weakens the mitochondrial membrane potential, enhances the mitochondrial ROS and lessens ATP production, stimulates pro-inflammatory cytokine (IL-1β, TNF-α) expression, induces intracellular dopamine accumulation as well as impairs growth factor (BDNF) pathway signaling in neurons, leading to apoptosis (Di Loreto et al., 2004, 2008; de Arriba et al., 2007; Xie et al., 2014).

*In vitro* studies in high glucose or AGE-stressed neurons imply that GLP-1 or its analogs rescue diabetes-associated structural mitochondrial damage and membrane potential loss, ROS production, DNA oxidation, Aβ secretion, Tau hyperphosphorylation and neuronal death (Li et al., 2010b; Chen et al., 2014; An et al., 2015). Moreover, GLP-1R agonism protected from systemically administered AGEs/methyl glyoxal, leading to reduced hippocampal Tau hyperphosphorylation and apoptosis (caspase 3) induction, re-invigorated PGC-1α activity and mitochondrial biogenesis, enhanced anti-oxidative gene transcription as well as the rescue of synaptic injuries and spatial memory deficits *in vivo* (An et al., 2015; Qi et al., 2017).

More specific for methyl glyoxal, liraglutide ameliorated the mitochondrial membrane potential and survival of SH-SY5Y cells by engaging Akt as well as MEK_1/2_ and its downstream target p90RSK, inhibiting the synthesis of pro-apoptotic Bax and Bik, blocking caspase 3 activity and restoring the decline in anti-apoptotic Mcl-1 levels (Sharma et al., 2014). Furthermore, as shown in methyl glyoxal-stressed PC12 cells, GLP-1 prevents apoptosis by re-establishing the cellular redox balance (GSH vs. glutathione disulfide). This improvement in redox signaling was dependent on the PI3K/Akt/mTor-driven transcriptional upregulation of GCLc, which is the rate-limiting gene in GSH synthesis (Kimura et al., 2009). Since cAMP and MEK inhibitors attenuated the anti-apoptotic effects of GLP-1 in these methyl glyoxal-treated PC12 cells (Kimura et al., 2009), it is implied that the activation of the survival transcription factor CREB, as linked to cAMP/PKA- and MEK_1/2_ /ERK_1/2_/p90RSK-signaling, was involved (see also Figure 2). Indeed, as triggered with 2-iodo-4’-methoxychalcone, a recently discovered GLP-1R agonist, GLP-1R activation enhanced Akt, the inhibitory Ser^9^-GSK-3β and CREB phosphorylation, increased Bcl-2 expression via CREB, reduced the apoptosis-driving Bax, CytC and caspase 3/9 levels, upregulated BDNF and 75*^NTR^*/tyrosine receptor kinase B (TrkB) growth factor receptor synthesis, reduced ROS production and stimulated anti-oxidative defense mechanisms that included enhanced (GSK-3β-suppressed) NRF-2/HO-1 expression, SOD activity and GSH pools in methyl glyoxal-injured SH-SY5Y cells. CHA79 further prevented the decline in glyoxalase-1, which is involved in the detoxification of methyl glyoxal (Tseng et al., 2019).

Notably, in comparison to astrocytes, an *in vitro* study demonstrated that neurons are 6 times more vulnerable to methyl glyoxal due to the weaker expression of glyoxalase enzymes. In this context, astrocytes shield nearby neurons by internalizing methyl glyoxal, followed by the GSH-mediated reduction of the toxic carbonyl compound to hemithioacetal and subsequent processing to D-lactate via glyoxalase-1/2 (Belanger et al., 2011; Allaman et al., 2015). Nonetheless, high methyl glyoxal concentrations eventually enforce AGE generation, glycolytic dysfunction and impaired glutamate uptake even in astrocytes, elevating the neuronal risk of exocytotic injuries and methyl glyoxal-induced anti-oxidant depletion (Hansen et al., 2017). Given that GLP-1 agonists exert various pro-survival effects on astrocytes, such as enhancements in the expression of GSH and multiple neuro-supportive mechanisms (section “GLP-1 exerts direct anti-inflammatory and cytoprotective effects on astrocytes”) (Xie et al., 2021; Zheng et al., 2021), it is likely that GLP-1R activation in astrocytes ameliorates AGE/methyl glyoxal-induced neuronal damage.

## Autophagy and mitophagy

### Treatment with GLP-1 mimetics restores dysfunctional autophagy

Autophagy describes an intracellular cellular clearance process that eliminates waste products, such as misfolded proteins or damaged organelles. There are 3 main forms of autophagy (illustrated in Fujikake et al., 2018) and, in the context of this review, we will specifically refer to the best characterized one: macroautophagy. Briefly, macroautophagy involves the engulfment of junk with autophagosomes that subsequently fuse with lysosomes for the enzymatic degradation of the captured material (Fujikake et al., 2018).

However, as observed during the aging process and exacerbated in AD and PD, the gradual impairment of autophagy is thought to elicit the accumulation and aggregation of toxic waste products and amyloids, such as Aβ, Tau and α-synuclein (Fujikake et al., 2018). Importantly, post-mortem investigations have revealed that AD patients exhibit functional deficits in the lysosomal activity of proteolytic enzymes and the fusion of waste-containing autophagosomes with lysosomes. Thus, the latter impairments lead to the amassment of non-degraded junk, such as lipofuscin inclusions and amyloids, in autophagic vacuoles (Cataldo et al., 1994; Nixon et al., 2005). Importantly, even though the rate of autophagy, as marked by reduced levels of the autophagy-initiating protein Beclin-1, is attenuated during an early stage in AD patients (Pickford et al., 2008), therapeutic approaches to enhance the rate of autophagy only seem to work before degradation-resistant amyloid aggregates have formed (Majumder et al., 2011). Similarly, PD patients exhibited a decrease in autophagy modulators, the accumulation of LC3-II-containing autophagosomes, as indicative of the impaired downstream fusion with lysosomes, the formation of intraneuronal α-synuclein deposits (Chu et al., 2009; Alvarez-Erviti et al., 2010; Dehay et al., 2010) and the sequestration of proteins that regulate the maturation of autophagosomes (LC3, ULK_1/2_ etc.) by these α-synuclein aggregates (Tanji et al., 2011; Miki et al., 2016) in brain regions such as the SNpc. Indeed, it has been revealed that not the deceleration, but the functional impairment of autophagy, triggers neurodegeneration and an AD-like phenotype (Boland et al., 2008).

As reviewed and further discussed in Arden (2018), there is clear evidence that GLP-1 agonists enhance the function of autophagy in various peripheral tissues, which mediates the survival of pancreatic β-cells during hyperglycaemia and T2DM. Similarly, treatment with GLP-1 agonists was shown to prevent T2DM-associated autophagy impairments in the brain *in vivo* (Candeias et al., 2018; Zhang M. et al., 2021).

Our group demonstrated that the application of GLP-1 analogs enhances the transcription of autophagy inducers, including Atg3 and Atg7, and elevated LC3 levels, which implies an increase in autophagosome synthesis, even in non-stressed SH-SY5Y cells (Jalewa et al., 2016). The latter effects were PI3K-dependent (Jalewa et al., 2016), suggesting that the increase in autophagy proteins was driven by the GLP-1R/PI3K/Akt/mTORC1-mediated upregulation of protein translation (Garza-Lombo et al., 2018).

Generally, the mTor pathway regulates macroautophagy in response to the nutritional state. The lack of energy substrates elicits the release of hormones such as Ghrelin that induce the phosphorylation of the energy sensor AMPK, leading to TSC_1/2_ activation, the inactivation of mTORC1 drives autophagy by the ULK_1/2_ initiation complex. In turn, nutrient availability and growth signals, such as insulin or GLP-1, attenuate the induction of autophagy via the PI3K/Akt-mediated suppression of TSC_1/2_ (Garza-Lombo et al., 2018; Reich and Holscher, 2020).

In the context of macroautophagy, Beclin-1, along with vacuolar protein sorting 34 and VPS15, initiate the formation of autophagosomes. Furthermore, Atg4 cleaves immature LC3 at the C-terminus, followed by modification with phosphatidylethanolamine via Atg7 and Atg3 to promote the critical incorporation of LC3 as LC3-II into autophagosomes (Kaleli et al., 2020). While abnormally heightened P62/SQSTM1 levels indicate dysfunctional autophagy, it has been shown that polymeric p62 complexes are necessary for LC3 recruitment, autophagosome maturation and amyloid removal (Bjorkoy et al., 2005).

Despite the fact that GLP-1 mimetics stimulate the PI3K/Akt/mTor pathway and, hence, reduce the rate of autophagy, they were shown to prevent autophagy dysfunction following ER stress and Aβ insult in AD animal models (Figure 1). ER stress, as induced by the accumulation of unfolded proteins and amyloids, oxidative stress or excessive Ca^2+^-amassment in the cytoplasm, impedes autophagy through two mechanisms. This involves the induction of the PERK/eIF2a arm that represses mTORC1-associated protein translation (including that of autophagy-mediators) as well as the activation of apoptosis modulators, i.e., caspase 3 and calpain, which cleave Atg proteins and Beclin-1 to drive suicide, if ER stress persists (Lindholm et al., 2006; Ogen-Shtern et al., 2016; Song et al., 2017). *In vivo* studies indicate that the incretin dual agonist DA-CH3 not only raised Akt-signaling to resolve Aβ-triggered ER stress in the brains of APP/PS1 mice, but also normalized the decreased expression of Atg3, Atg7, Beclin-1 and the autophagosome-associated marker LC3 (Panagaki et al., 2018). Furthermore, i.c.v. Aβ_1–42_-injected rodents developed lipofuscin inclusions in the hippocampus, which was prevented by liraglutide treatment, indicating that the autophagy function was improved (Qi et al., 2016). Similarly, liraglutide rescued the synthesis of Beclin-1 and LC3, with a trend for normalized Atg7, in response to thapsigargin-induced cytosolic Ca^2+^ overload and ER stress in SH-SY5Y cells (Panagaki et al., 2017). Notably, thapsigargin was demonstrated to terminate autophagy by impeding autophagosome synthesis and the fusion of autophagosomes with lysosomes (Ganley et al., 2011; Engedal et al., 2013). As such, the latter results support that GLP-1R activation prevents autophagy impairments in response to Aβ amassment, ER stress and Ca^2+^ deregulation in neurons.

Autophagy-improving effects following GLP-1R activation were also observed in PD models. GLP-1 analogies and GLP-1R/GIPR dual agonists prevented the 6-OHDA-provoked downregulation of autophagy initiators, including Beclin-1 and p62, in neuroblastoma cells (Zhang L.Y. et al., 2021). Further *in vivo* studies in the MPTP and 6-OHDA mouse models showed that the expression of autophagy markers was impaired in nigrostriatal brain areas, whereas the treatment with liraglutide, semaglutide Exendin-4 or GLP-1R/GIPR dual agonists reversed the decline in Beclin-1, Atg7, p62 and LC3-positive autophagosomes (Jalewa et al., 2017; Zhang et al., 2018, 2020; Zhang L.Y. et al., 2021). Strikingly, the Beclin-1 or p62 levels were even doubled in 6-OHDA/DA-JC1-co-injected animals compared to control mice (Jalewa et al., 2017; Zhang L.Y. et al., 2021). As indicative of improved autophagy function and the associated amyloid removal, GLP-1R or GLP-1R/GIPR dual agonists reduced α-synuclein accumulation (Zhang et al., 2019; Lv et al., 2021; Zhang L.Y. et al., 2021) and the deposition of insoluble α-synuclein/p-α-synuclein and ubiquitin inclusions (Yun et al., 2018) in dopaminergic neurons in the brains of various PD *in vivo* models. Indeed, GLP-1 mimetic treatment extended the life span of α-synuclein hA53T Tg mice by 100 days (Yun et al., 2018).

### GLP-1 receptor activation normalizes excessive or impaired mitophagy

Mitophagy is a quality control process and a selective form of autophagy that is implicated in the degradation of dysfunctional and ROS-generating mitochondria. Indeed, the restoration of mitophagy, as deregulated in AD and PD, poses a therapeutic strategy that has been shown to prevent amyloid accumulation and aggregation, brain inflammation, neuronal loss and cognitive impairments (Fang et al., 2019; Liu et al., 2019). Classic Parkin-dependent mitophagy is concertedly executed by PINK1 and Parkin, while mutations in these genes lead to the degeneration of dopaminergic neurons and early onset PD. PINK1 senses mitochondrial damage, including membrane depolarisation and ROS leakage. Upon damage, PINK1 accumulates at the mitochondrial surface and attracts plus phosphorylates cytosolic or inner-mitochondrial Parkin. This results in the polyubiquitination of mitochondrial proteins, autophagy receptor recruitment and the formation of LC3-II-containing autophagosomes to engulf and digest the marked mitochondrion (Liu et al., 2019).

As shown in 3 × Tg AD mice, a GLP-1R/GIPR dual agonist boosted mitophagy by raising the impaired hippocampal PINK1 and Parkin expression (Cai et al., 2021). Notably, the transcription of PINK1 and Parkin is regulated by NRF-1 (Lu et al., 2020), while the expression of NRF-1 is under control of PGC-1α (Xu et al., 2014). GLP-1R agonists restored the expression of NRF-1 upon 6-OHDA-induced mitochondrial damage in neuroblastoma cells (Zhang L.Y. et al., 2021) and in the brains of AGE-injected mice (An et al., 2015), while normalizing the decreased neuronal PGC-1α levels in response to H_2_O_2_, Aβ, or AGE-induced oxidative stress *in vitro* (Ma et al., 2017) and *in vivo* in 3 × Tg AD or AGE-treated mice (An et al., 2015; Cai et al., 2021). Thus, GLP-1R activation prevents mitochondrial damage (section “Oxidative stress and mitochondrial dysfunction”) and the pathology-associated downregulation of transcription factors (PGC-1α/NRF-1; see Figure 2) that synthesize the mitophagy modulators PINK1 and Parkin.

Intriguingly, while enhancing the impeded mitophagy in AD animals (Cai et al., 2021), it was reported that a GLP-1 analog quenched mitophagy-signaling in the nigrostriatal region of MPTP mice (Zhang et al., 2019). Notably, excessive autophagy or mitophagy can promote cell death. It has been suggested that an imbalance between the mitochondrial degradation via mitophagy, as driven by PINK1, Parkin and BNIP3L/Nix, as well as mitochondrial multiplication through biogenesis, mainly induced by PGC-1α, is linked to the development of neurodegenerative diseases. Excessive mitophagy, yet impaired biogenesis, triggers apoptosis through the depletion of mitochondria and ATP, whereas aberrantly enhanced mitochondrial biogenesis in the absence of mitophagy encourages the accumulation of ROS-generating, damaged mitochondria (Palikaras and Tavernarakis, 2014). As indicated in H_2_O_2_-treated RGC-5 cells and confirmed with the autophagy-inducer rapamycin, Liraglutide rescued oxidative stress-induced cell death by suppressing autophagy and normalizing the heightened autophagosome formation (LC3-II/LC3-I conversion ratio) and Beclin-1 expression, while recovering the lowered p62 levels. Furthermore, Liraglutide reduced the mitochondrial injury, potentiated biogenesis (PGC-1α), yet blocked the H_2_O_2_-provoked expression of mitophagy markers [Parkin and BNIP3L/Nix, a Parkin-independent mitophagy inducer (Ma et al., 2017; Liu et al., 2019)]. Comparably, Liraglutide rescued from apoptosis and mitochondrial defects in high glucose/hyperglycaemia-burdened retinal ganglion cells *in vitro* and *in vivo*, respectively, by attenuating autophagy, PINK1/Parkin expression and, hence, mitophagy (Zhou et al., 2020). This implies that the anti-oxidative and mitoprotective effects of GLP-1 agonists (Figure 2) forestall the excessive need of repair mechanisms (autophagy and mitophagy) by protecting from oxidative damage in PD or T2DM/insulin resistance-associated hyperglycaemia. As such, GLP-1 mimetics restore the cellular homeostasis between autophagy/mitophagy and mitochondrial biogenesis.

## Other growth factors

### GLP-1 mimetics stimulate brain derived neurotrophic factor synthesis in neurons and glia

BDNF, which is regarded as the predominant and most widely expressed neurotrophin in the CNS, is a member of the neurotrophin family that encompasses NGF and neurotrophin 3/4. BDNF and its receptor TrkB play a major role in neuroprotection, dendritic branching, synapse formation, neurogenesis as well as plasticity, learning and memory. Interestingly, BDNF shares similar downstream signaling pathways with insulin, IGF-1 and GLP-1 that include the activation of the neuroprotective IRS-1/2/PI3K/Akt pathway that modifies survival modulators, such as Bcl-2 and Bad, and stimulates mTor-dependent protein translation as well as the Ras/Raf/MEK/ERK pathway, which results in CREB-mediated survival, growth, plasticity and *de novo* BDNF gene expression (Sampaio et al., 2017; Holscher, 2019; Lima Giacobbo et al., 2019; Hölscher, 2020). Moreover, BDNF induces the PLC-γ/DAG/PKC pathway implicated in intracellular Ca^2+^ accumulation and the defense from pro-inflammatory (TLR) and pro-apoptotic GSK-3β/p38/caspase 3-signaling (Lima Giacobbo et al., 2019).

Plasma and CNS investigations of the BDNF and TrkB levels in AD patients are inconclusive, showing elevated, lowered or no change in the levels of BDNF or TrkB. However, there seems to be a decline in the cortical levels of BDNF, more apparent in advanced stages of AD, that was also observed in Aβ-based AD rodent models (see Sampaio et al., 2017). It was reported that MCI and AD patients displayed massive reductions in the levels of proBDNF (21% / 30%) and BDNF (34% / 62%) in the parietal cortex that preceded acetylcholine dysfunction and correlated with cognitive impairments (Peng et al., 2005). Such BDNF reductions were seen in the prefrontal cortex, also inversely correlating with cognitive impairments in more advanced AD patients, as well as in Tau-bearing and non-tangle-affected neurons in the frontal cortex of AD patients. Interestingly, strong BDNF expression was discovered in dystrophic neurites that co-localized with plaques, while TrkB was selectively downregulated in Tau-loaded neurons (Ferrer et al., 1999). First, given that neuronal insulin resistance and Tau hyperphosphorylation mutually enhance each other (Goncalves et al., 2019), this suggests that the decrease in neurotrophin receptor expression was caused by impaired insulin-signaling. Second, in cortical neurons, APP-cleavage products, involving Aβ_1–42_, appear to disrupt the anterograde and retrograde trafficking of BDNF-containing vesicles (Seifert et al., 2016), while sublethal concentrations of Aβ_1–42_ block BDNF-signaling (PI3K/Akt and Ras/Raf/MEK/ERK pathways) by interfering with the TrkB-mediated Tyr-phosphorylation of the docking proteins Shc (necessary for Grb2/SOS/Ras-signaling) and IRS-1 (responsible for PI3K and Grb2/SOS/Ras induction) (Tong et al., 2004). Because Aβ interrupts BDNF-signaling, it is likely that early increases in BDNF/TrkB during AD reflect a compensatory response.

BDNF is also expressed in motor areas, such as the basal ganglia or cerebellum, and is, hence, implicated in PD. In this context, BDNF/TrkB-signaling assists the survival of dopaminergic neurons in the SNpc and VTA as well as other nigrostriatal areas, while mediating the synthesis of dopamine D3 receptors and TH (Sampaio et al., 2017). Indeed, as summarized in Sampaio et al. (2017), post-mortem investigations have confirmed a decline in BDNF levels in the SNpc, caudate nucleus and putamen, which was correlated with the magnitude of dopamine neuron death. *In vitro* studies further show that α-synuclein overexpression impairs ERK and PKC, enhances the (Akt-inhibited) GSK-3β activity and blocks the CREB-mediated transcription of BDNF (Yuan et al., 2010).

I*n vivo* studies have demonstrated that GLP-1R, GLP-1R/GIPR, or GLP-1R/GIPR/glucagon receptor agonists enhanced the hippocampal (Park et al., 2021) or neocortical (Ohtake et al., 2014) BDNF expression in WT mice, while restoring BDNF levels in the hippocampus of APP/PS1 mice (Tai et al., 2018), 3 × Tg and 5xFAD AD animal models (Park et al., 2021) as well as the SNpc and striatum of MPTP-treated rodents (Ji et al., 2016; Lv et al., 2021). Exendin-4 further recovered the downregulated CREB and BDNF synthesis in the hippocampus of a T2DM animal model (Gumuslu et al., 2016). Lastly, GLP-1 stimulated the expression of BDNF, but also GDNF and NGF, in BV-2 and primary adult microglia in a PI3K and PKA-dependent fashion (Spielman et al., 2017). Similarly, GLP-1R activation on astrocytes preserved the astroglial BDNF secretion following Aβ assault by inducing the cAMP/PKA/(CREB) pathway (Xie et al., 2021).

Mechanistically, an *in vitro* study demonstrated that GLP-1R activation and downstream PI3K/Akt, ERK, and cAMP/PKA-signaling jointly elicit the phosphorylation of the transcription factor CREB to increase BDNF promotor activity and expression (Figure 2). Moreover, while the withdrawal of differentiation agents and neurotrophins from the culture medium led to the differentiation of human neuroprogenitor cells toward GFAP-positive astroglia, the application of Exendin-4 dose-dependently elevated BDNF secretion and preserved the neuronal phenotype, including the presence of neuritic and synaptic markers such as synaptophysin and MAP2a (Velmurugan et al., 2012).

Notably, oxidative stress and the formation of lipid peroxidation products was shown to reduce CREB expression, CREB phosphorylation and the CREB-mediated transcription of BDNF due to the induction of the JNK/c-Jun stress pathway and the competitive BDNF promotor binding by c-Jun (Zhang and Jope, 1999; Pugazhenthi et al., 2006; Fu et al., 2019). Given the anti-oxidative capacities of GLP-1 analogs (see Figure 2 and section “Oxidative stress and mitochondrial dysfunction”), this is another mechanism that preserves BDNF expression.

Another trigger of BDNF impairments is inflammation. For example, Aβ_1–42_-provoked decreases in the cerebral BDNF levels could be rescued with the anti-inflammatory cytokine TGF-β1 (Prakash and Kumar, 2014; Chen et al., 2015), whereas reduced BDNF transcription co-occurred with elevated IL-6 and TNF-α mRNA levels as well as reactive microglia numbers in the SNpc, striatum and hippocampus of PD and Lewy Body Disease patients (Sawada et al., 2006). IL-1β was also shown to interrupt BDNF-signaling by eliciting the inactivating phosphorylation of IRS-1, a downstream mediator of both IR and TrkB (Tong et al., 2012). The release of inflammatory modulators by microglia and astrocytes, such as IL-1β, IL-6, and TNF-α, activate the corresponding PICR on neurons, resulting in the GSK-3β-driven inhibition of CREB-mediated BDNF expression (Grimes and Jope, 2001; Gould et al., 2004), pro-apoptotic p38/caspase 3-signaling and the potentiation of NF-κB-conveyed inflammatory gene transcription (Lima Giacobbo et al., 2019). As such, it is implied that the anti-inflammatory effects following GLP-1R activation (section “Inflammation”) prevent inflammation-triggered impairments in the signaling pathway and the expression of BDNF.

### GLP-1 analogs enhance GDNF expression in dopaminergic neurons

GDNF synthesis occurs in a few selected brain regions, including the dorsal and ventral striatum or SNpc, whereas GDNF family receptor α subtypes (GFRα1-4) are distributed across the CNS. GDNF shows high affinity for GFRα1, which further leads to the co-activation of the receptor Ret. While GFRαs are seemingly absent in the striatum, they are densely expressed by nigral dopaminergic neurons. Therefore, GDNF is considered to be the most crucial neurotrophic factor in the midbrain dopaminergic system. Mechanistically, GDNF/GFRα1/Ret-signaling leads to activation of the neuroprotective Gab_1/2_/PI3K/Akt and Grb2/SOS/Ras/Raf/MEK/ERK pathways to modulate the neuronal survival, differentiation, maintenance and organization. Presumably due to its cell-specific actions, GDNF is 5–10-fold more potent than BDNF in the rescue of nigrostriatal dopaminergic neurons, which has made GDNF the pursued therapeutic approach for PD. Indeed, PD patients have been shown to exhibit selective and grave reductions in the levels of GDNF in the SNpc (see Kramer et al., 2015; Pramanik et al., 2017; Sampaio et al., 2017). Moreover, α-synuclein accumulation was shown to interfere with GDNF-signaling in nigral dopaminergic neurons by reducing the Nurr1-induced transcription of the GDNF receptor Ret (Decressac et al., 2012). However, despite having high potential, the cerebral GDNF infusion in clinical PD trials failed to deliver benefits and led to severe side effects. It has been postulated that viral strategies to selectively increase GDNF expression in nigrostriatal regions, improved infusion protocols or the co-treatment of the Ret/GDNF-inhibiting α-synuclein pathology are necessary (Pramanik et al., 2017).

Similar to BDNF, a range of studies has confirmed that single GLP-1 or dual-activating GLP-1/GIP incretin analogues stimulate the phosphorylation of Akt and CREB (Jalewa et al., 2017) to rescue GDNF expression in the SNpc and striatum of MPTP-microinjected PD rodents (Jalewa et al., 2017; Yuan et al., 2017; Feng et al., 2018; Zhang et al., 2019; Lv et al., 2021). A recent study showed that both the expression of BDNF and GDNF in nigral dopaminergic neurons is mediated by phospho-CREB and dependent on the (Akt-associated) induction of mTORC1-driven protein translation (Figure 2; Nam et al., 2015). Given that α-synuclein intercepts Ret/GDNF-signaling in dopaminergic neurons (Decressac et al., 2012), while GLP-1R activators were shown to prevent the accumulation of soluble, insoluble and phosphorylated α-synuclein species in PD *in vivo* models (Yun et al., 2018; Zhang et al., 2019; Lv et al., 2021; Zhang L.Y. et al., 2021), GLP-1 mimetics might be a safer, easier administrable and more effective alternative to endogenous GDNF treatment.

## GLP-1 receptor agonists promote neurogenesis

Albeit at a low rate, in rodents, neurogenesis occurs across adulthood and describes the birth, proliferation and differentiation of neural stem cells toward functional neurons. Neural stem cells are exclusively found in two specialized brain areas: the subgranular zone of the DG as well as the subventricular zone (SVZ). DG-derived neuroblasts may integrate as mature glutamatergic granule cells into the hippocampal formation to support memory, whilst SVC-originating neuronal precursor cells can migrate to the olfactory bulb (Apple et al., 2017). There is also evidence that DG immature neurons improve information resolution in the hippocampus, with higher numbers of newborn neurons facilitating pattern separation and declarative memory (Aimone et al., 2011). Unsurprisingly, neurogenesis not only declines with age, but is impeded during AD and PD. Therefore, some therapeutic approaches aim to boost neurogenesis (Van Bulck et al., 2019).

Murine *in vivo* studies showed that GLP-1Rs are expressed by adult neural progenitor cells of the SVZ (Bertilsson et al., 2008). Likewise, GLP-1R expression was observed in granular neurons and polymorphic cells of the DG, although the receptor levels on DG granule cells appear to significantly decline during early adulthood (Lee et al., 2011b).

As proof of concept, the injection of various GLP-1 or GLP-1/GIP mimetics enhanced the numbers of doublecortin (DCX)-displaying immature neurons in the SVZ (Salles et al., 2018, 2020), the nearby medial striatum (Bertilsson et al., 2008) or DG (Li et al., 2010a; Hamilton et al., 2011; McClean et al., 2011, 2015; Hunter and Holscher, 2012; McGovern et al., 2012; Parthsarathy and Holscher, 2013a; McClean and Holscher, 2014a,b), increased neuroblast differentiation [bromodeoxyuridine (BrdU) and Ki67] (Li et al., 2010a; Hamilton et al., 2011; Hunter and Holscher, 2012; McGovern et al., 2012; Parthsarathy and Holscher, 2013a) and heightened their differentiation into adult neurons (NeuN) (Parthsarathy and Holscher, 2013a) in the brains of WT, APP/PS1 or various T2DM animal models. Liraglutide failed to induce neurogenesis in the DG in APP/PS1 mice in another study, however (Holubova et al., 2019). Given that GLP-1 mimetics improved the birth of neurons in WT mice as well, this suggests that neurogenesis was enhanced as a consequence of GLP-1R activation and not only through the improvement of the brain pathology. Besides *in vivo* studies, neurosphere cultures derived from the SVZ that were cultured on a liraglutide-disposing biodevice showed an increased birth of DCX-positive neurons as well as signs of differentiation (Salles et al., 2018). Similarly, GLP-1 and exendin-4 enhanced the neuronal proliferation, as assessed via BrdU and ATP levels, and the expression of the differentiation markers MAP2, β-III-tubulin and neuron-specific enolase in SVZ-derived neurospheres and the rat embryonic striatal ST14A stem cell line (Bertilsson et al., 2008).

The available evidence indicates that GLP-1 mimetics possess neurotrophic capabilities, enhancing the proliferation, differentiation and maturation of immature neurons in the DG and SVC. Explicitly, GLP-1 analogs were shown to induce PI3K/Akt and cAMP/PKA/CREB-signaling, resulting in a PI3K- and PKA-, but not ERK-, dependent and dose-related improvement in the viability and proliferation of SH-SY5Y neuroblastoma cells (Qin et al., 2008; Li et al., 2010c,^2015^). Notably, in the APP/PS1 mouse model of AD, progenitor neuron proliferation in the DG (as measured by BrdU) begins to be impaired at an age of around 3 months, when oxidative stress and plaque formation start to incline, whilst the total quantities of DCX-positive immature neurons decreased from an age of 10 months (Hamilton and Holscher, 2012). Moreover, GSK-3β, as inactivated by Akt and overactive during AD, led to growth cone collapse and neurite retraction *in vitro* (Sayas et al., 1999) as well as postsynaptic protein (PSD-95) reductions, GluR1 loss and dendritic shortening in immature DG neurons plus microgliosis and inflammatory cytokine expression in the DG *in vivo* (Llorens-Martin et al., 2013). Given the pro-growth [PI3K/Akt/mTor pathway (Garza-Lombo et al., 2018)], anti-oxidative (section “Oxidative stress and mitochondrial dysfunction”), anti-amyloid (section “amyloid beta and Tau pathology in AD”) and GSK-3β-inhibiting (section “GLP-1R agonists suppress amyloid beta and GSK-3β-driven mitochondrial damage in AD”) effects of incretins, early GLP-1 treatment both stimulates growth and prevents proliferation deficits in immature neurons.

Interestingly, GLP-1 preserved the neuronal phenotype of isolated human neuroprogenitor cells even after withdrawal of other differentiation-boosting agents (i.e., retinoic acid, EGF and FGF), albeit the neuronal differentiation was less efficient (Velmurugan et al., 2012). As indicated in another study using PC12 cells, GLP-1 seems to potentiate the differentiation-enhancing effect of other neurotrophins (NGF in this case) (Perry et al., 2002b). Indeed, when GLP-1 was applied to SH-SY5Y cells following initial retinoic acid treatment, the incretin hormone attenuated vimentin levels (a marker of proliferating neural progenitor cells) and induced AMPA, NMDA, and dopamine receptor and synaptic protein expression, neurite outgrowth, the development of neuron-like morphologies and the ability to generate action potentials (Yang et al., 2020). Furthermore, amongst other effects, GLP-1R induction enhanced Na^2+^ and L/T-type VDCC currents, paralleling those of mature neurons (Luciani et al., 2010).

The use of pharmacological inhibitors demonstrated that the GLP-1-boosted growth, differentiation toward glutamatergic/dopaminergic neurons and survival were dependent on the PI3K/Akt/CREB axis and partially on MEK (Yang et al., 2020). Other studies indicate that GLP-1R-induced Ras/Raf/MEK/ERK-signaling, as confirmed with MEK inhibitor treatment of PC12 cells, drive GAP-43 expression as well as GAP-43-associated neurite outgrowth and branching (Liu et al., 2006). The neurite number and length-boosting effects of Exendin-4, as observed in cultured adult sensory neurons and SH-SY5Y cells (Luciani et al., 2010; Kan et al., 2012), involved concomitant cytoskeletal adaptions, including an increase in F-actin and tubulin polymerisation as well as the phosphorylation and inactivation of the depolymerising factor cofilin (Luciani et al., 2010). Therefore, GLP-1 induced PI3K/Akt-signaling supports the survival and proliferation of immature neurons, whereas the Ras/Raf/MEK/ERK pathway elicits neurite outgrowth.

In addition, incretin hormones stimulate the differentiation of progenitor neurons across the CREB/BDNF axis. *In vitro* studies confirm that GLP-1R activation evokes CREB-phosphorylation via the PI3K/Akt, Ras/Raf/MEK/ERK and cAMP/PKA pathways, even under unstressed conditions (Perry et al., 2002b; Velmurugan et al., 2012), leading to the expression of BDNF and the differentiation toward a neuronal phenotype (Velmurugan et al., 2012). It is widely accepted that the local, CREB-mediated expression of BDNF in the hippocampus (without any external BDNF sources from the blood) is mandatory for memory formation and neurogenesis. Indeed, CREB expression co-occurs with that of DCX and seems to terminate with the emergence of adult neuron markers in mature granule cells, such as NeuN. Moreover, CREB enhances neurite outgrowth, dendritic spine formation and functions as a pivotal survival factor in newborn neurons, responsible for the transcription of key modulators such as the anti-apoptotic protein Bcl-2 or the neurogenic factors BDNF and NGF (Ortega-Martinez, 2015). In this context, BDNF was shown to be expressed and secreted across dendrites by mature granule cells of the DG, followed by the induction of TrkB on GABAergic interneurons. The latter neurons subsequently promote the differentiation and maturation of neighboring neuroblasts as well as their DG integration as mature neurons (Waterhouse et al., 2012). Importantly, GLP-1R or GLP-1R/GIPR dual agonists were shown to enhance CREB activity in various non-pathologic or pathologic contexts *in vitr*o (Velmurugan et al., 2012; Yang et al., 2016), in adult mice (Ohtake et al., 2014) as well as AD (Cai et al., 2018; Li et al., 2020a), PD (Jalewa et al., 2017; Zhang L.Y. et al., 2021), and T2DM (Gumuslu et al., 2016) animal models *in vivo*. Moreover, GLP-1R activation led to the upregulation of BDNF in the hippocampus and other brain areas of WT and the aforementioned rodent models of neurodegeneration (section “GLP-1 mimetics stimulate BDNF synthesis in neurons and glia”) (Ohtake et al., 2014; Gumuslu et al., 2016; Ji et al., 2016; Tai et al., 2018; Lv et al., 2021; Park et al., 2021).

Besides neurons, glial cells contribute to neurogenesis through the hippocampal supply with BDNF (Quesseveur et al., 2013). Similar to neurons, by inducing the PI3K/Akt and cAMP/PKA pathways, GLP-1 mimetics facilitate the glial delivery of neurotrophic factors by improving the microglial production and release of BDNF, GDNF, and NGF (Spielman et al., 2017), while preventing the loss of the astroglial BDNF secretion in response to Aβ (Xie et al., 2021).

Notably, neuroinflammation and the release of pro-inflammatory cytokines (i.e., TNF-α), but not amyloidosis per se, are thought to be the major cause of neurogenesis impairments. This discovery is supported by fact that the treatment with various anti-inflammatory agents rescued neurogenesis in AD and PD-like animals (Ghosal et al., 2010; Le Grand et al., 2015). It is likely that neuroinflammatory processes impair neurogenesis by interrupting BDNF-signaling and the transcription of BDNF (Sawada et al., 2006; Tong et al., 2012; Prakash and Kumar, 2014; Chen et al., 2015; Lima Giacobbo et al., 2019). Given the potent direct and indirect anti-inflammatory effects of GLP-1 in glial cells and neurons (section “Inflammation”), it can be postulated that the administration of GLP-1 analogs maintains neurogenesis in light of age- and pathology-associated neuroinflammation.

## Clinical trials show good protective effects in patients with Alzheimer’s disease or Parkinson’s disease

Based on the protective effects of GLP-1 receptor agonists on a wide range of physiological systems described above, several clinical trials have been conducted and have shown good effects. The results of four clinical trials in AD or PD have been conducted, testing the GLP-1 receptor agonists exendin-4 or liraglutide. These trials have shown clear and impressive effects that improve clinical pathologies of PD and AD. These results are a clear proof of concept that the use of such growth-factors are indeed effective and show meaningful effects.

### Exendin-4 (exenatide)

In a single-blinded, open-label pilot study that tested the drug exendin-4 (NCT01174810) (viles-Olmos et al., 2013), 45 patients with moderate PD who were on L-Dopa-based treatment were randomized to either exendin-4 (Byetta) twice daily treatment for 12 months, or were controls without treatment. Drug treatment was for 12-month, followed by a 2-month “drug wash-out” period where no drugs were given to test if the drug effect is still visible. After 14 months, the result showed significant differences. A significant improvement was found in the Mattis dementia rating scale-2 (Mattis DRS-2) which tests the PD-associated cognitive impairments. Furthermore, a clear improvement in motor control was observed in the Movement Disorders Society Unified PD Rating Scale (MDS-UPDRS) part 3 in the drug group. The drug group did not deteriorate, while the control group showed steady and strong decline in all measures (viles-Olmos et al., 2013). The motor test battery MDS-UPDRS part 3 had been conducted by Neurologists blind to the treatment. Importantly, the benefits in both motor and cognitive abilities were still visible in follow-up tests 12 months after the last drug treatment (viles-Olmos et al., 2014). Again, the drug group did not deteriorate after 24 months in cognitive and motor assessments, while the control group had deteriorated severely. This demonstrates that the improvements in motor control and cognitive tests are not due to a placebo effect.

Based on these encouraging results, a follow -up phase II clinical trial with a placebo control group had been run (NCT01971242) (Athauda et al., 2017). The once-weekly formulation of exendin-4 (Bydureon) was tested in PD patients for 60 weeks, accompanied by a placebo group. In this study, 60 patients with moderate PD were treated with exenatide (Bydureon; once weekly) or placebo for 48 weeks in addition to their dopaminergic replacement drugs. This was followed by a “wash-out” period of 12-weeks. Patients that had received the drug showed far better values in the part 3 of MDS-UPDRS motor tests than the placebo group following 48 weeks of exenatide therapy. As in the pilot study, the drug group did not deteriorate, while the placebo group did. After 12 weeks of no-treatment, the difference between groups was still visible. CSF analysis demonstrated that exendin-4 can cross into the brain, and furthermore showed that it was no longer present after wash-out (Athauda et al., 2017). The improvements in MDS-UPDRS part 3 scores were still visible after “wash-out” at 60 weeks, demonstrating that the drug effect is not an simply an acute effect, satisfying the definition of disease-modification. In addition, a range of additional secondary measures was assessed, most of which showed an improvement (Athauda and Foltynie, 2018; Foltynie and Athauda, 2020).

Exosomes are vesicles that are released from cells. Neuronal exosomes taken from blood samples from patients that took part in this clinical trial showed improvements in insulin signaling in the brain, similar to GLP-1 drug effects in diabetes. It was found that standard biomarkers of insulin signaling such as IRS-1 phosphorylation at tyrosine sites was improved in the brains of PD patients. This demonstrates that the preclinical research results translate into the clinic (Athauda et al., 2019; Yang X. et al., 2022).

### Liraglutide

A randomized, double-blind phase II clinical trial showed that liraglutide can improve PD pathology (NCT02953665). PD patients received once-daily subcutaneous injections of liraglutide for 52 weeks in addition to standard clinical medication to treat PD. The analysis included 37 active and 18 placebo subjects. At 54 weeks, non-motor symptom scores (NMSS) had improved in the liraglutide group and worsened in the placebo group, a 13.1 point adjusted mean difference (*p* = 0.07), just missing statistical significance. However, a significant improvement in MDS-UPDRS part-II scores in the treatment group had been observed (*p* = 0.001). This test battery evaluates everyday activities such as walking, talking, eating, getting dressed, getting out of a car, tremor, personal hygiene activities, and more (Malatt et al., 2022). Furthermore, statistically significant improvement of Global MDS-UPDRS and PDQ-39 (quality of life) scores were found. The result shows that liraglutide improves PD pathology beyond the effects of L-Dopa.

We have conducted a placebo- controlled double-blind phase II clinical trial testing liraglutide in over 200 MCI/AD patients for 1 year (ELAD study, NCT01843075). It analyzed the effects on cognition (ADASexec tests), brain volume changes as measured by MRI brain scans, and other parameters that are still being analyzed (Femminella et al., 2019). It was found that liraglutide protected patients from cognitive impairment as measured in ADASexec tests (*p* < 0.01). In MRI brain scans, brain temporal lobe volumes shrank less in the drug group compared with the placebo group (*p* < 0.001), and the total gray matter cortical volume shrank less in patients, too (*p* = 0.002), indicating that neuronal loss has been reduced by the drug (Edison et al., 2020, 2021; Hölscher, 2020).

Other clinical trials testing GLP-1 type drugs in PD patients are currently ongoing, testing the drugs semaglutide (NCT03659682), lixisenatide (NCT03439943), NLY01 (NCT04154072), or PT320 (NCT04269642) in PD patients, with additional phase 2 (NCT04305002) and phase 3 (NCT04232969) trial testing exenatide in PD patients, highlighting the growing interest and demand for discovery and development of GLP-1R agonists. Two phase III clinical trial testing semaglutide in AD patients are currently underway, too (NCT04777396 and NCT04777409).

### Future challenges

The first clinical trials in AD and PD show clear neuroprotective effects, with others reporting later this year. Phase 3 trials have started, demonstrating the confidence and interest of the industry in this novel approach. In addition, dual GLP-1/GIP receptor agonists are being developed as novel treatments for AD and PD (Hölscher, 2022b; Yang X. et al., 2022). As current GLP-1 receptor agonists have been developed to treat diabetes, it is important to design drugs specifically for treating CNS diseases. Diabetes drugs have been developed to remain in the blood stream for a long time, ensuring protection from hyperglycaemia day and night (Hedrington et al., 2018; Samms et al., 2020). However, this means that the drugs do not enter the brain very well, reducing target engagement (Salameh et al., 2020; Yang X. et al., 2022). There is a clear correlation between the neuroprotective potency of drugs and their ability to cross the BBB, as demonstrated by growth factors such as NGF, BDNF, and GDNF. While showing excellent neuroprotective effects in cell culture or when injected directly into the brain, they do not have any effects when injected peripherally (Allen et al., 2013). Hence, novel dual agonists have been developed to cross the BBB at an enhanced rate, showing superior effects in animal models compared with GLP-1 receptor agonists that have been developed to treat diabetes (Yang X. et al., 2022). Further improvements can be made by adding other receptor agonist binding sites to the peptide, with GLP-1 of GIP dual agonists only just starting this area of drug discovery. The future looks bright for this novel research area, and we may well be at the beginning of a paradigm shift in treating chronic neurodegenerative disorders such as AD and PD.

## Conclusion

The peptide hormone and growth factor GLP-1 plays key roles in cell growth, energy utilization, autophagy, protection from oxidative stress and in the control of chronic inflammation responses. Neurodegenerative disorders such as AD and PD are driven by chronic inflammation and impairments in growth factor signaling. Reversing this impairment by the use of GLP-1 mimetics has shown improvements in cell growth and repair, and in the re-activation of synaptic activity and energy utilization, while reducing the chronic inflammation response and removing misfolded protein build-up. Neuronal survival and functionality is rescued, and key pathological markers such as motor impairment, loss of the striatal-dopaminergic function and neuronal loss in PD, and loss of glutamatergic neurons and cognitive processes in AD are markedly improved in preclinical studies as well as in first clinical trials in AD and PD patients. These encouraging results demonstrate the big potential that the pharmacological treatment with GLP-1 mimetics holds.

## Author contributions

NR wrote the manuscript and made the figures. CH reviewed and revised the manuscript. Both authors contributed to the article and approved the submitted version.
